# Is it possible to optimise the labour and time intensity of diatom analyses for determination of the Polish Diatom Indices (IO, IOJ)?

**DOI:** 10.1007/s10661-022-10676-7

**Published:** 2022-11-03

**Authors:** Adrian Kryk, Małgorzata Bąk, Aleksandra Kaniak, Marzena Adamczyk

**Affiliations:** 1 Institute of Marine and Environmental Sciences, University of Szczecin, Mickiewicza 16, 70-383 Szczecin, Poland; 2 West Pomerania Agricultural Centre in Barzkowice, Barzkowice 2, 73-134 Barzkowice, Poland

**Keywords:** Diatom valve counting, Reduction in counted valves number, Methodology optimisation, Polish diatom index for rivers, Polish diatom index for lakes, River Ina, River Drawa

## Abstract

Diatom-based indices are a recommended approach in the assessment of the ecological status of lakes and rivers, required by the EU Water Framework Directive. To optimise the time-consuming microscopic analyses, we determined the lowest number of diatom valves needed to be identified (‘counted’) in a sample to arrive at a reliable ecological status assessment based on the Polish multimetric diatom indices (diatom index, IO for rivers, and diatom index for lakes, IOJ). We compared the indices calculated with different valve counts. The dataset consisted of diatom counts in 55 samples collected in the rivers Ina and Drawa (Western Pomerania, NW, Poland) and their lakes. Following the method used by the Polish Chief Inspectorate for Environmental Protection (ChIEP), periphyton samples were collected from macrophytes growing in the rivers and lakes, and diatom slides were prepared. Originally, the ecological status was determined based on identifying 400 valves per slide. In the subsequent simulation, 50, 100, 150, 200, 250, 300, 350 and 400 valves per slide were randomly drawn 20 times for each sample and each simulation, and the indices were calculated again. In 87% of our simulations, the ecological status turned out to be identical, regardless of the number of valves identified. However, the standard deviation of the index values was at its highest (> 0.05) with 50 valves, and decreased systematically (below 0.05) for 100 and more valves. Simulated datasets showed logarithmic trend of changes where the compilation and standard deviation was stable above 200 valves identified. Therefore, we recommend simplified methodology for Polish multimetric diatom indices. Reduction of identified valve quantity in a sample to 200 valves would result in over 95% of compliance with classification obtained when 400 valves were identified.

**Supplementary Information:**

The online version contains supplementary material available at 10.1007/s10661-022-10676-7.

## Introduction

Research on the quality of European waters has intensified after the European Union’s Water Framework Directive became operative (European Council, 2000). The Directive puts forth biological parameters as the most important indicators of the quality of water bodies.

Among numerous biotic indicators, those based on diatoms have been increasingly frequently considered the most reliable. This approach has a long history. Diatoms have been used as indicators of riverine water since the early 1900s (e.g. Bąk et al., 2012; Coring, 1999; Ector & Rimet, 2005) as they are well-studied, cosmopolitan, sensitive to physico-chemical changes in the water and therefore responding very rapidly to environmental changes, even slight ones, e.g. changes in the intensity of eutrophication, acidity, saprobity, nitrogen content, salinity and current speed (e.g. Battarbee et al., 1997; Coring, 1999; Denys, 1991a, b; Kelly et al., 1998; van Dam, 1996). Moreover, diatoms are easy to sample and can be archived, on permanent slides, for later examination and possible taxonomic revisions.

Data on diatom assemblages in different regions, water types and micro-habitats have been presented and compared in numerous studies. However, relatively little is known on the effects of methodological differences, particularly with respect to the number of diatom frustules (valves or cells) identified (‘counted’) and hence the precision of diatom composition assessment (Besse-Lototskaya et al., 2006; Stoermer & Smol, 2001). A diatom assemblage data set consists of counts of individual diatom taxa identified in one or more samples. The goal of counting representatives of diatom species in a sample is to obtain semi-quantitative data from which statistically valid ecological interpretation can be drawn and, more importantly, to extract information on the dominant species to quantify their occurrence in the natural habitat as precisely as possible (Patrick, 1973) and, hence, to be able to assess the status of that habitat. To this end, the relative abundance of the diatom taxa present in a sample is converted to a metric (index) reflecting the habitat status. The diatom-based habitat quality indices include the following: the Indice de Polluo-sensibilité Spécifique (IPS; Cemagref, 1982), the Generic Diatom Index (GDI; Coste & Ayphassorho, 1991), the Trophic Diatom Index (TDI; Kelly & Whitton, 1995), the Indice Biologique Diatomeé (IBD; Lenoir & Coste, 1996), the Eutrophication/Pollution Index based on Diatoms (EPI-D; Dell’Uomo, 1996), the Indice Diatomique Artois-Picardie (IDAP; Prygiel et al., 1996), the Leclercq and Maquets Index (LMI; Leclercq & Maquet, 1987), Sládeček’s Index (SLA; Sládeček, 1986), and Descy’s Index (DES; Descy, 1979). Many of those indices are being widely used around the world (Chessman et al., 2007; Feio et al., 2009; Jüttner et al., 2003). Moreover, there are a great number of regional indices that have been developed to solve specific environmental problems or to be applied to a specific country or region. These include, for example, the acidity index for diatoms (ACID; Andrén & Jarlman, 2008; Kahlert & Andrén, 2005) applied in determining the acidity of Swedish rivers; the Eastern Canadian Diatom Index (IDEC; Lavoie et al., 2006) used in some parts of Canada; the Pampean Diatom Index (PDI; Gomez & Licursi, 2001) applied to water quality assessments in Argentina; the Swiss Diatom Index for Switzerland (DI-CH; Hürlimann & Niederhauser, 2006); and the multimetric diatom indices IO and IOJ, both used in Poland in monitoring of rivers and lakes, respectively (Picińska-Fałtynowicz & Błachuta, 2010).

Despite their utility, the diatom-based indices come also with some disadvantages, such as the need-to-know ecological preferences of each diatom taxon, and to have reference species for all types of water bodies. Moreover, diatom identification can be very costly and time-consuming; it should be performed by well-trained taxonomists (Bąk et al., 2012; European Standard, 2004; Szczepocka & Żelazna-Wieczorek, 2018; Szczepocka et al., 2014). Reducing a cost of water quality assessments is in interest of authorities responsible for environmental monitoring. Methodological recommendations which can lower the cost of diatom-based water quality assessment without losing its reliability are crucial to optimise methodological procedures. Therefore, there have been efforts to optimise the procedures by analysing the enumeration approaches used (Brabcová et al., 2017; Tyree et al., 2020), taxonomic resolution level (Raunio & Soininen, 2007; Rimet & Bouchez, 2010, 2012), sampling effort (Bennett et al., 2014) and exclusion of rare taxa (Lavoie et al., 2009). However, there are no such methodological analyses concerning IO and IOJ indices, both used in Poland. Therefore, the aim of this study was to test a methodological simplification for the Polish multimetric diatom indices.

Actual methodological guide for Polish diatom indices requires a minimum of 400 valves to be counted in a sample (Picińska-Fałtynowicz & Błachuta, 2010). A particularly useful question in this context is whether the values of IO and IOJ calculated with fewer diatom valves counted in a sample would result with the same water quality classification as when counted up to recommended 400 valves in a sample? Such reduction would successfully reduce microscopic work time and thus analysis cost. To answer the question, effects of reducing diatom valves to be identified (‘counted’) in a sample were assessed by simulating various counting sums. In order to work on a larger dataset, two datasets, both from different river systems but from the same region, were tested in this manner. This work compares results of ecological status assessment of 2 rivers and 10 lakes. We use the results of the comparison to estimate the valve count required for a reliable ecological status assessment and to indicate the applicability of our inferences. The final aim of this study was to develop reliable recommendation which would simplify methodology used for IO and IOJ calculation and result in identical water quality assessment. Reduction in count size could led to more effective in costs environmental monitoring.

## Material and methods

### Data sources and acquisition

**Fig. 1:**
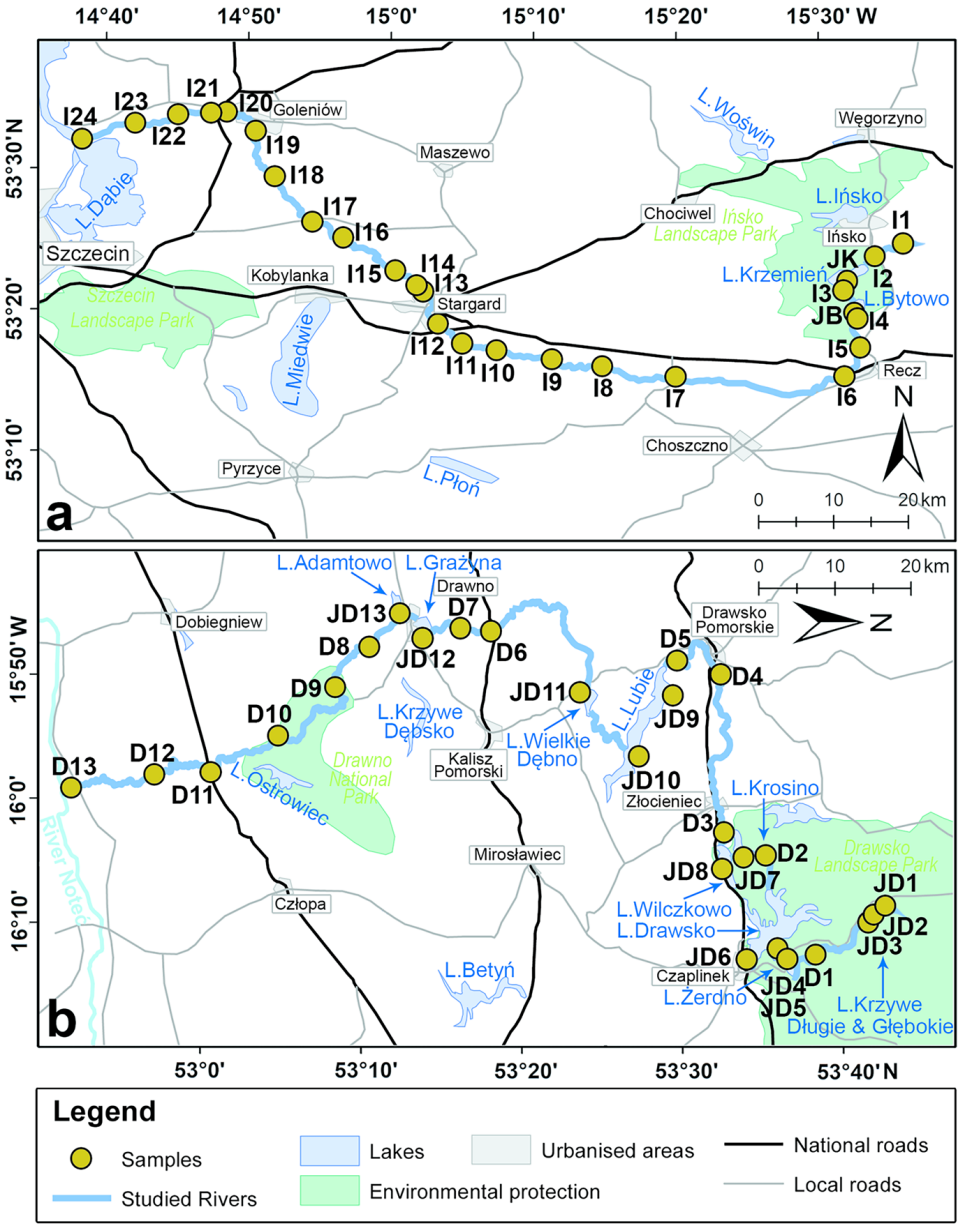
Map of two river systems and location of taken samples: **a** Ina River; **b** Drawa River

Our dataset consists of 54 samples collected from two NW Poland River systems which had been previously analysed during the original research in 2013. The original research concerned the ecological assessment of the above river systems and was conducted as part of authors’ degree theses preparation. Identification was made by 2 observers, each working on one river system (AK* & MA). As the original research was a part of degree theses, to minimise the observer’s bias, each diatom species identified by an observer was verified by a supervisor (MB). Unpublished data of diatom taxonomical composition from those studies were re-used for this paper purposes. The first system is that of River Ina with lakes Krzemień and Bytowo, the other system consisting of River Drawa with its eleven lakes: Adamtowo, Grażyna, Wielkie Dębno, Lubie, Krosino, Wilczkowo, Drawsko, Żerdno, Głębokie, Długie and Krzywe (Fig. 1). The samples were collected along the course of the Ina and the Drawa (26 and 13 samples, respectively) as well as from the lakes (a total of 15 samples). All the samples were collected between 19 and 21 October 2013. Wherever possible, the sampling sites were situated about 5–10 km away from each other along the entire length of each river (Fig. 1). As the water quality assessment is recommended to be based on diatoms attached to submerged surfaces, where on lowland rivers such as those used in this study the most suitable surface is periphyton (Lampert & Sommer, 2007; Picińska-Fałtynowicz & Błachuta, 2010), samples were collected from macrophytes (mainly *Phragmites australis*) by cutting off, with scissors, a shoot fragment at a depth of about 20 cm below the water surface and scraping the periphyton off. The scraped material was transferred to a plastic container with water (about 100 ml) and the sample number, GPS location (Supplementary material 1) and general characteristics of the environment were recorded. The samples are numbered in an increasing order from the upstream reaches of the river to its mouth.

The material was cleaned with 10% HCl and 37% H_2_O_2_ (Battarbee, 1986). Thereafter, cleaned material was pipetted on cover slips, left at room temperature until the water evaporated and mounted on glass slides using Naphrax®. In compliance to the standard methodology for diatom analyses, at least two permanent slides were made with different valve densities and the one regarded as optimal was used in microscopic analyses (Bąk et al., 2012; Battarbee, 1986).

The permanent diatom slides were examined under a Nikon Eclipse E600 light microscope with a PlanAPO × 100 immersion lens at × 1000 magnification. In each sample, diatoms were identified to the lowest taxonomic level possible, most often to the species, and occasionally to the subspecies, variety or form, and less often to the genus. Only whole, undamaged valves were considered. Those valves visible from the girdle view were counted as two (Picińska-Fałtynowicz & Błachuta, 2010). The taxonomic identification was mainly based on Bąk et al. (2012). In addition, extensive auxiliary literature was used, including Krammer and Lange-Bertalot (1986, 1988, 1991a, b), Krammer (2000, 2002, 2003), Lange-Bertalot (2001), Levkov (2009), Lange-Bertalot et al. (2011), Hofmann et al. (2013), Levkov et al. (2013), Levkov et al. (2016) and Cantonati et al. (2017).

### Data treatment for the purpose of calculating the Polish multimetric diatom indices IO (rivers) and IOJ (lakes)

The Polish indices have been developed following the requirements of the EU Water Framework Directive (European Council, 2000); all the relevant information and equations used in this study can be found in Picińska-Fałtynowicz and Błachuta (2010) and Zgrundo et al. (2018). The two Polish indices, which—in their structure—rely on modified Rott’s trophy and saproby indices (Rott, 1991; Rott & Pipp, 1999), assess the level of trophy (TI), saprobity (SI) and the contribution of reference species (GR; the degree of deviation of the diatom assemblage examined from the reference one) for rivers, and only SI and GR for lakes. The components are characterised by values with different numerical ranges; therefore, to calculate the final value, they are normalised. IO is the arithmetic mean of the three components (normalised TI, SI and GR), while IOJ is the arithmetic mean of the two components (normalised TI and GR). Generally, the diatom indices range from 0 (indicating hypertrophy and a low proportion of reference species in a sample) to 1 (ultra-oligotrophy, along with the assemblage resembling a reference one). The IO values were classified according to Picińska-Fałtynowicz and Błachuta (2010). The classification is given in Table 1. Moreover, during the original analyses, the assemblage biodiversity was determined with the natural logarithm-based Shannon–Wiener index (H’) (Krebs, 1972, 1989; Shannon, 1948; Shannon & Weaver, 1964).

**Table 1:** The classification of ecological status used in this study (Picińska-Fałtynowicz & Błachuta, 2010). *IO*, Polish diatom index for rivers; *IOJ*, Polish diatom index for lakes

IO		IOJ	
**Status**	**Index value**	**Status**	**Index value**
Very good	> 0.7	Very good	> 0.8
Good	0.5–0.7	Good	0.6–0.8
Moderate	0.3–0.5	Moderate	0.4–0.6
Poor	0.15–0.3	Poor	0.15–0.4
Bad	< 0.15	Bad	< 0.15

### Diatom valve identification (‘counting’): secondary analyses

Original analyses involved identification of a minimum of 400 diatom valves per sample (slide) and recording the number of valves representing each species (henceforth referred to as the primary counts). To optimise the counting process, we used the game approach. Each diatom valve from the primary analyses in every sample was given a unique natural number. Then, 50, 100, 150, 200, 250, 300, 350 and 400 random, unique natural numbers corresponding to individual valves were selected from each sample. The draws were made using the RAND function in an additional column of the Microsoft Office Excel spreadsheet containing the original data. The function returns a real number with a uniform distribution that is greater than 0 and less than 1 (e.g. 0.7732). The number of draws performed using the function corresponds to the number of all diatom valves identified in a sample. To obtain draws of unique natural numbers from the required range, the next column used the RANK.EQ formula. The function first retrieved items with the highest value in the auxiliary column and returned their position on the list of numbers relative to others. If more than one value had the same position, the function returned to the highest position in the entire set of values. After setting the highest value and returning it as a value of 1, the function performed the same action for the second, third, fourth, etc. values in terms of the size of the number from the auxiliary column. In this case, the function played a key role—each randomly selected value from the RAND function was assigned a unique value of the natural number corresponding to the number of the randomly selected diatom valve. It is worth remembering that the RAND function does not require any arguments and changes its value whenever a worksheet or formulas are altered in a workbook. To obtain a meaningful artificial dataset, the drawing procedure was repeated 20 times for each total valve count (i.e. 20 draws of up to 50 valves, 20 draws of up to 100).

This procedure resulted in dataset which consisted of 20 random species counts chosen from primary count in each counting range (160 counts for each sample). Entire dataset reached 8640 artificially generated diatom valve count sums for all 54 sites. For each generated count sum, adequate Polish diatom index (IO or IOJ) was calculated. Obtained matrix was used for further numerical and statistical treatment. The changes of IO and IOJ values were tested by applying the Kruskal–Wallis test for each sample (one-way ANOVA; Kruskal & Wallis, 1952). The result indicates whether differences between medians for each counting sum within one sample were significant. If obtained *p*-value for a sample is more than the significance level of 0.05, the medians are equal (no significant differences between obtained IO and IOJ results for each counting sum). Then, each artificially generated counting sum was compared to the primary study results by calculating goodness-of-fit of our simulation (percentage of values which gave similar ecological classification). Such goodness-of-fit results indicate how much of the artificially generated IO and IOJ values would have resulted in the same classification as during the primary studies (*n* = 1080 counts for each count sum). Moreover, the effect of reducing the counting diatom valves sum was shown by calculating the mean values and confidence intervals of standard deviation, also for each count sum in all samples. To both, goodness-of-fit and standard deviation plots, logarithmic trend line was generated, as the most suitable trend for obtained results. Additionally, samples were divided into categories of ecological status which was produced by the primary study counts and the box-and-whiskers plots were created, calculating median, values between lower and higher quartile (25–75%), as well as minimal and maximal values, for each artificially generated count sum (for each quantity of identified diatom valves).

## Results

### Original (primary) analyses

The primary analyses resulted in identifying 282 diatom taxa representing 56 genera in samples from the River Drawa system, and 278 taxa in 59 genera from the Ina system. The ecological status at the river’s source was very good (IO equal to 0.77 at site I1). It was the highest IO and the only site that was classified as along the Ina (Table 2). The IO values were gradually decreasing along the river course down to 0.51 at site I9. Thus, the River Ina section from kilometre 5 to 55 showed good ecological status. Downstream of that section, the Ina rolls through the town of Stargard (km 55–73) with sites I9–I13 showing the lowest IO values. IO dropped below 0.5, indicative of moderate ecological status. Further downstream (km 74–113; sites I14–I21), IO values rebounded to over 0.5 typical of good ecological status. In its mouth section (km 116–126; sites I22–I24), the ecological status declined to moderate. The last site (I24) showed the river’s lowest IO, below 0.4 (see Table 2). The Ina system lake sites (JK – Lake Krzemień, and JB – Lake Bytowo) showed good ecological status, the IOJ value being slightly higher at JB (Table 3).

**Table 2:** Values of the Polish diatom indices for river (IO), their components (TI, trophy level; SI, saprobity level; GR, reference species contribution) and the resultant ecological status (ES) assessment obtained at during the primary study

Site	TI	SI	GR	IO	ES
I1	1.58	1.73	0.96	0.77	Very good
I2	2.27	1.67	0.79	0.67	Good
I3	2.36	1.70	0.67	0.62	Good
I4	2.39	1.56	0.65	0.62	Good
I5a	2.55	1.62	0.59	0.58	Good
I5b	2.66	1.49	0.77	0.65	Good
I6	3.05	1.97	0.66	0.52	Good
I7	2.74	1.74	0.61	0.56	Good
I8	2.86	1.83	0.56	0.52	Good
I9a	2.83	1.96	0.57	0.51	Good
I9b	2.88	2.05	0.52	0.48	Moderate
I10	2.92	1.95	0.63	0.53	Good
I11	3.09	2.11	0.61	0.49	Moderate
I12	3.07	1.98	0.51	0.47	Moderate
I13	3.13	2.12	0.53	0.46	Moderate
I14	2.96	1.98	0.69	0.54	Good
I15	2.98	1.98	0.59	0.51	Good
I16	3.03	1.99	0.59	0.50	Good
I17	3.05	2.02	0.63	0.51	Good
I18	3.03	1.95	0.65	0.53	Good
I19	3.13	1.93	0.62	0.51	Good
I20	3.10	1.99	0.66	0.52	Good
I21	3.07	1.98	0.65	0.52	Good
I22	3.17	2.07	0.56	0.47	Moderate
I23	3.11	2.00	0.73	0.54	Good
I24	3.50	2.17	0.44	0.39	Moderate
D1	0.98	1.37	0.93	0.85	Very good
D2	2.57	1.71	0.83	0.65	Good
D3	2.24	1.60	0.83	0.71	Very good
D4	1.87	1.77	0.95	0.74	Very good
D5	2.69	1.94	0.82	0.61	Good
D6	3.22	1.94	0.53	0.47	Moderate
D7	2.48	1.72	0.73	0.62	Good
D8	2.92	1.81	0.65	0.55	Good
D9	2.72	1.75	0.68	0.58	Good
D10	2.70	1.87	0.62	0.55	Good
D11	2.77	1.55	0.67	0.60	Good
D12	2.77	1.68	0.62	0.57	Good
D13	2.97	1.72	0.51	0.51	Good

**Table 3:** Values of the Polish diatom indices for lakes (IOJ), their components (TI, trophy level; GR, reference species contribution) and the resultant ecological status (ES) assessment obtained at during the primary study

Sample	TI	GR	IO	ES
JK	3.04	0.48	0.61	Good
JB	2.74	0.68	0.71	Good
JD1	2.13	0.63	0.77	Good
JD2	3.15	0.21	0.50	Moderate
JD3	3.34	0.25	0.50	Moderate
JD4	3.08	0.32	0.54	Moderate
JD5	2.68	0.37	0.59	Moderate
JD6	3.36	0.03	0.41	Moderate
JD7	2.54	0.47	0.64	Good
JD8	2.45	0.60	0.72	Good
JD9	2.69	0.54	0.66	Good
JD10	3.60	0.32	0.51	Moderate
JD11	3.31	0.40	0.56	Moderate
JD12	2.45	0.47	0.64	Good
JD13	3.41	0.25	0.50	Moderate

The River Drawa average IO was 0.61 showing, like the Ina, a generally good condition. The highest IO were typical of the Drawa’s source section (km 6–16; sites D1–D5). At three sites, the IO exceeded 0.7, indicating very good ecological status (see Table 2). Most of the remaining sites along the entire course of the river displayed good ecological status, with IO oscillating between 0.51 (at the river mouth, km 180; site D13) and 0.62 (in the midstream, km 110; site D7). It was only at one site (km 90; site D6) that the IO dropped below 0.5 (the lowest value) indicative of moderate ecological status.

While the IOJ was at its highest (0.77) at the lake closest to the river source (Lake Krzywe; site JD1), indicating good ecological status, the remaining lakes of the Drawa’s upstream section (sites JD2–JD6) showed mostly moderate ecological status. The lowest IOJ (0.41; Lake Drawsko; site JD6) was most likely due to a sewage plant located near the sampling site. The next two upstream lakes (Lakes Krosino and Wilczkowo; site JD7–JD8) were characterised by good ecological status. Interestingly, the Wilczkowo sample produced IOJ of 0.72, the second highest IOJ across all the lakes studied (Table 3). The northern Lake Lubie site (JD9) showed a higher IOJ than the southern site (JD10), resulting in differing ecological status classification in a lake, good at JD9 and moderate at JD10. The Drawa’s midstream lakes (sites JD11–JD13) showed mostly moderate ecological status, Lake Grażyna (site JD12) being the only lake with good ecological status, although its IOJ (0.64) was close to the lower limit of the class. Other mid-stream lakes showed IOJ ranging from 0.49 to 0.56 (see Table 3).

### Secondary analyses: reduced valve counts

Our results showed that there were no significant differences between medians of each quantity of identified diatom valves for most of sites (Table 4), suggesting that there were no significant changes in majority of calculated IO and IOJ, regardless of the number of identified diatom valves. In only 6 of all 54 sites (0.111%), the differences between medians were statistically significant (italicised in Table 4). Such a small percentage of samples in which the reduction of the counted (identified) diatom valves had an impact on the obtained diatom index results allows us to conclude that lowering the number of identified diatom valves did not significantly affect the result of environmental assessment.

**Table 4:** Kruskal–Wallis test for equal medians results for each sampling site. If *p*-value is lower than significance level of 0.05, the medians between different count sums were not all equal and it can be concluded that the differences were statistically significant (italicised)

Rivers		
Site	*p*-value	Difference between medians
I1	0.097	Non-significant
I2	0.140	Non-significant
I3	0.620	Non-significant
*I4*	*8.85E-20*	*Significant*
I5a	0.211	Non-significant
I5b	0.356	Non-significant
I6	0.580	Non-significant
I7	0.357	Non-significant
I8	0.945	Non-significant
I9a	0.477	Non-significant
I9b	0.549	Non-significant
I10	0.630	Non-significant
I11	0.279	Non-significant
I12	0.989	Non-significant
I13	0.577	Non-significant
I14	0.130	Non-significant
I15	0.217	Non-significant
*I16*	*4.70E-11*	*Significant*
*I17*	*4.46E-06*	*Significant*
I18	0.496	Non-significant
I19	0.717	Non-significant
I20	0.407	Non-significant
*I21*	*1.10E-17*	*Significant*
I22	0.247	Non-significant
I23	0.984	Non-significant
I24	0.098	Non-significant
D1	0.917	Non-significant
D2	0.459	Non-significant
D3	0.611	Non-significant
D4	0.998	Non-significant
D5	0.146	Non-significant
D6	0.336	Non-significant
D7	0.853	Non-significant
D8	0.150	Non-significant
D9	0.754	Non-significant
D10	0.948	Non-significant
D11	0.230	Non-significant
D12	0.593	Non-significant
D13	0.276	Non-significant

Lakes		
Site	*p* value	Difference between medians
*JK*	*3.63E-15*	*Significant*
*JB*	*9.61E-15*	*Significant*
JD1	0.246	Non-significant
JD2	0.532	Non-significant
JD3	0.183	Non-significant
JD4	0.777	Non-significant
JD5	0.825	Non-significant
JD6	0.906	Non-significant
JD7	0.133	Non-significant
JD8	0.079	Non-significant
JD9	0.199	Non-significant
JD10	0.835	Non-significant
JD11	0.601	Non-significant
JD12	0.851	Non-significant
JD13	0.596	Non-significant

**Fig. 2:**
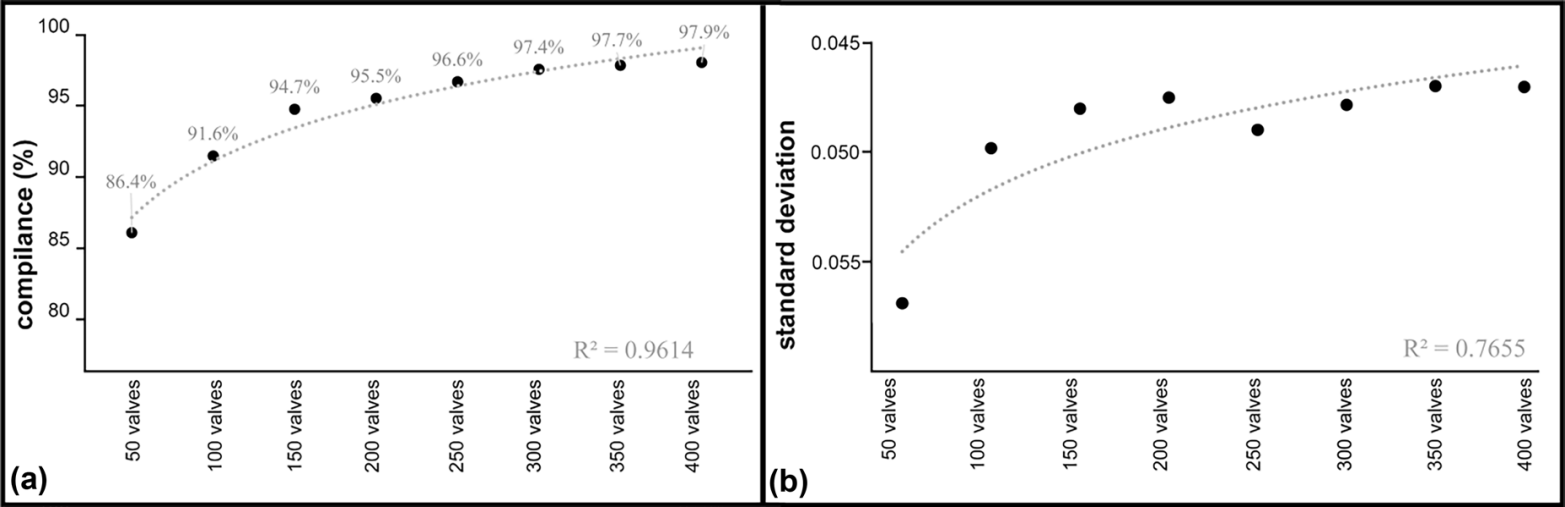
Goodness-of-fit of our simulation results with results obtained during primary studies (**a**) and the standard deviation of simulated polish multimetric diatom indices values (**b**) for each quantity of identified diatom valves (20 times for each of 54 samples; *n* = 1080) along with the best-fit trendline for obtained results (logarithmic)

Moreover, relation between the IO and IOJ values obtained from artificially generated counts and those from primary studies showed that more than 86% of draws of up to 50 valves resulted in ecological status assessment identical to that produced by the primary study (400 valves). When 100 valves were drawn, the goodness-of-fit of our simulation increased to more than 90%, and exceeded 95% for the number of 200 valves drawn (identified or ‘counted’; Fig. 2). In addition, when up to 50 valves were identified, the results showed a wider scatter, as expressed by standard deviation of 0.057, i.e. distinctly higher than that produced by identification (and counting) of 100 and more valves (standard deviation below 0.05 and systematically declining with increasing number of valves). It is worth noticing that, despite a visible declining standard deviation (increasing precision) with increasing number of identified (counted) valves, the largest drop in standard deviation was noticed between drawing 50 and 100 valves (standard deviation decreasing by 0.007), whereupon the differences remained more or less stable and standard deviation ranged from 0.050 to 0.047 (an 0.003 of scatter; Fig. 2b). In both, the goodness-of-fit and standard deviation, the best-fit trendline had a logarithmic trend. This proves that both rates of change in the data increase or decrease quickly and then level out. However, logarithmic trend was better representation for the changes of the goodness-of-fit data (Fig. 2a) than for the standard deviation (Fig. 2b). First trendline value of *R*-squared was 0.9614 suggesting very good fit, while the second value of *R*-squared was 0.7655. Nevertheless, both logarithmic trendlines, by their good fitness to the changes in data, showed that identifying (counting) more diatom valves increases the accuracy of environmental assessment in a logarithmic manner. It is worth noticing that the standard deviation plot is reversed: the more diatom valves were identified, the higher compliance with the original research we obtained, and opposite with the standard deviation, the more diatom valves were identified, the lower scatter the resulted IO and IOJ had.

**Fig. 3:**
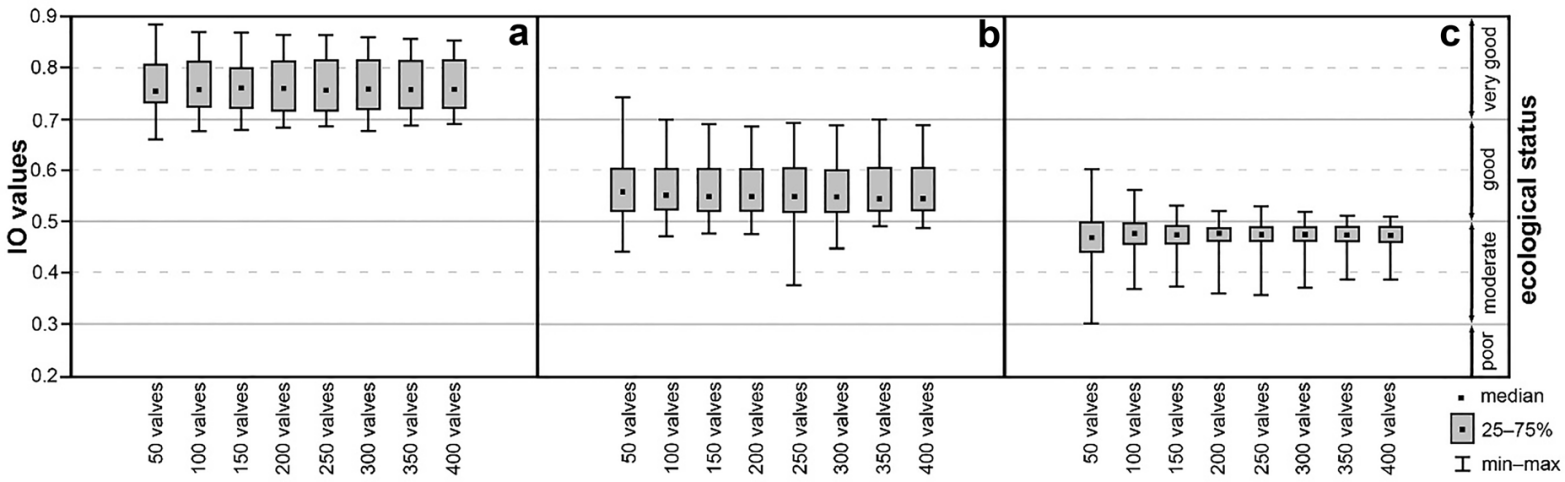
Box-and-whisker plots of polish multimetric diatom index for river (IO) values obtained during simulations for each amount of identified diatom valves. Samples have been grouped in accordance with the primary study results: (**a**) group 1, very good ecological status; (**b**) group 2, good ecological status; (**c**) group 3, moderate ecological status

**Fig. 4:**
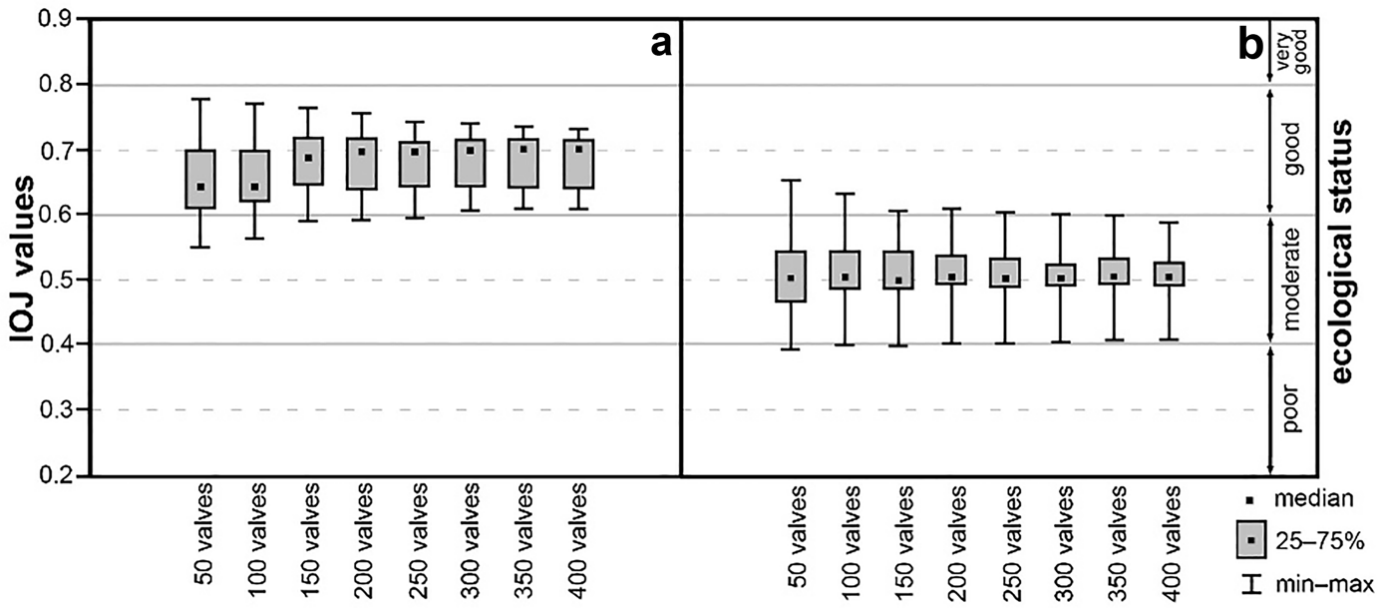
Box-and-whisker plots of polish multimetric diatom index for lakes (IOJ) values obtained during simulations for each amount of identified diatom valves. Samples have been grouped in accordance with the primary studies results: (**a**) group 2, good ecological status; (**b**) group 3, moderate ecological status. For IOJ, no sample has reached very good ecological status; therefore, no group 1 has been distinguished

For a better visualisation of changes in the Polish diatom indices for the results of our artificially generated counts, the samples were divided into three categories of ecological status as produced by the primary study (box-and-whiskers plots) for IO (Fig. 3) and IOJ (Fig. 4). The actual values of the indices are available in Supplementary material 3 and 4. The first group consisted of samples for sites classified by IO as very good, based on identification of more than 400 valves; these were 4 riverine sites (no lacustrine site was classified as very good in the primary study) with a total of 20 draws for each sample (each count sum – *n* = 80 draws; a in Fig. 3). The second and third group comprised sites classified as good and moderate, respectively. The second group (good ecological status) consisted of 28 sites for IO (each count sum – *n* = 560; b in Fig. 3) and 7 sites for IOJ (each count sum – *n* = 140; a in Fig. 4). The third group (moderate ecological status) was made up by 7 sites for IO (each count sum – *n* = 140; c in Fig. 3) and 8 sites for IOJ (each count sum – *n* = 160; b in Fig. 4). The interquartile distances for all the groups were within the range of IO and IOJ values typical of the respective ecological status categories, which were identical to those arrived at during the primary study. The interquartile ranges changed from 0.099 to 0.024, the range being clearly narrower for sites in the second and third group (cf. c in Fig. 3 and b in Fig. 4). While the interquartile distances for the first and second group (a and b in Fig. 3 and a in Fig. 4) changed from 0.99 to 0.7, the range for the third group was as low as 0.58–0.24 and 0.79–0.31 for IO and IOJ, respectively. Although the interquartile distances did not show significant change which could be associated with the number of valves identified, the ranges of the Polish diatom indices (minimum–maximum) decreased with increasing number of valves drawn (cf. whiskers in Figs. 3 and 4). The widest scatter of those values was typical of draws up to 50 valves, the scatter decreasing (from 0.31 to 0.12) with the increasing number of valves being drawn. The plot for the second group assessed with IO (b in Fig. 3) showed a wider IO scatter for draws of up to 250 valves. The maximum IO values were similar (about 0.69), but the minimal values dropped markedly to 0.37, while the remaining minimum values in the group ranged within 0.44–0.49 (Supplementary material 4; Fig. 3). It can be seen that most box-and-whisker plots are within the boundaries of an ecological status category arrived at during the primary study. The widest scatter of the IO and IOJ values obtained were observed for identification (‘counting’) of 50 and 100 valves. On the other hand, counting 150 and more valves resulted, in most cases, in small deviations from results of the primary study when more than 400 valves were counted.

## Discussion

Currently, many diatom researchers are involved in a discussion focusing on how many diatom valves, frustules or cells need to be identified (‘counted’) in a single permanent slide to get a statistically reliable autecological assessment. Often, the total number of valves or cells counted varies, depending on the purpose of the analysis (e.g. John & Birks, 2010; Karthick et al., 2010; Taylor et al., 2007). If the research is aimed at determining the true taxonomic structure of a diatom assemblage, the highest possible number of diatom cells or valves should be counted. Patrick et al. (1954) indicate a range of 3000–8000 cells as a representative data set. On the other hand, if the study is aimed at identifying only the diatom species dominant in an assemblage, it is recommended to count between 500 and 1000 cells per sample (Pappas & Stoermer, 1996). Schoeman (1973) ran an analysis in which 200, 300, 400, 500 and 800 valves per sample were counted and the relative abundances of the respective species were calculated. The results demonstrated that counting 200 valves produced 6–7% difference in the occurrence of individual species, compared to 800 counts. The difference dropped to 1–2% with 400 valves counted. Schoeman (1973) concluded that up to 400 valves per sample provided a satisfactory representation of the relative abundances of the species identified.

Subsequent studies (e.g. Battarbee, 1986; Prygiel et al., 2002) showed differences in percent contributions of individual species to be usually large when up to 100 and 200 valves were counted, and to decrease with up to 400 and 500 valves counted. It was therefore concluded that counting more than 300 valves per sample would not improve the result of diatom-based classification. In South Africa, it is recommended to count up to 400 diatom valves per slide (Taylor et al., 2007). In Poland, the Chief Inspectorate of Environmental Protection (ChIEP) recommends that a representative quantitative proportion of diatom taxa in an assemblage is obtained with up to 400 valves being counted. Actually, that number applies to the species on the IO and IOJ reference lists, which usually results in counts of well over 400 valves (Picińska-Fałtynowicz & Błachuta, 2010).

Although recommendations of researchers from different countries vary, most agree that counting up to 400 valves should be standard. However, as the actual count should be dependent on the purpose of the analysis, in the case of IO and IOJ (recommended by ChIEP), the numbers could be reduced without compromising the validity of the analysis. Our study demonstrated that this approach is a robust one. We demonstrated (cf. Figs. 3 and 4) that a reduction in the number of valves identified (‘counted’) in a single slide (sample) did not affect significantly the ecological status classification based on the Polish diatom indices for rivers (IO) and lakes (IOJ).

A similar conclusion was reported over 10 years ago by Bigler et al. (2010) who used diatom counts from 73 streams in northern Sweden and simulated results of reducing the number of valves counted for two indices: the IPS and the ACID. They randomly drew diatom valves to generate artificial assemblages of up to 40, 80, 120, 160, 200, 240, 280, 320, 360 and 400 diatom valves (‘counts’). The process was repeated 20 times for each sample (200 simulations per stream). For most of the simulated data, the IPS was not affected by the reduced count: the IPS remained the same regardless of counting 400 valves or counting up to 40 valves for 50 streams, and 80 valves for 60 streams (Bigler et al., 2010). Moreover, the data showed low standard deviations: for counts of up to 40 valves (a 90% reduction in the number of valves), standard deviation of the IPS was below 0.5 for 11 streams. On the other hand, the ACID data showed a reduction in the number of diatom valve count to affect the resultant classification: a classification resulting from counting 400 valves was repeated for as few as 12 streams with 40 counted diatom valves and 24 streams with a count of 80 (Bigler et al., 2010). This may suggest that different indices may respond differently to the reduction of the identification effort.

Our results showed that reducing the sum of identified diatom valves in a sample had no statistically significant changes between obtained IO and IOJ values (only 6 samples reached the *p*-value lower than statistical significance level as the result of nonparametric Kruskal–Wallis test for equal medians). The compliance between IO and IOJ values obtained by simulated counts and those obtained during the primary research had logarithmic trendline as the most fitted to the changes, indicating that the goodness-of-fit increases quickly when counted up to 50 and 100 valves per sample and stabilise above 95% of compliance when counting to more than 200 valves per sample. Despite that counting up to 50 valves carries an uncertainty (as expressed by standard deviation) similar to that when up to 400 valves were counted (0.057 vs 0.047, respectively; Fig. 2b), the trendline showed similar character of the changes with quick decrease in standard deviation when counting up to 50 and 100 valves per sample and stabilisation upwards. Therefore, the IO and IOJ may be expected to remain unaltered when the recommended count of up to 400 valves is halved to 200 valves per sample. The more valves are counted (i.e. identified), the more time-consuming a microscopic analysis becomes, and a reduction in the number of valves identified brings a considerable saving of time, whereby more samples can be analysed during the time allotted, which may then allow to adjust the sampling regime (e.g. increase the density of the sampling site network in a water body).

## Conclusion

The study showed that the overall pattern of river and lake ecological status, as determined using the Polish diatom-based indices IO and IOJ, is not affected significantly by halving the recommended number of diatom valves identified (‘counted’), while the reduction in the number of valves counted (from 400 to 200) will bring a considerable saving in time, thereby allowing to increase the sampling effort without compromising the outcome of the ecological status assessment. Our results clearly indicate that considerable simplification in methodology of Polish multimetric diatom indices, without compromising the ecological assessment result is possible. Using our recommendation, the same ecological assessment classification would occur with 95% of confidence to classification obtained when identifying (counting) to 400 and more diatom valves in a sample, when the counted valves’ sum was reduced to 200 valves per sample.

## Supplementary Information

Below is the link to the electronic supplementary material.

Supplementary file1 (PDF 117 KB)

Supplementary file2 (PDF 300 KB)

Supplementary file3 (PDF 231 KB)

Supplementary file4 (PDF 254 KB)

## Data Availability

All data generated and analysed during this study are provided online as supplementary information files (Supplementary material 1–4). The raw datasets are available from the corresponding author (AK) on reasonable request. Diatom microscopic slides used in this study are included in the Szczecin Diatomological Collection (SZCZ) of the Institute of Marine and Environmental Sciences at the University of Szczecin with the following numbers: Ina River system from 21,019 to 21,044 and Drawa River system from 21,048 to 21,074.
