# Supplementary material for: Is it possible to optimise the labour and time intensity of diatom analyses for determination of the Polish Diatom Indices (IO, IOJ)?
Source: Environ Monit Assess. 2022 Nov 3;195(1):64. doi: 10.1007/s10661-022-10676-7 (PMC9633445; doi:10.1007/s10661-022-10676-7)
Supplement: Supplementary file 1 — Supplementary file1 (PDF 117 KB) [file 10661_2022_10676_MOESM1_ESM.pdf]

Supplementary material 1

Supplementary table A Geographical coordinates of samples taken along the River Ina system. The description consists of the body of water to which the sample relates. The nearest town or additional information is given in brackets. Sampes are given in order from source to estuary.

RIVER INA SYSTEM			
**Sample**	**Geographical coordinates**		**Description**
I1	53° 24' 31.4" N	15° 36' 6.2" E	River Ina (Gronówko)
I2	53° 23' 50.4" N	15° 34' 7.5" E	River Ina (Ciemnik)
JK	53° 22' 14.7" N	15° 31' 57.6" E	Lake Krzemień
I3	53° 21' 49.2" N	15° 31' 35.1" E	River Ina (between Krzemień/Bytowo)
JB	53° 20' 27.4" N	15° 32' 37.3" E	Lake Bytowo
I4	53° 19' 52.6" N	15° 32' 50.3" E	River Ina (Sulibórz/Zdbino)
I5a	53° 17' 24.8" N	15° 32' 49" E	River Ina (Rybaki, above old mill)
I5b	53° 17' 22.3" N	15° 32' 48.9" E	River Ina (Rybaki, below old mill)
I6	53° 15' 25.4" N	15° 32' 6.3" E	River Ina (Recz Pomorski)
I7	53° 15' 13.7" N	15° 19' 54.9" E	River Ina (Suchań)
I8	53° 15' 56.5" N	15° 15' 2.5" E	River Ina (Lipka)
I9a	53° 16' 23.3" N	15° 11' 19.7"	River Ina (Rzeplino, above lock)
I9b	53° 16' 23.4" N	15° 11' 18.7" E	River Ina (Rzeplino, below lock)
I10	53° 17' 3.7" N	15° 7' 31.2" E	River Ina (Krępcewo/Radziszewo)
I11	53° 17' 32.7" N	15° 5' 2.6" E	River Ina (Witkowo Pierwsze)
I12	53° 18' 57.5" N	15° 3' 29.1" E	River Ina (Stargard south)
I13	53° 21' 36" N	15° 2' 11.2"’ E	River Ina (Stargard north)
I14	53° 21' 52.2" N	15° 1' 50.6" E	River Ina (Klępino)
I15	53° 22' 39.7" N	15° 0' 23.2" E	River Ina (Lubowo)
I16	53° 25' 18.9" N	14° 56' 53.5" E	River Ina (Poczernin)
I17	53° 26' 7.5" N	14° 54' 23.8" E	River Ina (Sowno)
I18	53° 29' 29.5" N	14° 51' 58.3" E	River Ina (Łęsko)
I19	53° 32' 57.9" N	14° 50' 29.3" E	River Ina (Goleniów south)
I20	53° 34' 2.2" N	14° 48' 35.9" E	River Ina (Goleniów north)
I21	53° 33' 55.4" N	14° 47' 34.6" E	River Ina (Domastryjewo)
I22	53° 33' 43.4" N	14° 45' 9.2" E	River Ina (Ininka)
I23	53° 33' 13.3" N	14° 42' 9.2" E	River Ina (kayak stop)
I24	53° 32' 3.6" N	14° 38' 11.3" E	River Ina (Inoujście)

Supplementary table B Geographical coordinates of samples taken along the River Drawa system. The description consists of the body of water to which the sample relates. The nearest town or additional information is given in brackets. Sampes are given in order from source to estuary.

RIVER DRAWA SYSTEM			
**Sample**	**Geographical coordinates**		**Description**
JD1	53°42'29.98" N	16°08'43.96" E	Lake Krzywe
JD2	53°42'03.38" N	16°09'21.21" E	Lake Długie
JD3	53°41'28.80" N	16°10'02.61" E	Lake Głębokie
D1	53°38'24.16" N	16°12'33.87" E	River Drawa (Prosinko)
D2	53°34'32.94" N	16°04'31.37" E	River Drawa (near old mill)
JD4	53°36'08.91" N	16°11'46.90" E	Lake Żerdno (Stare Drawsko)
JD5	53°36'09.20" N	16°11'31.26" E	Lake Drawsko (Stare Drawsko)
JD6	53°33'23.49" N	16°13'26.91" E	Lake Drawsko (sewage plant)
JD7	53°34'15.87" N	16°04'44.08" E	Lake Krosino (Głęboczek)
JD8	53°32'25.63" N	16°05'47.35" E	Lake Wilczkowo (Siemczyno)
D3	53°32'34.74" N	16°02'46.46" E	River Drawa (Budowo)
D4	53°32'21.08" N	15°49'37.82" E	River Drawa (Drawsko Pomorskie)
D5	53°29'45.11" N	15°48'35.66" E	River Drawa (Mielenko Drawskie)
JD9	53°27'15.06" N	15°56'35.10" E	Lake Lubie north (Lubieszewo)
JD10	53°29'25.73" N	15°51'22.26" E	Lake Lubie south (Gudowo)
JD11	53°23'38.11" N	15°51'22.26" E	Lake Wielkie Dębno
D6	53°18'17.77" N	15°46'42.67" E	River Drawa (Prostynia)
D7	53°16'14.30" N	15°46'24.22" E	River Drawa (Rościn)
JD12	53°13'08.46" N	15°45'49.70" E	Lake Grażyna
JD13	53°12'43.60" N	15°44'54.06" E	Lake Adamtowo
D8	53°10'39.74" N	15°47'55.35" E	River Drawa (Barnimie)
D9	53°08'26.85" N	15°50'55.32" E	River Drawa (Zatom)
D10	53°04'46.43" N	15°54'50.47" E	River Drawa (Moczele)
D11	53°00'46.97" N	15°57'53.36" E	River Drawa (Stare Osieczno)
D12	52°57'12.49" N	15°57'53.36" E	River Drawa (Hutniki)
D13	52°52'05.30" N	15°59'04.02" E	River Drawa (Bielice Nowe)
