# Supplementary material for: Is it possible to optimise the labour and time intensity of diatom analyses for determination of the Polish Diatom Indices (IO, IOJ)?
Source: Environ Monit Assess. 2022 Nov 3;195(1):64. doi: 10.1007/s10661-022-10676-7 (PMC9633445; doi:10.1007/s10661-022-10676-7)
Supplement: Supplementary file 2 — Supplementary file2 (PDF 300 KB) [file 10661_2022_10676_MOESM2_ESM.pdf]

Supplementary material 2

Supplementary Table C Results of primary studies of River Ina system. Shannon biodiversity index and species richness with a list of all idetified taxa encoded relative abundances (- = not abundant; + = <1%; 1 = 1–3%; 2 = 3–5%; 3 = 5–10%; 4 = 10–20% and 5 = >20%)

BIODIVERSITY INDICES	I1	I2	JK	I3	JB	I4	I5a	I5b	I6	I7	I8	I9a	I9b	I10	I11	I12	I13	I14	I15	I16	I17	I18	I19	I20	I21	I22	I23	I24
species richness	18	36	37	58	37	31	51	41	66	51	56	56	48	70	62	51	63	59	68	70	59	58	57	62	47	61	51	42
Shannon biodiversity index	0.79	3.62	3.92	4.90	3.46	2.82	3.89	4.12	5.05	4.75	4.60	4.82	4.92	5.43	5.13	4.53	5.03	4.55	4.93	5.28	4.89	4.93	5.02	5.02	4.46	5.14	4.76	3.59
RELATIVE ABUNDANCES																												
TAXA	I1	I2	JK	I3	JB	I4	I5a	I5b	I6	I7	I8	I9a	I9b	I10	I11	I12	I13	I14	I15	I16	I17	I18	I19	I20	I21	I22	I23	I24
Achnantidium caledonicum	-	+	-	-	-	-	-	-	-	-	-	-	-	-	-	-	-	-	-	-	-	-	-	-	-	-	-	-
Achnantidium minutissimum	5	2	2	2	2	+	2	1	+	-	+	1	1	2	2	1	1	1	+	+	+	+	+	1	+	1	1	2
Amphora copulata	-	-	+	-	+	-	-	-	-	+	+	+	1	1	-	2	1	1	2	1	1	-	1	1	1	-	1	2
Amphora eximia	-	-	-	-	-	-	-	-	-	-	-	+	-	-	+	-	+	-	-	-	-	-	-	-	-	-	-	-
Amphora inariensis	-	-	-	2	-	-	+	+	+	+	1	1	-	+	+	-	-	1	-	+	+	+	+	-	1	-	-	+
Amphora minutissima	-	-	-	+	-	-	-	-	-	-	-	-	-	-	-	-	-	-	-	-	-	-	-	-	-	-	-	-
Amphora ovalis	-	-	-	+	-	-	+	-	+	+	1	-	+	+	+	+	+	+	+	1	+	+	-	-	+	+	-	+
Amphora pediculus	-	-	+	1	+	-	-	-	-	-	+	+	-	-	+	1	-	+	1	+	+	-	-	-	-	+	-	2
Anomoeoneis sphaerophora	-	-	-	-	-	-	-	-	-	-	-	-	-	-	-	-	+	-	-	-	-	-	-	-	-	-	-	-
Asterionella formoza	-	-	-	-	-	1	1	1	1	-	-	-	-	-	-	-	-	-	-	-	-	-	-	-	+	-	-	-
Aulacoseira alpigena	-	-	-	-	-	-	-	-	-	2	-	-	-	-	-	-	-	-	-	-	-	-	-	-	-	-	-	-
Aulacoseira ambigua	-	-	-	+	-	+	2	2	1	2	2	2	-	2	+	1	+	+	1	1	1	1	-	+	1	-	+	-
Aulacoseira granulata	-	-	-	+	2	2	2	2	2	2	2	2	2	2	1	2	2	1	2	2	2	2	2	1	1	1	+	+
Aulacoseira italica	-	-	-	+	-	-	-	-	-	1	-	+	-	-	-	-	-	-	-	-	-	-	-	-	-	-	-	-
Aulacoseira subarctica	-	-	-	-	-	+	-	-	-	-	-	-	-	-	-	-	-	-	-	-	-	-	-	-	-	-	-	-
Caloneis amphisbaena	-	+	-	-	-	-	+	-	-	-	-	-	-	+	-	-	+	-	+	+	+	-	-	-	-	-	+	-
Caloneis lancettula	-	-	-	1	-	-	-	-	-	-	-	-	-	-	-	-	-	-	-	+	+	-	-	-	-	-	-	-
Caloneis silicula	-	-	-	-	-	-	-	-	-	-	-	-	-	-	-	-	-	-	-	-	+	-	+	-	-	-	-	-
Caloneis sp.	-	-	-	-	-	-	-	-	+	-	-	-	-	-	-	-	-	-	-	-	-	-	-	-	-	-	-	-
Cavinula lapidosa	-	-	-	-	-	-	-	-	-	-	-	-	-	+	-	-	-	-	-	-	-	-	-	-	-	-	-	-
Cocconeis disculus	-	-	-	1	-	-	+	-	-	-	-	+	-	-	-	-	-	-	-	-	-	-	-	-	-	-	-	-
Cocconeis neodiminuta	-	1	-	1	-	-	-	-	-	-	-	-	-	-	-	-	-	-	-	-	-	-	-	-	-	-	-	-
Cocconeis neothumensis	-	-	-	1	-	-	-	-	-	-	-	-	-	+	-	-	-	-	+	-	-	+	-	-	-	-	-	-
Cocconeis pediculus	-	-	-	+	-	-	-	-	-	-	-	-	-	-	-	-	-	-	-	-	-	-	-	-	-	-	-	-
Cocconeis placentula var. *euglypta*	-	1	-	2	-	+	1	-	-	1	-	1	-	1	1	-	1	1	-	-	1	1	1	+	-	+	-	-
Cocconeis placentula var. *lineata*	-	+	-	-	-	-	-	-	-	+	-	-	-	-	-	-	-	+	-	-	-	-	-	-	-	-	-	-
Cocconeis placentula var. *placentula*	-	1	+	2	+	-	1	1	1	1	1	2	1	2	2	2	2	2	2	2	2	2	2	2	2	2	2	2
Craticula accomoda	-	-	-	-	-	-	-	-	-	-	-	-	+	-	-	-	-	-	+	-	-	-	-	-	-	-	-	-
Craticula ambigua	-	-	-	-	-	-	-	-	+	-	-	-	-	-	-	-	-	-	+	-	-	-	-	-	-	-	-	-
Craticula cuspidata	-	-	-	-	-	-	-	-	+	-	-	+	-	-	-	-	-	-	-	-	-	-	-	-	-	-	-	-
Cyclostephanos dubius	-	-	-	1	+	2	2	2	-	2	2	-	2	-	1	2	2	1	2	2	2	2	2	1	2	2	1	+
Cyclostephanos invisitatus	-	+	-	-	-	+	-	1	+	1	1	-	-	+	-	-	-	-	-	+	-	-	-	-	-	+	-	-
Cyclotella atomus	-	+	-	+	+	+	+	1	1	2	2	2	2	1	+	1	-	+	1	+	+	1	+	-	1	1	-	-
Cyclotella balatonis	-	-	+	1	-	+	-	1	-	+	+	-	-	-	-	-	+	-	+	+	+	+	+	-	-	-	+	-
Cyclotella bodanica	-	-	-	-	-	-	+	-	-	-	-	-	-	-	-	-	-	-	-	-	-	-	-	-	-	-	-	-
Cyclotella glabriuscula	-	-	-	-	-	-	-	-	+	-	-	-	-	-	-	-	-	-	-	-	-	-	-	-	-	-	-	-
Cyclotella meneghiniana	-	1	-	-	-	-	-	1	1	2	2	2	2	2	1	2	1	2	2	2	2	2	1	1	2	2	+	-
Cyclotella ocellata	-	-	+	2	-	-	1	1	-	+	+	-	-	-	-	-	+	-	-	-	-	-	-	-	-	-	-	-
Cyclotella pseudostelligera	-	-	-	-	-	-	-	-	-	1	-	-	-	-	-	-	-	-	-	-	+	-	-	-	-	-	-	-
Cyclotella radiosa	-	-	-	-	-	+	1	-	-	1	+	+	-	-	-	-	-	-	-	-	-	-	-	-	-	-	-	-
Cymatopleura librile	-	-	-	-	-	-	-	-	-	-	-	-	-	-	-	-	+	+	-	-	-	-	-	-	-	-	-	-
Cymatopleura solea	-	+	-	-	-	1	+	-	+	-	-	+	-	+	+	-	-	-	+	+	-	-	+	+	-	-	-	-
Cymbella caespitosa	-	-	2	-	-	-	-	-	-	-	-	-	-	-	-	-	-	-	-	-	-	-	-	-	-	-	-	-
Cymbella cymbiformis	-	-	+	-	-	-	-	-	-	-	-	-	-	-	-	-	-	-	-	-	-	-	-	-	-	-	-	-
Cymbella excisa	-	-	-	+	-	-	-	-	-	-	-	-	+	-	-	-	-	-	-	-	-	-	-	-	-	-	-	-
Cymbella hustedtii	-	-	-	+	-	-	+	-	-	-	-	-	-	-	-	-	-	-	-	-	-	-	-	-	-	-	-	-
Cymbella lanceolata	-	-	-	-	-	+	-	-	-	-	-	-	-	-	-	-	-	-	-	-	-	-	-	-	-	-	-	-
Cymbella neocistula	-	-	-	-	-	-	+	-	-	-	-	-	-	-	+	-	-	-	-	-	-	-	-	-	-	-	-	-
Cymbella tumida	-	-	-	-	-	-	-	-	-	-	-	-	-	-	-	-	-	-	-	-	-	-	-	+	-	-	-	-
Cymbella affiniformis	-	-	-	-	-	-	-	-	-	-	-	-	-	-	-	-	-	-	-	-	-	-	-	-	+	-	-	-
Diatoma moniliformis	-	-	-	-	-	-	-	-	-	-	-	-	2	-	-	-	-	-	-	-	-	-	-	-	-	-	-	-
Diatoma tenuis	-	-	-	-	-	-	-	-	-	-	-	-	-	-	-	-	-	+	-	-	-	-	-	-	-	-	-	-
Diatoma vulgaris	-	-	-	1	-	-	-	-	-	-	-	+	-	+	-	-	-	-	-	+	-	-	-	-	+	-	-	-
Diploneis fontanella	-	-	-	+	-	-	-	-	-	-	-	-	-	-	-	-	-	-	-	-	-	-	-	+	+	-	-	-
Diploneis oculata	-	-	-	-	-	-	-	-	-	-	-	+	-	-	-	+	-	-	-	-	-	-	-	-	-	-	-	-
Diploneis petersenii	-	-	-	-	-	-	-	-	-	-	-	-	-	-	-	+	-	+	-	-	-	-	-	-	-	-	-	-
Encyonema caespitosum	-	-	-	-	1	-	+	-	-	-	-	-	-	-	-	-	-	-	-	-	-	-	-	-	-	-	-	-
Encyonema minutum	-	-	-	-	-	-	-	-	-	+	-	-	-	-	-	-	-	-	-	-	-	-	-	-	-	-	-	-
Encyonema silesiacum	-	+	+	+	-	+	-	+	-	-	-	+	2	+	2	+	1	1	+	+	1	+	+	2	+	+	1	+
Encyonema ventricosum	-	-	-	-	+	-	-	-	-	-	-	-	2	-	-	-	-	-	-	-	-	-	-	-	-	1	2	1
Eolimna subminuscula	-	-	-	-	-	-	-	-	-	-	-	-	+	-	-	-	-	-	-	-	-	-	-	-	-	-	-	-
Epithemia adnata	-	-	2	+	2	+	-	-	-	-	-	-	-	-	-	-	-	-	-	-	-	-	-	-	-	-	-	-
Epithemia frickei	-	-	1	+	1	-	-	-	-	-	-	-	-	-	-	-	-	-	-	-	-	-	-	-	-	-	-	-
Epithemia sorex	-	-	2	-	2	-	-	-	-	-	-	-	-	-	-	-	-	-	-	-	-	-	-	-	-	-	-	-
Epithemia turgida	-	-	-	-	-	+	-	-	-	-	-	-	-	-	-	-	-	-	-	-	-	-	-	-	-	-	-	-
Eunotia bilunaris	+	-	-	-	-	-	-	-	-	-	-	-	-	-	-	-	-	-	-	-	-	-	-	-	-	-	-	-
Eunotia implicata	-	-	-	+	-	-	-	-	-	-	-	-	-	-	-	-	-	-	-	-	-	-	-	-	-	-	-	-
Fragilaria bidens	-	-	-	+	-	-	-	-	-	-	-	-	-	-	-	-	-	-	-	1	-	-	-	-	-	-	-	-
Fragilaria capucina	-	1	2	-	-	-	-	-	1	-	-	-	-	-	-	-	+	-	1	-	-	-	+	-	-	-	-	-
Fragilaria delicatissima	-	+	-	-	-	-	-	-	-	-	-	-	-	+	-	-	-	-	-	-	+	-	-	-	-	-	-	-
Fragilaria dilatata	-	-	-	-	-	+	-	-	-	-	-	-	-	+	-	-	-	-	-	-	-	-	-	-	-	-	-	-
Fragilaria mesolepta	-	-	-	-	-	-	+	-	+	-	-	-	-	-	-	-	-	-	-	-	-	-	-	+	-	-	-	-
Fragilaria nanana	-	+	-	+	-	+	+	-	-	-	+	-	+	-	-	-	-	-	-	-	-	+	-	-	-	-	-	-
Fragilaria parasitica var. *constricta*	-	-	-	-	+	-	-	-	-	-	-	+	-	-	-	-	-	-	+	-	-	+	-	+	-	+	-	-
Fragilaria parasitica var. *parasitica*	-	-	-	-	-	+	-	-	-	-	-	-	-	-	-	-	-	+	-	-	-	-	-	-	+	-	-	-
Fragilaria perminuta	-	-	+	-	+	-	-	-	-	-	-	-	+	-	-	-	-	-	-	-	-	-	-	-	-	-	-	-
Fragilaria rumpens	+	1	-	+	+	-	-	-	-	-	-	-	+	-	-	-	-	-	-	+	-	-	-	-	-	-	-	-
Fragilaria tenera	+	-	-	-	+	-	-	-	-	+	-	-	-	-	-	-	-	-	-	-	-	-	-	-	-	-	-	-
Fragilaria vaucheriae	-	1	1	2	1	-	+	-	+	-	-	-	+	-	+	-	+	-	-	+	+	-	-	+	-	+	+	-
Fragilariforma bicapitata	-	2	-	-	-	-	-	-	-	-	-	-	-	-	-	-	-	-	-	-	-	-	-	-	-	-	-	-
Frustulia vulgaris	-	-	-	-	-	-	-	-	-	+	-	-	-	-	-	1	+	-	-	-	+	-	-	+	-	-	-	-
Geissleria acceptata	-	-	-	+	-	-	-	-	+	-	-	-	-	-	-	-	-	-	-	-	-	-	-	-	-	-	-	-
Geissleria decussis	-	-	-	-	-	-	-	-	-	-	+	1	-	-	-	+	+	-	+	+	-	+	-	+	+	-	+	-
Gomphonema acuminatum	-	-	-	-	+	-	-	-	-	-	-	-	-	-	-	-	-	-	-	-	-	-	-	-	-	-	-	-
Gomphonema angustatum	+	-	-	-	-	-	-	-	-	-	-	-	-	-	-	-	-	+	-	-	-	-	-	-	-	-	+	+
Gomphonema auritum	-	-	-	-	-	-	-	-	-	-	-	-	-	-	-	+	-	-	-	-	-	-	-	-	-	-	-	-

Gomphonema capitatum	+	-	-	-	-	-	-	-	-	-	-	-	-	-	-	-	-	-	-	-	-	-	-	-	-	-	-	-
Gomphonema clavatum	-	-	-	-	-	-	-	-	-	-	-	-	-	-	-	-	-	-	-	-	-	-	-	-	-	-	+	-
Gomphonema exilissimum	-	+	-	-	-	-	-	-	-	-	-	-	-	-	-	-	-	-	-	-	-	-	-	-	-	-	-	-
Gomphonema italicum	-	-	-	+	+	-	-	-	-	-	-	-	-	-	-	-	-	-	-	-	-	-	-	-	-	-	-	-
Gomphonema micropus	-	1	-	-	-	-	1	-	+	1	+	1	1	+	1	-	1	+	+	+	+	+	+	+	-	+	-	-
Gomphonema minutum	-	-	-	1	-	-	+	-	-	-	+	-	-	+	+	-	+	+	-	+	-	-	-	-	-	-	-	-
Gomphonema olivaceum	-	-	1	1	-	-	+	-	1	+	-	+	2	1	+	1	1	1	+	1	1	1	1	1	2	1	2	2
Gomphonema pala	-	-	-	+	-	-	-	-	-	-	-	-	-	-	-	-	-	-	-	-	-	-	-	-	-	-	-	-
Gomphonema parvulum	2	-	-	-	-	-	+	-	-	-	-	-	-	-	-	-	-	-	-	-	-	-	-	-	-	-	-	-
Gomphonema parvulum var. *parvulum* f. *parvulum*	-	1	1	-	1	-	-	-	1	1	+	1	1	1	2	-	2	2	1	+	1	1	1	2	1	2	2	1
Gomphonema parvulum var. *parvulum* f. *saprophilum*	-	-	+	-	-	-	+	-	+	+	-	-	-	1	1	-	1	+	+	1	+	+	-	+	+	+	-	+
Gomphonema procerum	-	+	-	-	-	-	-	-	-	-	-	-	-	-	-	-	-	-	-	-	-	-	-	-	-	-	-	-
Gomphonema productum	-	-	-	-	-	-	-	-	-	1	-	-	-	+	-	-	-	-	-	-	-	-	-	-	-	-	-	-
Gomphonema pumilum	-	-	2	+	1	-	-	-	-	-	-	-	-	-	+	-	-	-	-	-	+	-	+	+	-	-	1	1
Gomphonema sarcophagus	-	-	-	+	-	-	-	-	+	-	-	-	-	-	-	-	-	-	-	-	-	-	-	-	-	-	-	-
Gomphonema subclavatum	-	-	-	-	-	-	-	-	+	-	-	-	-	-	-	-	-	-	-	-	-	-	-	-	-	-	-	-
Gomphonema subtile	-	-	+	-	-	-	-	-	-	-	-	-	-	-	-	-	-	-	-	-	-	-	-	-	-	-	-	-
Gomphonema cymbelliclinum	-	-	-	-	-	-	-	-	-	-	-	-	-	-	-	-	-	+	-	-	-	-	-	-	-	-	-	-
Gomphonema parvulius	-	-	-	-	-	-	-	-	-	-	-	-	-	-	-	-	-	+	-	-	-	-	-	-	-	-	-	-
Gyrosigma acuminatum	-	-	-	-	-	-	-	+	+	-	+	-	-	+	-	-	-	-	-	+	-	-	+	+	-	+	-	+
Gyrosigma attenuatum	-	-	-	-	-	+	-	-	-	-	-	-	-	-	-	-	-	-	-	-	-	-	-	+	-	-	+	-
Gyrosigma sciotense	-	-	-	-	-	-	-	-	+	-	-	+	-	-	+	-	-	-	-	-	-	-	-	-	-	-	-	-
Halamphora montana	-	-	-	-	-	-	-	-	-	-	-	-	-	+	-	-	-	-	-	-	-	-	-	-	-	-	-	-
Halamphora veneta	-	-	-	-	-	-	-	-	-	-	-	-	-	-	+	-	-	-	-	-	-	-	-	-	-	-	-	-
Hantzschia abundans	-	-	-	-	-	-	-	+	-	-	-	-	-	-	-	-	-	-	-	-	-	-	-	-	-	-	-	-
Hippodonta capitata	-	-	-	+	-	-	-	-	+	1	1	+	+	+	1	+	+	1	1	2	1	1	1	1	1	1	+	+
Hippodonta hungarica	-	-	-	-	-	-	-	-	-	-	-	+	-	1	-	-	-	-	-	-	-	-	-	-	-	-	-	-
Hippodonta costulata	-	-	-	-	-	-	-	-	-	-	-	-	-	-	-	-	-	-	+	-	-	-	-	-	-	-	-	-
Hippodonta lueneburgensis	-	-	-	-	-	-	-	-	-	-	-	-	-	-	-	-	-	-	-	-	-	-	+	-	-	-	-	-
Karayevia clevei	-	-	-	1	-	-	-	-	-	-	+	-	-	-	-	-	-	-	+	-	-	-	-	-	-	-	+	+
Karayevia kolbei	-	-	-	+	-	-	-	-	-	-	-	-	-	-	-	-	-	-	+	-	+	-	-	-	-	-	-	-
Karayevia ploenensis	-	-	+	-	-	-	-	-	-	-	-	-	-	-	-	-	-	-	-	-	-	-	-	-	-	-	-	-
Karayevia suchlandtii	-	-	-	-	-	-	-	-	-	-	-	-	-	-	-	-	-	-	-	-	-	-	+	-	-	-	-	-
Kolbesia gessneri	-	-	-	+	-	-	-	-	-	-	-	-	-	-	-	-	-	-	-	-	-	-	-	-	-	-	-	+
Kolbesia kolbei	-	-	-	-	+	-	-	-	+	-	-	-	-	-	-	-	-	-	-	-	-	-	-	-	-	-	-	+
Lemnicola hungarica	-	-	-	-	-	-	+	+	-	-	-	-	-	-	-	-	-	+	-	+	+	+	-	-	-	-	-	-
Luticola goeppertiana	-	-	-	-	-	-	-	-	-	-	-	-	-	-	-	-	-	-	-	-	+	-	-	-	-	-	-	-
Luticola mutica	-	-	-	-	-	-	+	-	-	-	-	-	-	-	-	-	-	-	-	-	-	-	-	-	-	-	-	-
Luticola similis	-	-	-	-	-	-	-	-	-	-	-	+	-	-	-	-	-	-	-	-	-	-	-	-	-	-	-	-
Mayamaea atomus var. *atomus*	-	-	-	-	-	-	-	-	-	-	-	-	-	-	-	-	-	-	-	-	-	+	-	-	-	-	-	-
Melosira varians	-	-	+	-	1	+	+	-	-	-	-	-	2	1	1	-	1	1	1	1	+	1	-	-	-	1	1	+
Meridion circulare var. *circulare*	-	1	-	1	-	-	+	1	+	1	+	-	+	1	+	-	+	-	+	1	-	1	1	1	+	1	-	-
Navicula amphiceropsis	-	-	-	-	-	-	-	-	-	-	-	-	-	-	-	-	-	+	-	-	-	-	-	-	-	-	-	-
Navicula angusta	-	-	-	-	-	-	-	-	-	-	-	-	+	-	-	-	-	-	-	-	-	-	-	-	-	-	-	-
Navicula antonii	-	-	+	+	+	-	-	+	1	-	-	+	1	+	1	+	+	+	+	1	+	-	1	+	+	+	1	+
Navicula capitatoradiata	-	-	+	-	-	-	-	-	-	-	-	1	2	2	2	1	2	2	1	1	1	1	2	2	2	1	1	+
Navicula cari	-	-	-	-	-	-	-	-	2	-	-	-	-	-	+	-	+	+	-	-	-	-	+	-	-	-	-	-
Navicula cincta	-	-	-	-	-	-	-	-	-	-	+	-	-	-	-	-	-	-	-	-	-	-	-	-	+	-	-	-
Navicula cryptocephala	1	-	-	1	-	-	+	+	-	+	+	1	-	-	+	-	+	-	+	+	-	+	-	+	-	+	-	-
Navicula cryptofallax	-	-	-	-	-	-	-	-	+	-	-	-	-	-	+	-	+	-	-	-	-	-	-	-	-	-	-	+
Navicula cryptotenella	-	-	+	+	+	-	-	+	2	-	-	-	-	1	-	1	+	+	+	-	1	+	1	+	1	1	2	+
Navicula cryptotenelloides	-	-	-	+	-	-	-	-	-	-	-	-	-	-	-	-	-	-	-	-	-	-	-	-	-	-	-	-
Navicula diluviana	-	-	-	-	-	+	-	-	-	-	-	-	-	-	-	-	-	-	-	-	-	-	-	-	-	-	-	-
Navicula germainii	-	-	-	-	-	-	-	-	-	-	-	-	-	+	-	-	-	-	-	-	-	-	-	-	-	-	-	-
Navicula gregaria	-	+	-	-	-	-	-	-	2	-	+	1	2	2	2	2	2	2	+	1	1	1	2	2	1	2	2	+
Navicula integra	-	-	-	-	-	-	-	-	-	-	-	-	-	-	-	-	-	-	-	+	-	-	-	-	-	-	-	-
Navicula lanceolata	-	-	-	-	-	-	-	-	-	+	+	+	2	2	1	+	+	+	1	1	1	1	+	+	1	1	1	-
Navicula lundii	-	-	-	+	-	-	-	-	-	-	-	-	-	-	-	-	+	-	-	-	-	-	-	-	-	-	-	-
Navicula menisculus	-	-	-	-	-	-	-	-	-	-	-	-	-	-	-	+	-	-	-	-	-	-	-	-	-	-	-	-
Navicula moskalii	-	-	-	-	-	-	-	-	-	-	+	-	-	-	-	-	-	-	-	-	-	-	-	-	-	-	-	-
Navicula oblonga	-	-	-	-	-	-	-	-	-	-	+	-	-	-	-	-	-	-	+	-	-	-	+	-	-	+	+	-
Navicula oppugnata	-	-	-	-	-	-	+	-	-	-	+	1	-	1	-	1	+	+	1	1	+	+	1	+	+	-	-	-
Navicula radiosa	-	-	-	+	-	-	+	-	+	-	+	+	+	+	+	-	1	-	+	+	-	-	-	-	-	-	-	-
Navicula recens	-	-	-	+	-	-	-	-	1	-	-	-	-	-	+	-	-	-	-	-	-	-	-	-	-	-	-	-
Navicula reinchardtiana	-	+	+	+	+	-	-	-	-	+	+	+	1	-	2	+	1	1	+	+	-	+	+	1	+	+	+	+
Navicula reinhardtii	-	+	+	-	-	-	-	+	+	-	1	+	-	+	+	+	-	+	+	+	+	1	+	+	-	-	-	-
Navicula rhynchotella	-	-	-	-	-	-	-	+	-	-	-	-	-	-	-	-	-	-	-	-	-	+	-	-	-	-	-	-
Navicula slesvicensis	-	-	-	-	-	-	-	-	1	-	+	-	-	+	-	+	-	-	+	-	-	+	-	+	+	-	-	-
Navicula tenelloides	-	-	-	-	-	-	-	+	-	-	-	-	-	-	-	-	-	-	-	-	-	-	-	-	-	-	-	-
Navicula tripunctata	-	+	-	1	+	-	+	-	2	1	1	1	2	2	+	1	1	2	1	1	2	+	2	2	2	2	2	1
Navicula trivialis	-	-	-	-	+	-	-	+	1	-	+	1	-	1	1	+	1	1	1	1	1	+	+	-	1	+	+	-
Navicula umbonata	-	-	-	-	-	-	-	-	-	-	-	-	-	-	-	-	-	-	-	+	-	-	-	-	-	-	-	-
Navicula upsaliensis	-	-	-	-	-	-	+	-	-	-	+	+	-	-	+	-	+	+	1	+	-	-	+	-	+	+	-	-
Navicula veneta	-	-	-	-	-	-	-	-	-	+	-	-	-	+	+	+	+	-	+	-	+	+	-	-	-	-	-	-
Navicula viridula	-	-	-	-	-	-	-	-	-	-	-	-	-	-	+	-	+	-	+	+	-	-	-	-	-	-	-	-
Navicula vitabunda	-	-	-	-	-	-	-	-	+	-	-	-	-	-	-	-	-	-	-	-	-	-	-	-	-	-	-	-
Navicula wildii	-	-	-	-	-	-	-	-	-	+	-	-	-	-	-	-	-	-	-	-	-	-	-	-	-	-	-	-
Neidium affine	-	-	-	-	-	-	-	+	-	-	-	-	-	-	-	-	-	-	-	-	+	-	-	+	-	-	-	-
Neidium ampliatum	-	-	-	-	-	+	-	-	-	-	-	-	-	-	-	-	-	-	-	-	-	-	-	-	-	-	-	-
Neidium dubium	-	-	-	-	-	-	-	-	-	-	-	-	-	-	-	-	-	+	-	-	-	-	-	-	-	-	-	-
Nitzschia acicularis	-	-	-	-	-	-	-	-	-	-	-	-	-	-	-	-	-	-	-	+	-	-	-	-	+	+	-	-
Nitzschia adamata	-	-	-	-	-	-	-	-	-	-	-	-	-	-	-	-	-	-	-	-	+	-	-	-	-	+	-	-
Nitzschia agnita	-	-	-	-	-	-	+	-	-	-	-	1	1	+	-	+	-	+	+	+	+	-	-	-	-	-	-	-
Nitzschia amphibia	-	-	+	-	1	-	-	-	-	-	-	-	-	-	-	1	1	+	-	+	1	1	2	2	2	2	1	2
Nitzschia amphioxys	-	-	-	-	-	-	-	-	-	-	-	-	-	-	-	-	-	-	-	-	-	-	-	+	-	-	-	-
Nitzschia angustata	-	-	-	-	-	-	-	-	-	-	-	-	-	-	-	-	-	-	-	-	-	-	1	+	+	-	+	-
Nitzschia angustiforaminata	-	+	-	-	-	-	-	-	-	-	-	-	-	-	-	-	-	-	-	-	-	-	-	-	-	-	-	-
Nitzschia brunoi	-	-	-	-	-	-	-	-	+	-	-	-	-	-	+	-	-	-	-	-	-	-	+	-	-	+	-	-
Nitzschia calidia	-	-	-	-	-	-	-	-	-	-	-	-	-	-	-	-	-	-	-	-	-	+	-	-	-	-	-	-
Nitzschia capitellata	-	-	-	-	-	-	-	-	+	-	-	+	1	-	+	-	+	+	+	-	+	-	+	-	-	+	+	-
Nitzschia commutata	-	-	-	-	-	-	-	-	-	-	-	-	-	-	-	-	-	-	-	-	+	-	-	-	-	-	-	-
Nitzschia dissipata	+	-	+	-	-	-	-	+	1	-	-	-	+	1	1	+	+	+	+	-	+	+	-	+	+	+	1	+
Nitzschia dubia	-	-	-	-	-	-	-	-	1	-	-	-	-	-	-	-	-	-	-	-	-	-	-	-	-	-	-	-
Nitzschia fonticola	-	-	1	+	-	-	-	-	-	-	-	+	1	+	+	+	1	+	-	-	-	-	-	1	-	1	2	1
Nitzschia frustulum	-	-	-	-	-	-	-	-	-	-	-	-	-	-	-	-	-	-	-	-	+	-	-	-	-	-	-	-
Nitzschia gracilis	-	-	-	-	-	-	-	-	-	-	+	+	-	-	+	+	-	-	-	-	-	-	-	-	-	-	-	-
Nitzschia heufleriana	-	-	-	-	-	-	-	-	+	-	+	-	-	+	-	-	+	+	-	-	+	+	+	+	-	+	1	-
Nitzschia hungarica	-	-	-	-	-	-	-	-	-	-	-	-	-	+	+	-	+	+	-	+	-	-	-	-	-	-	-	-

Nitzschia intermedia	-	-	-	-	-	-	-	-	+	-	-	-	-	-	-	-	-	-	-	-	-	-	-	-	-	-	-	-
Nitzschia linearis	-	+	-	+	-	+	+	+	2	+	+	-	-	+	1	1	-	+	+	1	+	1	+	1	1	1	1	-
Nitzschia palea	+	-	+	-	-	-	-	-	+	-	-	-	+	+	1	-	1	1	-	-	-	+	+	+	-	+	+	+
Nitzschia paleacea	+	-	-	-	-	-	-	-	-	-	-	-	+	-	-	-	-	-	-	-	-	-	-	+	-	+	-	-
Nitzschia perminuta	+	-	-	-	-	-	-	-	+	-	-	-	-	-	-	-	-	-	-	-	-	-	+	-	-	-	1	+
Nitzschia pumila	-	-	-	-	-	-	-	-	-	-	-	+	-	-	-	+	-	-	-	-	-	-	-	-	-	-	-	-
Nitzschia pura	-	-	-	+	-	-	-	-	-	-	-	-	+	-	-	-	-	-	+	+	-	-	-	-	-	-	-	-
Nitzschia radicula	+	-	-	-	-	-	-	-	-	-	-	-	-	-	-	-	-	-	-	-	-	-	-	-	-	-	-	-
Nitzschia recta	-	-	-	+	-	-	+	-	2	-	-	+	-	1	1	1	1	1	1	+	+	1	1	1	1	2	1	+
Nitzschia regula	-	-	-	-	-	-	-	-	-	-	-	-	-	-	+	-	-	-	+	+	-	+	+	-	-	+	1	+
Nitzschia sigmoidea	-	-	-	-	-	-	-	-	1	-	-	-	-	-	+	-	+	-	+	-	-	+	+	+	-	-	-	-
Nitzschia sociabilis	+	-	-	-	-	-	-	-	+	-	-	-	-	-	-	-	-	-	-	-	-	-	-	-	-	-	-	-
Nitzschia sp. cf. *tenuis*	-	-	-	-	-	-	-	-	+	-	-	-	-	-	-	-	-	-	-	-	-	-	-	-	-	-	-	-
Nitzschia subacicularis	-	-	-	-	-	-	-	-	-	-	+	-	-	-	-	-	-	-	-	-	-	-	-	-	-	-	-	-
Nitzschia sublinearis	-	-	-	-	-	-	-	-	-	-	-	-	-	-	-	-	-	-	-	-	-	-	+	-	-	+	-	-
Nitzschia supralitorea	-	-	-	-	-	-	-	-	-	-	-	-	+	-	-	-	-	-	-	-	-	-	-	-	-	+	-	-
Nitzschia tubicola	-	-	-	-	-	-	-	-	-	-	-	-	-	-	-	-	-	-	+	-	-	-	-	-	-	-	-	-
Nitzschia vermicularis	-	-	-	-	-	-	-	-	+	-	-	-	-	-	-	-	-	-	-	-	-	-	-	-	-	-	-	-
Nitzschia wuellerstorffii	-	-	-	-	-	-	-	-	-	-	-	+	-	-	+	-	-	+	+	-	-	-	1	+	-	-	+	-
Nitzschia bulnheimiana	-	-	-	+	-	-	+	-	-	-	-	-	-	-	-	-	-	-	-	-	-	-	-	-	-	-	-	-
Pinnularia brebissonii	-	-	-	-	-	-	-	-	-	+	-	-	-	-	-	-	-	-	-	-	-	-	-	-	-	-	-	-
Placoneis clemensis	-	-	-	-	-	-	-	-	+	-	-	-	-	-	-	-	-	-	-	-	+	-	-	-	-	-	-	-
Placoneis elginensis	-	-	-	+	-	-	-	-	-	+	-	-	-	-	-	-	-	-	-	+	-	-	-	-	-	-	-	-
Placoneis gastrum	-	-	-	-	-	-	-	-	-	-	-	-	-	-	-	-	-	-	+	-	-	-	-	-	-	-	-	-
Placoneis paraelginensis	-	-	-	-	-	-	-	-	-	-	-	-	-	+	-	-	-	-	-	-	-	-	-	-	-	-	-	-
Placoneis placentula	-	-	-	-	+	+	-	-	-	-	-	-	-	-	-	-	-	-	-	+	-	-	+	-	-	-	-	-
Planothidium dubium	-	1	-	-	-	-	+	-	-	-	+	-	-	-	-	-	-	-	+	+	-	-	-	+	-	+	-	1
Planothidium frequentissimum	-	+	-	+	-	+	-	-	-	-	-	-	-	-	-	+	1	+	-	+	+	-	-	-	+	-	+	1
Planothidium granum	-	-	-	+	-	-	-	-	-	-	-	-	-	-	-	-	-	-	-	-	-	-	-	-	-	-	-	-
Planothidium lanceolatum	+	2	-	1	-	-	+	1	+	1	-	+	+	+	1	+	1	-	+	-	1	1	+	1	1	-	+	1
Planothidium minutissimum	-	-	-	-	-	-	-	-	-	2	-	-	-	-	-	-	+	-	-	+	-	-	-	-	-	-	-	-
Planothidium peragalli	-	+	-	-	-	-	-	-	-	-	-	-	-	-	-	-	-	-	-	-	-	-	-	-	-	-	-	-
Planothidium rostratum	-	-	-	+	-	-	-	-	-	-	-	1	-	2	+	-	-	-	-	-	-	+	-	+	+	+	-	+
Prestauroneis integra	-	-	-	-	-	-	-	-	-	-	-	-	-	-	-	-	-	-	-	-	+	-	-	-	-	-	-	-
Psammothidium bioretii	-	-	-	-	-	-	-	-	-	+	-	-	-	-	-	-	-	-	-	-	-	-	-	-	-	-	-	-
Psammothidium subatomoides	-	+	-	+	-	-	-	-	-	+	-	-	-	-	-	-	-	-	+	-	-	-	-	-	-	-	-	-
Psammothidium lauenburgianum	-	-	-	-	-	+	-	-	-	-	-	-	-	-	-	-	-	-	-	-	-	-	-	-	-	-	-	-
Pseudostaurosira binodis	-	-	-	-	1	2	1	2	-	+	-	-	-	-	-	-	-	-	-	-	-	+	+	-	-	-	-	-
Pseudostaurosira brevistriata	-	-	1	+	1	-	-	2	-	1	+	-	-	1	-	+	-	+	+	1	-	-	2	+	-	1	-	-
Reimeria sinuata	-	-	-	+	-	-	-	+	+	-	-	-	+	-	-	-	-	-	-	-	-	-	-	-	-	-	-	-
Reimeria uniseriata	-	-	-	-	-	-	-	-	-	-	+	-	-	-	-	-	-	-	-	-	-	-	-	-	-	-	-	-
Rhoicosphenia abbreviata	-	-	2	-	-	-	-	+	-	-	-	-	-	-	-	+	+	+	1	-	1	1	2	1	1	1	1	1
Rhopalodia gibba	-	-	1	-	-	-	-	-	-	-	-	-	-	-	-	-	-	-	-	-	-	-	-	-	-	-	-	-
Sellaphora bacillum	-	-	-	+	-	-	-	+	-	-	-	-	-	-	-	-	-	-	-	-	-	-	-	-	-	-	-	-
Sellaphora laevissima	-	-	-	-	-	-	+	-	-	-	-	-	-	-	-	-	-	-	-	-	-	-	-	-	-	-	-	-
Sellaphora pseudopupula	-	1	-	-	-	-	-	-	-	-	-	-	-	-	-	-	+	-	+	-	-	-	-	-	-	-	-	-
Sellaphora pupula	-	-	-	-	-	-	-	-	-	-	-	-	+	+	-	1	+	-	1	-	-	1	1	-	+	+	-	+
Stauroneis anceps	-	-	-	-	-	-	-	-	-	-	+	-	-	-	+	-	-	-	-	-	-	-	-	-	-	-	-	-
Stauroneis gracilis	-	-	-	-	-	-	-	-	+	-	-	-	-	-	-	-	-	-	-	-	-	-	-	-	-	-	-	-
Stauroneis kriegeri	-	+	-	-	-	-	-	-	-	-	+	-	-	-	-	-	-	-	-	-	-	-	-	-	-	-	-	-
Staurosira binodis	-	+	-	-	+	-	-	-	+	-	-	-	-	-	-	-	-	-	-	-	-	-	-	-	-	-	-	-
Staurosira construens	-	-	1	-	+	2	2	2	-	+	1	+	-	-	-	-	-	-	+	1	-	1	-	-	-	-	-	-
Staurosira lapponica	-	-	-	-	-	-	-	-	-	-	-	-	-	+	-	-	-	-	-	-	-	-	-	-	-	-	-	-
Staurosira venter	-	2	1	1	1	2	1	2	1	2	2	2	1	1	+	1	+	+	1	+	-	1	+	1	+	+	1	-
Staurosirella martyi	-	-	-	+	-	-	-	-	-	-	-	-	-	-	-	-	-	-	-	-	-	-	-	-	-	-	-	-
Staurosirella pinnata	-	2	-	1	-	+	1	1	+	1	+	2	-	1	1	-	+	1	1	1	+	2	-	1	-	1	-	-
Stephanodiscus alpinus	-	-	-	-	-	+	+	-	-	-	-	-	-	-	-	-	-	-	-	-	-	-	-	-	-	-	-	-
Stephanodiscus hantzschii	-	-	-	-	+	1	1	1	2	-	2	2	2	2	1	2	2	2	2	2	2	2	2	2	2	2	+	+
Stephanodiscus minutulus	-	-	-	+	-	-	-	-	-	-	-	-	-	-	-	-	-	-	-	-	-	-	-	-	-	-	-	-
Stephanodiscus neoastraea	-	-	-	1	-	1	+	-	-	1	2	1	1	+	-	-	-	-	1	1	1	-	-	+	-	-	-	-
Stephanodiscus parvus	-	-	-	-	-	-	-	-	-	2	2	1	-	-	-	-	-	-	-	-	-	-	-	-	-	-	-	-
Stephanodiscus sp.	-	-	-	-	-	-	-	-	-	1	-	-	-	-	-	-	-	-	-	-	-	-	-	-	-	-	-	-
Surilella minuta	-	-	-	-	-	-	-	-	+	-	-	-	-	-	-	-	-	-	-	-	-	-	-	-	-	-	-	-
Surirella angusta	-	-	-	-	-	-	-	-	+	-	-	-	-	-	-	-	-	-	-	+	-	-	-	-	-	-	-	-
Surirella bifrons	-	-	-	-	-	1	-	-	-	-	-	-	-	-	-	-	-	-	-	-	-	-	-	-	-	-	-	-
Surirella brebissonii var. *kuetzingii*	-	-	-	-	-	-	-	-	+	-	-	-	-	-	-	-	-	-	-	-	-	-	-	-	-	-	-	-
Surirella visurgis	-	-	-	-	-	-	-	-	-	-	-	-	-	+	-	-	-	-	-	-	-	-	-	-	-	+	+	-
Tabellaria fenestrata	-	-	-	1	+	-	-	-	-	-	-	-	-	-	-	-	-	-	-	-	-	-	-	-	-	-	-	-
Tabellaria flocculosa	-	-	-	-	-	-	-	+	-	-	-	-	-	-	-	-	-	-	-	-	-	-	-	-	-	-	-	-
Tabularia fasciculata	-	-	-	-	-	-	-	-	-	-	-	+	-	-	-	-	-	-	-	-	+	-	-	-	-	-	-	-
Thalassiosira proschkinae	+	-	-	-	-	-	-	-	-	-	-	-	-	-	-	-	-	-	-	-	-	-	-	-	-	-	-	-
Thalassiosira visurgis	-	-	-	-	-	+	-	-	-	-	-	-	-	-	-	-	-	-	-	-	-	-	-	-	-	-	-	-
Ulnaria acus	-	-	-	-	-	-	2	-	+	1	+	2	1	+	+	+	+	+	+	+	+	+	+	-	-	+	+	+
Ulnaria delicatissima var. *angustissima*	-	-	+	+	1	+	+	-	1	+	+	+	-	-	-	+	+	+	+	-	-	-	-	-	-	+	+	-
Ulnaria ulna	-	+	+	+	2	-	+	1	+	-	+	1	1	1	+	+	2	1	1	-	2	1	1	-	+	+	2	1

Supplementary Table D Results of primary studies of River Drawa system. Shannon biodiversity index and species richness with a list of all idetified taxa encoded relative abundances (- = not abundant; + = <1%; 1 = 1–3%; 2 = 3–5%; 3 = 5–10%; 4 = 10–20% and 5 = >20%)

BIODIVERSITY INDICES	D1	D2	JD1	JD2	JD3	JD4	JD5	JD6	JD7	JD8	D3	D4	D5	JD9	JD10	JD11	D6	D7	JD12	JD13	D8	D9	D10	D11	D12	D13
species richness	33	61	39	47	67	48	40	22	52	41	61	30	63	61	57	47	59	74	53	64	72	89	76	58	72	61
Shannon biodiversity index	1.41	3.11	2.74	2.85	3.39	3.06	2.83	1.51	2.93	2.90	3.09	1.99	3.34	2.98	3.13	2.67	3.09	3.77	1.44	3.52	3.65	3.84	3.50	3.20	3.34	3.17
RELATIVE ABUNDANCES																										
	D1	D2	JD1	JD2	JD3	JD4	JD5	JD6	JD7	JD8	D3	D4	D5	JD9	JD10	JD11	D6	D7	JD12	JD13	D8	D9	D10	D11	D12	D13
Achnanthes exigua	-	+	-	-	-	-	-	-	+	-	+	-	-	-	-	-	-	-	-	-	-	-	-	-	-	-
Achnanthes lutheri	-	-	-	-	-	-	-	-	-	-	-	-	-	-	-	-	-	-	-	+	-	-	-	-	-	-
Achnanthes oblongella	-	-	-	-	-	-	-	-	-	-	-	-	-	-	-	-	-	-	-	-	-	+	-	-	-	-
Achnanthes ziegleri	-	-	-	-	-	-	-	-	-	-	-	-	-	-	-	-	-	-	-	-	-	-	+	-	-	-
Achnanthidium affine	-	-	-	-	-	-	-	-	-	-	-	-	-	-	-	-	-	-	2	-	-	+	-	+	-	+
Achnanthidium eutrophilum	-	-	-	-	-	-	-	-	-	-	-	-	-	-	+	-	-	-	-	-	-	-	-	-	-	-
Achnanthidium gracillimum	-	-	-	-	-	-	+	-	-	2	-	+	-	-	-	-	-	-	+	-	-	-	-	-	-	-
Achnanthidium linearioides	-	-	-	-	-	-	-	-	-	-	-	-	-	-	-	-	-	-	-	+	-	-	-	-	-	-
Achnanthidium minutissimum f. *inconspicuum*	-	-	-	-	-	-	-	-	-	-	+	+	-	-	-	-	-	2	2	1	+	-	+	+	+	-
Achnanthidium minutissimum var. *jackii*	-	-	2	1	-	-	-	-	+	-	-	2	+	2	2	-	2	2	2	2	-	-	-	1	+	-
Achnanthidium minutissimum var. *minutissimum*	-	2	2	2	2	1	1	-	1	2	1	5	2	2	2	+	2	2	2	-	+	+	1	-	1	+
Achnanthidium pyrenaicum	-	-	-	-	-	-	-	-	-	-	-	-	-	-	-	-	-	-	+	+	1	-	+	-	-	-
Amphipleura pellucida	-	+	-	-	-	-	-	-	-	-	-	-	-	-	-	-	-	-	-	-	-	-	-	-	-	-
Amphora copulata	-	+	+	-	1	+	-	-	1	+	-	-	+	1	1	-	-	1	-	-	1	1	+	+	+	1
Amphora exima	-	-	-	-	-	-	-	-	-	-	-	-	-	-	-	-	-	-	-	-	-	-	+	-	-	-
Amphora inariensis	-	-	-	+	1	+	+	+	-	+	1	-	+	+	-	1	1	1	2	2	2	1	1	1	1	2
Amphora indistincta	-	1	-	-	-	-	1	-	1	-	-	-	-	1	+	-	+	-	-	2	1	1	-	1	-	-
Amphora lange-bertalotii	-	-	-	-	-	-	-	-	-	-	-	-	-	-	-	-	-	-	-	-	-	-	+	-	-	-
Amphora ovalis	-	-	-	-	-	-	-	-	-	-	-	-	-	-	-	-	-	-	-	-	-	-	-	-	+	+
Amphora ovalis var. *tenuis*	-	-	-	-	-	-	-	-	-	-	-	-	-	+	-	-	-	-	-	-	-	-	-	-	-	-
Amphora pediculus	+	1	-	-	1	2	2	+	2	2	1	+	1	2	+	1	2	1	2	1	2	1	1	-	1	2
Asterionella formosa	-	2	2	1	-	+	-	1	+	+	1	-	-	-	-	-	-	+	-	-	1	1	+	+	+	-
Aulacoseira ambigua	1	1	1	2	+	-	+	1	1	+	+	-	-	-	-	2	-	+	+	-	1	2	1	+	1	+
Aulacoseira granulata	-	2	2	2	-	-	-	-	-	-	-	-	-	-	-	2	-	-	-	-	1	1	+	+	+	+
Aulacoseira subarctica	-	-	-	2	-	-	-	-	-	-	-	-	-	-	-	+	-	-	-	-	-	-	-	-	-	-
Caloneis aerophila	+	-	-	-	-	-	-	-	-	-	-	-	-	-	-	-	+	-	+	-	-	-	-	-	-	-
Caloneis fontinalis	-	-	-	-	-	-	-	-	-	-	-	-	-	-	-	-	-	-	+	-	-	-	-	-	-	-
Caloneis lancettula	-	+	-	-	+	-	-	-	+	-	-	-	-	-	-	-	-	-	-	-	-	-	-	-	-	-
Caloneis molaris	-	-	-	-	-	-	-	-	-	-	-	-	-	-	-	-	-	-	-	-	-	-	-	-	-	+
Cavinula scutelloides	-	-	-	-	-	-	-	-	-	-	-	-	-	-	-	-	-	-	-	-	-	+	-	-	-	-
Cocconeis disculus	-	-	-	-	-	-	-	-	-	-	-	-	-	-	-	-	-	-	-	+	-	-	-	-	+	-
Cocconeis neodiminuta	-	1	-	+	1	+	-	2	1	+	1	-	+	-	+	-	-	1	+	1	+	-	+	+	-	-
Cocconeis neothumensis	+	+	-	-	+	1	1	1	1	1	-	-	-	-	-	-	+	+	-	-	-	-	+	-	-	-
Cocconeis pediculus	-	+	-	+	+	+	-	-	-	+	1	-	+	-	+	-	+	+	+	1	+	-	-	-	-	-
Cocconeis placentula var. *euglypta*	+	2	2	2	2	2	2	5	2	2	2	1	2	1	1	-	1	2	2	2	+	1	2	2	2	2
Cocconeis placentula var. *klinoraphis*	-	-	-	+	-	-	-	+	-	-	+	-	+	+	-	-	-	-	+	-	-	-	-	-	-	-
Cocconeis placentula var. *lineata*	-	+	-	+	+	-	-	1	-	+	+	-	+	+	+	-	-	1	1	1	1	+	1	1	+	-
Cocconeis placentula var. *placentula*	-	1	1	2	+	1	1	2	+	1	2	-	+	-	1	+	+	2	-	2	+	2	+	2	+	1
Cocconeis pseudolineata	-	-	-	-	-	-	+	1	-	-	+	-	2	+	-	-	-	1	-	1	+	+	+	+	-	-
Cocconeis pseudothumensis	-	-	+	-	-	-	-	-	-	-	-	-	-	-	-	-	-	-	-	-	-	-	-	-	-	-
Craticula buderi	-	-	-	-	-	+	-	-	-	-	-	-	-	-	-	-	-	-	-	-	-	-	-	-	-	-
Cyclostephanos dubius	-	2	-	-	+	-	-	+	+	2	1	-	-	+	1	2	+	1	+	+	2	2	2	2	2	2
Cyclostephanos invisitatus	-	-	-	-	-	-	-	-	-	-	-	-	-	-	-	2	-	+	-	-	-	-	+	+	+	+
Cyclotella atomus	-	-	+	+	-	-	-	-	-	-	-	-	-	-	-	1	-	-	-	-	+	1	+	-	+	+
Cyclotella balatonis	-	+	1	2	-	-	-	-	-	+	-	-	-	-	-	+	-	-	-	-	-	-	-	-	+	-
Cyclotella bodanica	-	-	-	-	-	-	-	-	-	-	-	-	-	-	-	+	-	-	-	-	-	-	-	-	-	-
Cyclotella meneghiniana	-	-	-	-	+	-	-	-	+	+	-	-	+	-	-	1	-	+	+	-	+	1	+	-	+	-
Cyclotella oceallata	-	-	-	1	-	2	+	-	-	-	-	-	-	-	-	-	-	+	-	-	-	-	+	-	-	-
Cyclotella radiosa	-	-	-	2	-	-	-	-	+	-	-	-	-	-	-	1	-	-	-	-	-	-	+	-	+	-
Cymbella affiniformis	-	-	-	-	-	+	-	-	-	-	-	-	-	-	-	-	-	-	-	-	+	-	-	-	+	-
Cymbella cymbiformis	-	-	-	-	-	-	-	-	-	-	-	-	-	-	+	-	+	-	-	-	-	-	-	-	-	-
Cymbella excisa	-	-	-	-	-	+	-	-	-	-	-	-	-	+	-	-	-	-	+	-	-	-	-	+	-	-
Cymbella hustedtii	-	-	-	-	-	1	-	-	-	-	-	-	-	+	-	-	-	-	-	-	-	-	-	-	-	-
Cymbella lanceolata	-	-	-	-	-	-	-	-	-	-	-	-	+	-	1	-	+	-	-	+	-	-	-	-	-	-
Cymbella neocistula	-	-	-	-	-	+	-	-	-	-	-	-	-	-	+	-	+	+	-	-	-	-	-	-	-	-
Cymbella tumida	-	-	-	-	-	-	-	-	-	-	-	-	-	+	+	-	-	-	-	+	-	-	-	-	-	-
Cymbella vulgata	-	-	-	-	-	-	-	-	-	-	-	-	-	-	-	-	+	-	-	-	-	-	-	-	-	-
Diadesma perpusilla	-	+	-	-	-	-	-	-	-	-	-	-	-	-	-	-	-	-	-	-	-	-	-	-	-	-
Diatoma circulare var. *circulare*	-	-	-	-	+	-	-	-	-	-	-	-	-	-	-	-	-	-	-	-	-	-	-	-	-	-
Diatoma moniliformis	-	-	-	-	-	-	-	-	-	-	-	-	-	-	-	-	-	-	-	-	-	-	+	-	-	-
Diatoma tenuis	-	-	-	-	-	-	-	-	-	-	-	-	+	-	-	1	-	-	-	-	-	-	-	-	-	-
Diatoma vulgaris	-	-	-	-	-	+	-	-	-	-	-	-	+	-	-	-	-	-	-	-	-	-	-	-	-	-
Diploneis fontanella	-	-	-	-	+	-	-	-	-	-	-	-	-	-	-	-	-	-	-	-	-	-	-	-	-	-
Diploneis parma	-	-	-	-	+	-	-	-	-	-	-	-	-	-	-	-	-	-	-	-	-	-	-	-	-	-
Discostella pseudostelligera	-	-	-	+	-	-	-	-	-	-	-	-	-	-	-	+	-	-	-	-	1	1	-	-	-	-
Encynopsis microcephala	-	-	-	-	-	-	-	-	-	-	-	-	-	-	-	-	-	-	-	-	-	-	+	-	-	-
Encyonema caespitosum	-	-	-	-	-	-	-	-	-	-	-	-	-	-	+	-	-	-	-	-	-	-	-	-	-	-
Encyonema glacialis	-	-	-	+	-	-	-	-	-	-	-	-	-	-	-	-	-	-	-	-	-	-	-	-	-	-
Encyonema lange- bertalotii	-	-	-	-	-	-	-	-	-	-	-	-	-	-	-	+	-	-	-	-	-	-	-	-	-	-
Encyonema minutum	-	-	-	-	-	-	+	-	-	-	-	+	-	+	-	-	-	-	-	-	-	-	-	-	-	-
Encyonema prostratum	-	-	-	-	+	-	-	-	-	-	-	-	-	-	-	-	-	-	-	-	-	+	-	-	-	-
Encyonema silesiacum	-	+	-	+	-	+	-	-	-	-	-	-	-	-	-	-	-	-	-	-	-	-	-	-	-	-
Encyonema ventricosum	+	1	-	-	+	1	-	-	+	-	+	2	2	-	+	+	+	1	+	+	+	-	1	-	1	-
Encyonema vulgare	-	-	-	-	-	-	-	-	-	-	-	-	-	-	-	-	-	+	-	+	-	-	-	-	-	-
Eolimna minima	1	+	-	-	1	+	+	-	-	-	1	1	-	+	+	+	2	2	2	2	1	+	1	+	1	2
Eolimna subminuscula	-	-	-	-	-	-	-	-	-	-	-	-	-	-	-	-	-	+	-	-	-	+	-	-	-	-
Eolimna tantula	-	-	-	1	+	-	+	-	-	-	+	-	-	-	-	-	+	+	-	-	-	-	-	-	-	+
Epithemia adnata	+	-	-	1	-	-	+	-	+	1	-	-	-	+	-	-	-	-	-	-	-	-	-	-	-	-
Epithemia sorex	-	-	-	-	-	-	1	-	+	1	-	-	-	1	2	-	-	-	-	-	-	-	-	-	-	-
Epithemia turgida	-	-	-	-	-	-	-	-	-	-	-	-	-	-	-	-	-	-	-	1	-	-	-	-	-	-
Eunotia dianae-stitinensis	-	-	-	-	-	-	-	-	-	-	-	-	-	-	-	-	+	-	-	-	-	-	-	-	-	-
Eunotia gracialifalsa	-	-	-	-	-	-	-	-	-	-	-	-	-	-	-	-	-	-	-	2	-	-	-	-	-	-
Eunotia metamonodon	-	-	-	-	-	-	-	-	-	-	-	-	-	-	-	-	-	-	-	2	-	-	-	-	-	-
Eunotia monodontiforma	-	-	-	-	-	-	-	-	-	-	-	-	-	-	-	-	-	-	-	1	-	-	-	-	-	-
Fallacia lenzii	-	-	1	-	-	-	-	-	-	-	-	-	+	-	-	-	-	-	-	+	-	-	-	-	-	-
Fallacia pygmaea	-	-	-	-	-	-	-	-	-	-	-	-	-	-	-	-	-	-	-	-	-	+	-	-	-	-
Fallacia subhamulata	-	-	-	-	1	-	-	-	-	-	-	-	-	-	-	-	-	+	-	-	-	-	+	-	-	-
Fragilaria alpestris	-	-	-	-	+	-	-	-	-	-	-	-	-	-	-	-	-	-	-	-	-	-	-	-	-	-

Fragilaria bidens	-	-	-	-	-	-	-	-	-	-	-	-	-	-	+	+	-	-	-	-	-	-	-	-	-	-
Fragilaria capucina var. *capucina*	-	+	-	-	-	+	-	-	+	-	+	-	-	-	-	+	+	1	-	+	-	+	-	-	+	-
Fragilaria crotonenis	-	-	-	-	-	-	-	-	-	-	+	-	-	-	-	-	-	-	-	-	-	-	-	-	-	-
Fragilaria delicatissima	-	-	-	-	-	-	-	-	-	-	-	-	-	-	-	-	-	+	-	-	-	+	-	-	-	-
Fragilaria dilatata	-	-	-	-	-	-	-	-	-	-	-	-	-	-	-	-	-	-	-	-	-	+	-	-	-	-
Fragilaria famelica	-	-	-	-	-	-	-	-	-	-	-	-	-	-	-	-	-	-	-	-	-	+	-	-	-	-
Fragilaria gracilis	-	-	-	+	-	-	-	-	-	+	-	-	-	-	-	-	-	-	-	-	-	+	-	-	-	-
Fragilaria mesolepta	-	-	-	-	-	-	-	-	+	-	-	-	-	-	-	-	+	+	-	-	-	-	-	-	-	-
Fragilaria nanana	-	-	+	+	-	1	-	-	-	-	-	-	-	-	-	+	-	1	-	-	+	+	-	-	+	-
Fragilaria parasitica var. *parasitica*	+	+	+	-	-	-	-	-	-	-	-	-	-	+	-	-	-	+	-	-	+	-	-	-	-	-
Fragilaria parasitica var. *subconstricta*	-	-	-	-	+	-	-	-	-	-	+	-	+	-	+	-	-	-	+	-	-	-	-	-	-	+
Fragilaria perminuta	-	-	-	-	-	-	-	-	-	-	-	-	-	-	-	-	-	-	-	-	-	-	-	-	+	-
Fragilaria radians	-	-	-	-	-	+	-	-	-	-	-	-	-	-	-	-	-	-	-	-	-	-	-	-	-	-
Fragilaria recapitellata	-	-	-	-	-	-	-	-	-	-	-	-	-	-	-	-	-	-	-	-	+	-	-	-	-	-
Fragilaria rumpens	-	-	-	-	-	+	-	-	-	-	+	-	-	-	+	-	-	+	-	-	-	-	+	-	-	-
Fragilaria tenera	-	1	-	-	-	1	-	-	-	-	-	-	-	-	-	-	-	+	-	+	-	-	-	+	-	-
Fragilaria ulna	-	-	-	-	-	-	-	-	-	-	-	-	+	-	-	-	-	-	-	-	-	-	-	-	-	-
Fragilaria vaucheriae	+	-	-	-	-	-	-	-	+	+	+	-	+	1	+	-	-	1	+	+	+	-	+	-	+	-
Geissleria acceptata	-	-	-	-	-	-	-	-	+	-	-	-	-	-	-	-	1	+	-	-	-	-	-	-	+	-
Geissleria decussis	-	-	-	-	-	-	+	-	-	-	+	-	+	+	-	-	-	+	-	-	-	-	1	+	-	+
Geissleria schoenfeldii	-	-	-	-	-	-	-	-	+	-	-	-	-	+	-	-	-	-	-	-	-	-	-	-	-	-
Gomphonema acuminatum	-	-	-	+	-	-	-	-	-	+	-	-	-	+	+	-	+	+	-	+	-	-	-	-	-	-
Gomphonema angustatum	-	-	-	-	-	-	-	-	-	-	-	-	1	-	+	-	-	+	-	-	-	-	+	-	-	-
Gomphonema augur	+	-	-	-	-	-	-	-	-	-	-	-	-	-	-	-	-	-	-	-	-	-	-	-	-	-
Gomphonema auritum	-	-	-	-	-	-	-	-	-	-	-	-	1	-	+	-	-	-	+	+	-	-	-	-	-	-
Gomphonema brebissonii	-	-	-	-	-	-	-	-	-	-	-	-	-	-	+	-	+	-	-	+	-	-	-	-	+	-
Gomphonema capitatum	-	-	-	-	-	-	-	-	-	-	-	-	-	-	+	-	+	-	-	-	-	-	-	-	-	-
Gomphonema clavatum	-	-	-	-	-	-	-	-	-	-	-	-	-	+	-	-	-	-	-	-	+	-	-	-	-	-
Gomphonema cymbelliclinum	-	-	-	-	-	-	-	-	-	-	-	-	+	-	-	-	-	-	-	-	-	-	-	-	-	-
Gomphonema hebridense	-	-	-	-	-	1	-	-	-	-	-	-	-	-	-	-	-	-	-	-	-	-	-	-	-	-
Gomphonema innocens	-	-	-	-	-	-	-	-	-	-	-	+	-	-	-	-	-	-	-	-	+	+	-	-	-	-
Gomphonema italicum	-	-	-	-	-	-	-	-	-	-	+	-	-	-	-	-	-	-	-	-	-	-	-	-	-	-
Gomphonema lateripunctatum	-	-	-	-	-	+	-	-	-	-	-	-	-	-	-	-	-	-	-	-	-	-	-	-	-	-
Gomphonema micropus	-	-	-	-	-	-	-	-	-	-	-	-	-	-	-	-	-	-	-	-	-	-	-	-	+	-
Gomphonema minusculum	-	-	+	1	-	2	2	-	-	-	-	+	-	-	-	+	-	-	-	1	-	-	-	-	-	-
Gomphonema minutum	-	-	-	+	-	-	-	-	-	-	-	1	-	-	-	-	+	-	-	-	-	-	+	-	+	-
Gomphonema olivaceoides	-	+	-	-	+	-	-	-	+	-	+	+	-	+	-	-	-	-	-	-	-	-	-	-	+	-
Gomphonema olivaceum	-	+	-	-	1	1	+	-	+	+	-	2	2	+	-	-	-	1	-	-	-	+	1	1	+	+
Gomphonema pala	-	-	-	+	-	-	-	-	-	-	+	-	-	+	+	-	-	-	-	-	-	+	-	-	-	-
Gomphonema parvulum var. *parvulum* f. *parvulum*	-	1	1	1	-	1	+	1	+	1	+	1	1	+	1	+	+	2	-	-	1	+	+	+	-	+
Gomphonema pseudoaugur	-	-	+	-	-	-	-	-	-	-	-	-	-	-	-	-	-	-	-	-	-	-	-	-	-	-
Gomphonema pumilum	-	-	-	-	-	2	-	1	-	-	+	+	-	-	+	-	-	-	1	-	-	-	-	-	-	+
Gomphonema subclavatum	-	-	-	+	-	1	-	-	-	-	-	-	-	-	-	-	+	-	-	-	-	-	-	-	-	-
Gomphonema utae	-	-	-	-	+	-	-	-	-	-	-	+	-	-	-	-	-	-	+	-	-	+	-	-	-	-
Gomphonema vibrio	-	-	-	-	-	-	-	-	-	-	-	-	-	-	-	-	-	-	-	-	-	-	-	-	-	+
Gomphosphenia tackei	-	-	-	-	-	-	-	-	-	-	-	-	-	-	-	-	-	-	+	-	-	-	-	-	-	-
Gyrosigma acuminatum	-	-	-	-	+	-	-	-	-	-	-	-	-	-	-	-	-	-	-	-	+	+	+	-	+	+
Gyrosigma attenuatum	-	+	-	-	-	-	-	-	-	-	-	-	-	-	-	-	-	-	-	-	-	-	-	+	-	-
Gyrosigma sciotoense	-	-	-	-	+	-	-	-	-	-	-	-	-	-	-	-	-	-	-	-	+	+	-	-	-	+
Halamphora normanii	-	-	-	-	-	-	-	-	-	-	-	-	-	-	-	-	+	-	-	-	-	-	-	-	-	-
Halamphora oligotraphenta	-	-	-	-	-	-	-	-	-	-	-	-	-	-	-	-	-	-	+	-	-	-	-	-	-	-
Halamphora veneta	-	-	-	-	-	-	-	-	-	-	-	-	-	+	-	-	-	-	-	-	-	-	-	-	-	-
Hipodonta capitata	-	+	-	-	+	-	-	-	-	-	-	-	-	-	-	-	-	+	-	-	-	-	-	-	-	-
Hipodonta hungarica	-	-	-	-	-	-	-	-	-	-	-	-	-	-	-	-	-	-	-	-	-	-	-	+	-	-
Hipodonta lueneburgensis	-	-	-	-	-	-	-	-	-	-	-	-	-	-	-	-	-	-	-	-	-	-	-	+	-	-
Karayevia clevei	+	1	-	-	1	+	+	-	1	2	+	-	+	+	+	+	+	1	+	-	1	1	1	1	1	+
Karayevia kolbei	-	-	-	-	+	-	-	-	-	-	-	-	-	-	-	-	-	+	-	+	-	-	-	+	+	-
Karayevia ploenensis var. *ploenensis*	-	-	-	-	+	-	-	-	-	-	-	-	-	-	-	-	-	-	-	-	+	-	-	-	-	-
Kolbesia gessneri	-	-	-	-	+	-	-	-	+	-	-	-	-	-	-	-	-	-	-	+	-	+	-	-	-	-
Lemnicola hungarica	+	+	-	-	+	-	-	-	-	-	+	-	+	-	-	-	+	+	-	+	+	-	-	-	-	-
Melosira varians	-	-	+	-	+	-	-	-	-	+	+	-	2	1	2	-	2	1	1	+	1	2	2	2	2	1
Meridion circulare var. *circulare*	-	1	-	-	-	-	-	-	-	-	-	-	-	-	-	-	-	-	-	-	-	-	-	-	-	-
Navicula antonii	-	+	-	+	2	1	1	-	1	+	1	+	+	+	2	+	+	+	+	+	+	+	1	+	+	-
Navicula capitatoradiata	+	+	-	-	2	-	-	-	-	-	+	1	2	+	1	-	+	+	-	-	-	+	+	1	+	+
Navicula cari	-	+	-	-	-	-	-	-	-	-	-	-	-	+	-	-	-	-	-	+	+	-	+	-	-	-
Navicula cincta	-	-	-	-	-	-	-	-	-	-	-	-	-	-	-	-	-	-	-	-	+	-	-	-	-	-
Navicula cryptocephala	-	-	-	-	-	+	-	-	-	-	+	-	-	-	-	-	1	+	+	+	1	1	+	-	-	+
Navicula cryptofallax	-	-	-	-	-	-	-	-	-	-	-	-	-	-	-	-	-	-	-	-	+	-	-	-	-	-
Navicula cryptotenella	-	1	1	+	2	+	2	+	1	1	1	1	1	1	2	+	1	1	1	2	1	1	1	1	-	1
Navicula cryptotenelloides	-	-	-	-	-	-	-	+	+	-	-	-	-	-	-	-	-	-	-	-	-	-	-	-	-	-
Navicula exilis	-	-	-	-	-	-	-	-	-	-	-	-	+	-	-	-	-	-	-	-	-	-	-	-	-	+
Navicula germainii	-	-	-	-	-	-	-	-	-	-	-	-	+	-	-	-	-	-	-	-	-	-	+	-	-	-
Navicula gregaria	-	-	-	-	+	-	-	-	-	-	-	-	-	-	-	-	-	+	-	-	-	-	-	+	-	+
Navicula hanseatica	-	-	-	-	-	-	-	-	-	-	-	-	-	-	-	-	-	-	-	-	-	-	-	-	+	-
Navicula lanceolata	-	-	-	-	+	-	-	-	-	-	+	-	-	-	-	-	-	-	-	-	+	-	-	-	-	-
Navicula lundii	-	-	-	+	+	-	-	-	-	-	-	-	-	-	-	-	+	-	+	-	+	+	+	-	-	-
Navicula menisculus	-	-	-	-	-	-	-	-	-	-	-	-	-	-	-	-	-	-	-	+	-	-	-	-	-	-
Navicula moskalii	-	-	-	-	-	-	-	-	-	-	+	-	-	-	-	-	-	+	-	-	-	-	-	-	-	-
Navicula notha	+	-	-	-	-	-	-	-	-	-	-	-	-	-	-	-	-	-	-	-	-	-	-	-	-	-
Navicula oblonga	+	-	-	-	-	-	-	-	-	-	-	-	-	-	-	-	-	-	-	-	-	+	-	-	-	-
Navicula oppugnata	-	-	-	-	-	-	-	-	-	-	-	-	-	-	-	-	-	-	-	-	-	-	+	-	+	+
Navicula radiosa	5	+	2	1	-	-	-	-	-	-	2	-	-	-	+	-	-	+	+	-	-	+	+	-	-	-
Navicula recens	-	-	-	-	+	-	-	-	-	-	-	-	-	-	-	-	-	+	+	-	-	-	+	-	-	-
Navicula reichardtiana	1	+	-	1	+	1	1	+	+	+	+	1	2	+	-	-	-	-	-	-	+	+	+	-	-	+
Navicula reinhardtii	-	+	-	-	-	-	-	-	-	-	+	-	+	-	-	-	-	+	-	-	-	-	-	-	+	-
Navicula rhynchotella	-	-	-	-	-	-	-	-	-	-	-	-	2	-	-	-	-	-	-	-	-	-	-	-	-	-
Navicula slesvicensis	-	+	-	-	-	-	-	-	-	-	-	-	-	-	-	-	-	-	+	-	-	-	-	-	-	-
Navicula subalpina	-	-	-	-	-	-	-	-	-	-	+	-	-	-	-	-	-	-	-	-	-	-	-	-	-	-
Navicula tenelloides	-	-	-	-	-	-	-	-	-	-	-	-	-	-	-	-	-	-	-	-	+	+	-	-	-	-
Navicula tripunctata	-	2	-	-	1	1	-	+	+	+	+	1	1	+	2	+	+	1	1	1	2	2	1	1	1	2
Navicula trivialis	-	-	-	+	-	-	-	-	-	-	-	-	-	-	-	-	-	-	-	-	-	-	-	-	-	-
Navicula upsaliensis	-	-	-	-	-	-	+	-	-	-	-	-	-	-	-	-	-	-	-	-	-	-	-	-	+	-
Navicula veneta	1	-	-	-	-	-	-	-	-	-	-	-	-	-	+	-	-	-	-	-	-	-	-	-	-	-
Navicula wildii	-	-	-	+	-	-	-	-	-	-	-	-	-	-	-	-	-	-	-	-	-	-	-	-	-	-
Nitzschia acicularis	-	-	-	-	-	-	-	-	-	-	-	-	-	-	-	+	-	-	-	-	-	-	-	-	-	-
Nitzschia adamanta	-	-	-	-	-	-	-	-	-	-	-	-	+	-	-	-	-	-	-	-	-	+	-	-	-	-
Nitzschia agnita	+	-	+	-	-	-	-	-	-	-	-	+	-	-	-	+	-	-	-	-	-	-	-	-	+	+
Nitzschia amphibia	-	+	-	-	+	+	+	+	1	+	+	+	1	1	2	+	2	1	1	2	+	+	+	+	1	2

Nitzschia angustata	-	-	-	-	-	-	-	-	-	-	-	-	-	-	-	-	-	-	-	-	-	-	+	-	-	-
Nitzschia archibaldii	-	-	-	-	-	-	-	-	-	-	-	-	-	-	-	-	-	-	-	-	-	+	-	-	-	-
Nitzschia bulnheimiana	-	-	-	-	-	-	-	-	-	-	-	-	+	-	+	-	1	-	-	1	-	-	-	+	-	-
Nitzschia capitellata	-	-	-	-	-	-	-	-	-	-	+	-	-	-	-	+	-	-	-	-	+	+	+	-	-	+
Nitzschia capitellata	-	-	-	-	-	-	-	-	-	-	-	-	-	-	-	-	-	+	-	-	-	-	-	-	-	-
Nitzschia dissipata var. *dissipata*	-	-	+	-	2	-	+	-	-	-	1	+	+	+	1	-	-	-	-	1	-	+	1	-	-	-
Nitzschia dissipata var. *media*	+	-	-	+	2	-	-	-	-	-	1	1	2	+	+	+	+	+	+	+	+	+	-	+	+	-
Nitzschia fibulafissa	-	-	-	-	-	-	-	-	-	-	-	-	1	-	-	-	-	-	-	-	-	-	-	-	-	-
Nitzschia fonticola	-	+	+	-	2	-	-	-	+	-	1	1	2	+	2	1	2	2	+	+	2	2	2	1	1	+
Nitzschia fossilis	-	-	-	-	-	-	-	-	-	-	-	-	-	-	-	-	-	+	-	-	-	-	-	-	-	-
Nitzschia frustulum var. *frustulum*	-	-	-	-	-	-	-	-	-	-	-	-	-	-	-	-	-	-	-	-	-	+	-	+	-	-
Nitzschia frustulum var. *incospicua*	-	-	-	-	-	-	-	-	-	-	-	-	-	-	-	-	-	-	-	-	-	+	-	-	-	-
Nitzschia gracilis	+	-	-	+	-	-	-	-	-	-	-	-	-	+	-	+	-	-	-	-	1	1	+	+	+	+
Nitzschia hantzschiana	-	-	-	-	-	-	-	-	-	+	-	+	-	-	-	-	-	-	-	-	-	-	-	-	-	-
Nitzschia heufleriana	-	-	-	-	-	-	-	-	-	-	-	-	-	-	-	-	-	-	-	-	-	-	-	-	-	+
Nitzschia intermedia	+	+	+	-	-	-	-	-	-	-	-	-	+	+	-	-	-	-	-	-	-	+	+	-	-	-
Nitzschia lacuum	-	-	-	-	-	-	-	-	-	-	-	-	-	-	+	-	-	-	-	-	-	-	-	-	-	-
Nitzschia linearis	+	-	+	-	-	-	-	-	-	-	-	-	-	-	-	-	-	-	-	-	-	-	-	1	-	-
Nitzschia microcephala	-	-	-	-	-	-	-	-	-	-	-	-	-	-	-	-	-	-	-	-	+	-	-	-	-	-
Nitzschia palea var. *palea*	+	-	-	-	-	-	-	-	-	-	-	-	-	-	-	-	-	-	+	-	+	-	-	+	+	-
Nitzschia paleacea	-	-	-	-	-	-	-	-	-	-	-	-	-	-	+	-	-	+	-	-	+	-	-	-	-	-
Nitzschia perminuta	-	-	-	-	-	-	-	-	-	-	+	-	-	+	+	-	+	-	-	-	+	-	+	-	+	+
Nitzschia pura	-	-	-	-	-	-	-	-	-	-	-	-	-	-	-	-	-	-	-	-	-	+	-	-	-	-
Nitzschia radicula	-	+	-	-	-	-	-	-	-	-	-	-	-	-	-	-	-	-	-	-	-	-	-	-	-	-
Nitzschia recta	-	-	+	+	+	-	-	-	-	-	-	-	-	-	-	-	-	+	-	-	+	+	-	+	-	-
Nitzschia sigmoidea	+	-	-	-	-	-	-	-	-	-	-	-	-	-	-	-	-	-	-	-	-	+	-	-	-	+
Nitzschia sociabilis	-	-	-	-	-	-	+	-	-	-	-	-	+	-	-	-	+	-	-	-	+	-	-	+	+	+
Nitzschia subacicularis	-	-	-	-	-	-	-	-	-	-	-	-	-	-	-	1	-	-	-	+	-	-	-	+	-	-
Nitzschia sublinearis	+	-	-	-	-	-	-	-	-	-	-	-	+	-	-	-	-	-	-	-	-	-	-	-	-	-
Nitzschia subtilis	-	-	-	-	-	-	-	-	-	-	-	-	+	-	-	-	-	-	-	-	+	+	-	-	-	-
Nitzschia supralitorea	-	-	-	-	-	-	+	-	-	-	+	-	-	+	2	-	-	-	-	-	-	-	-	-	+	-
Nitzschia tenuis	-	-	-	-	-	-	-	-	-	-	-	-	-	-	-	-	-	-	-	-	-	-	-	+	-	-
Nitzschia vermicularis	-	-	-	-	-	-	-	-	-	-	-	-	-	-	-	-	-	-	-	-	-	-	-	+	+	-
Nitzschia wuellerstorffii	-	+	-	-	-	-	-	-	-	-	-	-	+	-	-	-	-	-	-	-	-	-	-	-	+	-
Pinnularia acrosphaeria	+	-	-	-	-	-	-	-	-	-	-	-	-	-	-	-	-	-	-	-	-	-	-	-	-	-
Pinnularia grunowii	-	+	-	-	-	-	-	-	-	-	-	-	-	-	-	-	-	-	-	-	-	-	-	-	-	-
Pinnularia obscura	-	-	-	-	-	-	-	-	-	-	-	-	-	-	-	-	-	-	-	-	-	-	-	-	-	+
Placoneis clementis	-	-	+	-	-	-	-	-	-	-	-	-	-	-	-	-	-	-	-	-	-	-	-	-	-	-
Placoneis elginensis	-	+	+	-	-	-	-	-	-	-	-	-	-	-	-	-	-	-	-	-	-	-	-	-	-	-
Placoneis pseudanglica	-	-	-	-	-	-	-	-	-	+	-	-	-	-	-	-	-	-	-	-	-	-	-	-	-	-
Planothidium delicatulum	-	-	-	-	-	-	-	-	-	-	-	-	-	-	-	-	-	-	-	-	-	+	-	-	-	+
Planothidium dubium	-	+	-	-	1	-	-	-	+	-	-	-	1	+	-	-	+	+	+	-	-	+	+	+	-	-
Planothidium frequentissimum	+	1	+	-	1	+	+	-	2	2	+	-	+	1	-	-	1	-	1	+	+	1	1	+	1	1
Planothidium granum	-	-	-	-	-	-	-	-	-	-	-	-	-	-	-	-	+	-	-	-	-	-	+	-	-	-
Planothidium joursacense	-	-	-	-	-	-	-	-	-	-	-	-	-	-	-	-	-	-	-	-	-	-	-	+	-	-
Planothidium lanceolatum	+	1	2	-	+	-	-	-	+	1	+	-	+	1	-	-	1	+	-	-	-	-	-	-	+	+
Planothidium minuttissimum	-	-	-	-	-	-	-	-	-	-	-	-	-	-	-	-	-	-	+	-	-	+	-	-	-	-
Planothidium rostratum	-	+	+	-	1	-	-	-	+	+	+	-	+	-	-	+	-	-	-	-	-	+	-	-	+	-
Platessa conspicua	-	-	-	-	-	-	-	-	+	+	-	-	-	-	-	-	-	-	-	+	-	+	-	-	-	-
Prestauroneis integra	-	-	-	-	-	-	-	-	-	-	-	-	-	-	+	-	-	-	-	-	-	-	-	-	-	-
Psammothidium bioretii	-	-	-	-	-	+	-	-	-	-	-	-	-	-	-	-	-	-	-	-	-	-	-	-	-	-
Psammothidium daonense	-	-	-	-	-	-	-	-	-	-	-	+	-	-	-	-	-	-	-	-	-	-	-	-	-	-
Psammothidium helveticum	-	-	-	-	-	-	-	-	-	-	-	-	-	-	-	-	-	-	-	-	-	+	-	-	-	+
Psammothidium lauenburgianum	-	-	-	-	-	-	-	-	-	-	-	-	-	-	-	-	-	-	-	+	-	1	-	-	-	-
Psammothidium marginulatum	-	-	-	-	-	-	-	-	-	-	-	+	-	-	-	-	-	-	-	-	-	-	-	-	-	-
Psammothidium subatomoides	-	-	-	-	-	-	-	-	-	-	-	-	-	-	-	-	-	-	+	-	+	-	+	-	+	-
Pseudostaurosira binodis	-	-	-	-	+	-	+	-	-	-	-	-	-	1	1	-	-	-	-	-	-	+	+	+	1	+
Pseudostaurosira brevistriata	-	1	-	-	+	2	2	-	1	2	1	-	+	2	2	+	1	1	2	2	+	1	1	2	2	1
Reimeria sinuata	-	-	-	-	-	-	-	-	+	-	-	-	-	-	-	-	-	+	-	-	-	+	-	-	-	-
Reimeria uniseriata	-	+	-	-	-	+	+	-	-	-	-	-	-	-	-	-	-	-	-	-	-	-	-	-	-	-
Rhoicosphenia abbreviata	-	1	-	-	+	2	2	+	2	2	-	-	-	1	1	-	+	-	2	2	-	-	-	+	-	+
Sellaphora bacillum	-	-	-	-	-	-	-	-	-	-	-	-	-	-	-	-	-	-	-	-	-	-	-	-	-	+
Sellaphora blackfordensis	-	-	-	-	-	-	-	-	-	-	-	-	-	-	-	-	-	-	-	+	-	-	-	-	-	-
Sellaphora joubaudii	-	-	-	-	-	-	-	-	-	-	-	-	-	+	-	-	+	-	+	+	-	-	-	-	-	+
Sellaphora mutata	-	-	-	-	-	-	-	-	-	-	-	-	-	-	-	-	-	-	-	-	-	-	+	-	-	-
Sellaphora mutatoides	-	+	-	-	-	-	-	-	-	-	-	-	-	+	-	-	-	+	-	-	-	-	-	-	-	-
Sellaphora pupula sensu lato	-	-	+	-	-	-	-	-	-	-	-	-	-	-	-	-	+	-	-	-	-	-	-	-	-	-
Sellaphora pupula sensu lato blackfordensis	-	-	-	-	-	-	-	-	-	-	-	-	-	+	-	-	-	-	-	-	-	-	-	-	-	-
Sellaphora seminulum	-	-	+	-	+	-	-	-	+	-	2	-	1	2	-	-	1	2	-	-	-	-	-	-	-	-
Skeletonema potamos	-	-	-	-	-	-	-	-	-	-	-	-	-	-	-	-	-	-	-	-	1	-	-	-	-	-
Stauroneis acidoclinata	-	-	-	-	-	-	-	-	-	-	-	-	-	-	-	-	-	-	-	-	-	+	-	-	-	-
Stauroneis gracilis	-	-	-	+	-	-	-	-	-	-	-	-	-	-	-	-	-	-	-	-	-	-	-	-	-	-
Stauroneis kriegeri	-	-	-	-	-	-	-	-	+	-	-	-	-	-	-	-	-	-	-	-	-	-	-	-	-	-
Stauroneis smithii	-	-	-	-	-	-	-	-	-	-	-	-	-	-	-	-	-	-	-	-	+	+	-	-	-	-
Staurosira construens	2	+	2	-	2	-	2	-	2	-	-	-	1	2	-	-	+	1	1	+	-	1	2	1	2	1
Staurosira subsalina	-	-	-	-	-	-	-	-	-	-	-	-	-	-	-	-	-	-	-	-	-	-	-	-	1	-
Staurosira venter	-	+	2	+	2	1	+	-	+	-	+	-	-	2	+	-	1	1	2	1	2	2	1	2	1	1
Staurosirella martyi	-	-	-	-	-	-	-	-	-	-	-	-	-	-	-	-	-	-	-	-	-	-	+	+	+	-
Staurosirella pinnata	1	2	-	-	2	+	2	-	1	2	1	-	+	2	-	+	+	+	-	+	+	1	-	2	1	+
Stephanodiscus alpinus	-	-	-	-	-	-	-	-	-	-	-	-	-	-	-	+	-	-	-	-	1	2	-	-	-	-
Stephanodiscus hantzschii	-	-	1	1	+	-	-	-	-	-	+	-	-	-	-	2	1	2	1	1	2	2	2	2	2	2
Stephanodiscus medius	-	-	-	-	-	-	-	-	-	-	-	-	-	-	-	+	-	-	-	-	-	-	-	-	-	-
Stephanodiscus minutulus	-	-	-	-	-	-	-	-	-	-	-	-	-	-	-	2	-	-	-	-	-	+	+	-	+	+
Stephanodiscus neoastrea	-	-	-	+	-	-	-	-	+	+	-	-	-	-	-	2	-	-	-	-	+	+	+	-	+	+
Stephanodiscus parvus	-	1	-	+	-	-	-	-	-	-	-	-	-	-	-	1	-	-	-	-	-	-	+	-	-	-
Stephanodiscus rotula	-	-	+	-	+	-	+	-	-	-	-	-	+	-	-	2	-	-	-	+	1	+	-	-	-	-
Surirella amphioxys	-	-	-	-	+	-	-	-	-	-	-	-	-	-	-	-	-	-	-	-	-	-	-	-	-	-
Surirella angusta	-	-	-	-	+	-	-	-	-	-	-	-	+	-	-	-	-	-	-	-	-	+	-	-	-	-
Surirella minuta	-	-	-	-	-	-	-	-	-	-	-	-	+	-	-	-	-	-	-	-	-	-	-	+	-	-
Tabellaria flocculosa	-	-	+	-	-	1	2	1	2	1	2	-	-	-	-	-	-	-	-	-	-	-	-	-	-	-
Tabularia fasciculata	-	-	-	-	-	-	-	-	-	-	-	-	-	-	-	-	-	-	-	+	-	-	-	-	-	-
Thalassiosira pseudonana	-	-	-	-	-	-	-	-	-	-	-	-	-	-	-	-	-	-	-	-	-	-	+	-	-	-
Ulnaria acus	+	-	1	1	+	+	+	2	+	-	+	+	+	+	+	+	+	+	+	+	1	+	+	2	+	+
Ulnaria biceps	+	-	-	-	+	-	-	-	-	-	1	-	-	+	-	-	+	+	-	1	+	1	+	+	-	-
Ulnaria delicatissima var. *angustissima*	-	-	-	+	-	-	-	-	-	-	-	-	-	-	+	-	-	-	-	-	-	-	-	-	+	+
Ulnaria ulna	-	-	-	-	-	-	-	-	-	-	-	-	-	-	+	-	-	-	-	-	-	+	-	1	+	+
