# Supplementary material for: Is it possible to optimise the labour and time intensity of diatom analyses for determination of the Polish Diatom Indices (IO, IOJ)?
Source: Environ Monit Assess. 2022 Nov 3;195(1):64. doi: 10.1007/s10661-022-10676-7 (PMC9633445; doi:10.1007/s10661-022-10676-7)
Supplement: Supplementary file 3 — Supplementary file3 (PDF 231 KB) [file 10661_2022_10676_MOESM3_ESM.pdf]

Supplementary material 3

Supplementary Table E Results of drew values (20 x each counting sum) of Polish Diatom Index for rivers (IO) for both river systems

RIVER INA

		I1	I2	I3	I4	I5a	I5b	I6	I7	I8	I9a	I9b	I10	I11	I12	I13	I14	I15	I16	I17	I18	I19	I20	I21	I22	I23	I24
DRAWS UP TO 50 VALVES	Draw 1	0.734	0.687	0.610	0.676	0.605	0.646	0.504	0.529	0.558	0.510	0.488	0.561	0.497	0.532	0.476	0.564	0.583	0.561	0.511	0.460	0.504	0.575	0.561	0.472	0.573	0.331
	Draw 2	0.754	0.678	0.601	0.650	0.578	0.675	0.568	0.521	0.560	0.538	0.468	0.551	0.487	0.484	0.439	0.499	0.496	0.551	0.542	0.526	0.505	0.520	0.551	0.481	0.591	0.372
	Draw 3	0.756	0.689	0.674	0.621	0.580	0.637	0.522	0.546	0.493	0.475	0.505	0.441	0.550	0.530	0.466	0.576	0.462	0.441	0.489	0.536	0.560	0.559	0.441	0.464	0.557	0.382
	Draw 4	0.759	0.681	0.593	0.681	0.619	0.667	0.534	0.551	0.561	0.536	0.507	0.517	0.472	0.411	0.373	0.573	0.480	0.517	0.542	0.521	0.510	0.539	0.517	0.458	0.571	0.440
	Draw 5	0.777	0.670	0.574	0.635	0.555	0.650	0.550	0.553	0.509	0.523	0.480	0.555	0.533	0.479	0.497	0.549	0.518	0.555	0.546	0.541	0.521	0.526	0.555	0.494	0.574	0.355
	Draw 6	0.767	0.661	0.600	0.646	0.636	0.654	0.558	0.593	0.570	0.538	0.447	0.510	0.535	0.467	0.469	0.589	0.504	0.510	0.550	0.491	0.516	0.502	0.510	0.445	0.602	0.399
	Draw 7	0.771	0.659	0.592	0.602	0.564	0.616	0.492	0.548	0.538	0.521	0.485	0.552	0.503	0.464	0.452	0.521	0.490	0.552	0.526	0.523	0.541	0.529	0.552	0.464	0.545	0.408
	Draw 8	0.780	0.686	0.654	0.658	0.588	0.626	0.532	0.578	0.535	0.478	0.487	0.520	0.531	0.438	0.419	0.562	0.486	0.520	0.524	0.515	0.508	0.499	0.520	0.473	0.552	0.395
	Draw 9	0.754	0.693	0.619	0.671	0.584	0.633	0.492	0.529	0.501	0.521	0.509	0.573	0.448	0.480	0.464	0.603	0.463	0.573	0.498	0.554	0.512	0.502	0.573	0.469	0.514	0.359
	Draw 10	0.716	0.683	0.615	0.621	0.630	0.664	0.498	0.549	0.520	0.508	0.516	0.516	0.531	0.468	0.473	0.596	0.460	0.516	0.491	0.530	0.552	0.556	0.516	0.376	0.521	0.416
	Draw 11	0.749	0.713	0.655	0.623	0.566	0.679	0.576	0.553	0.566	0.490	0.508	0.517	0.417	0.450	0.487	0.528	0.501	0.517	0.516	0.556	0.482	0.506	0.517	0.419	0.566	0.415
	Draw 12	0.805	0.629	0.658	0.615	0.605	0.636	0.459	0.568	0.496	0.559	0.492	0.505	0.498	0.507	0.455	0.498	0.488	0.505	0.473	0.516	0.460	0.509	0.505	0.489	0.536	0.388
	Draw 13	0.795	0.686	0.599	0.596	0.631	0.621	0.531	0.533	0.503	0.475	0.535	0.527	0.552	0.433	0.433	0.516	0.508	0.527	0.529	0.495	0.524	0.552	0.527	0.497	0.534	0.301
	Draw 14	0.777	0.620	0.597	0.607	0.599	0.645	0.492	0.554	0.518	0.509	0.460	0.521	0.499	0.491	0.464	0.492	0.499	0.521	0.521	0.568	0.514	0.540	0.521	0.464	0.544	0.398
	Draw 15	0.749	0.636	0.610	0.591	0.588	0.633	0.496	0.558	0.521	0.533	0.489	0.522	0.510	0.475	0.450	0.539	0.477	0.522	0.551	0.508	0.481	0.498	0.522	0.503	0.529	0.374
	Draw 16	0.756	0.639	0.601	0.629	0.589	0.658	0.527	0.614	0.489	0.479	0.508	0.582	0.511	0.447	0.423	0.495	0.542	0.582	0.487	0.546	0.459	0.525	0.582	0.441	0.508	0.335
	Draw 17	0.764	0.649	0.607	0.539	0.576	0.653	0.517	0.514	0.511	0.535	0.503	0.485	0.466	0.478	0.446	0.562	0.478	0.485	0.539	0.529	0.562	0.485	0.485	0.477	0.525	0.306
	Draw 18	0.787	0.669	0.596	0.603	0.569	0.598	0.517	0.570	0.518	0.547	0.470	0.501	0.484	0.491	0.463	0.564	0.513	0.501	0.531	0.549	0.501	0.526	0.501	0.439	0.569	0.420
	Draw 19	0.742	0.744	0.663	0.618	0.522	0.622	0.499	0.587	0.512	0.482	0.456	0.569	0.510	0.461	0.417	0.551	0.460	0.569	0.508	0.550	0.518	0.555	0.569	0.482	0.532	0.385
	Draw 20	0.807	0.698	0.605	0.615	0.580	0.647	0.560	0.554	0.528	0.523	0.452	0.488	0.445	0.458	0.402	0.584	0.494	0.488	0.569	0.524	0.501	0.453	0.488	0.467	0.523	0.405
DRAWS UP TO 100 VALVES	Draw 1	0.734	0.668	0.614	0.620	0.569	0.665	0.521	0.577	0.514	0.535	0.498	0.521	0.483	0.486	0.477	0.534	0.533	0.521	0.520	0.541	0.515	0.500	0.512	0.456	0.536	0.409
	Draw 2	0.780	0.645	0.604	0.610	0.569	0.642	0.535	0.573	0.517	0.527	0.493	0.526	0.490	0.483	0.456	0.545	0.511	0.526	0.515	0.526	0.534	0.538	0.509	0.500	0.555	0.391
	Draw 3	0.801	0.659	0.636	0.624	0.607	0.635	0.520	0.561	0.529	0.498	0.512	0.508	0.485	0.430	0.436	0.526	0.489	0.508	0.549	0.511	0.509	0.510	0.491	0.465	0.538	0.393
	Draw 4	0.796	0.638	0.628	0.627	0.573	0.661	0.504	0.554	0.525	0.507	0.512	0.546	0.543	0.494	0.455	0.565	0.490	0.546	0.527	0.528	0.540	0.519	0.526	0.492	0.514	0.413
	Draw 5	0.756	0.670	0.623	0.614	0.560	0.648	0.498	0.573	0.539	0.518	0.521	0.532	0.502	0.478	0.463	0.571	0.500	0.532	0.527	0.529	0.494	0.509	0.538	0.455	0.525	0.397
	Draw 6	0.766	0.649	0.614	0.618	0.593	0.660	0.514	0.563	0.509	0.474	0.472	0.551	0.499	0.479	0.461	0.575	0.503	0.551	0.482	0.506	0.484	0.532	0.497	0.474	0.549	0.368
	Draw 7	0.763	0.689	0.623	0.602	0.589	0.650	0.533	0.534	0.520	0.512	0.492	0.546	0.507	0.485	0.444	0.563	0.521	0.546	0.503	0.561	0.496	0.537	0.527	0.521	0.520	0.400
	Draw 8	0.793	0.675	0.619	0.605	0.589	0.674	0.536	0.527	0.506	0.490	0.488	0.513	0.489	0.464	0.468	0.561	0.481	0.513	0.483	0.540	0.543	0.521	0.525	0.481	0.553	0.395
	Draw 9	0.766	0.671	0.629	0.639	0.560	0.656	0.553	0.530	0.517	0.539	0.485	0.578	0.532	0.460	0.457	0.562	0.487	0.578	0.521	0.523	0.530	0.541	0.521	0.501	0.544	0.383
	Draw 10	0.758	0.621	0.620	0.628	0.566	0.647	0.486	0.558	0.544	0.527	0.499	0.540	0.458	0.467	0.468	0.522	0.524	0.540	0.513	0.516	0.513	0.527	0.520	0.483	0.547	0.377
	Draw 11	0.756	0.673	0.617	0.622	0.583	0.651	0.520	0.576	0.496	0.487	0.488	0.526	0.489	0.456	0.473	0.570	0.505	0.526	0.501	0.546	0.483	0.532	0.518	0.497	0.509	0.389
	Draw 12	0.777	0.679	0.594	0.629	0.585	0.639	0.527	0.528	0.530	0.526	0.482	0.521	0.499	0.444	0.457	0.554	0.506	0.521	0.518	0.529	0.501	0.537	0.514	0.476	0.554	0.405
	Draw 13	0.790	0.697	0.639	0.619	0.573	0.662	0.533	0.531	0.549	0.526	0.495	0.564	0.475	0.480	0.464	0.536	0.498	0.564	0.506	0.515	0.510	0.551	0.521	0.446	0.540	0.447
	Draw 14	0.776	0.640	0.595	0.657	0.589	0.652	0.545	0.570	0.527	0.490	0.475	0.515	0.499	0.471	0.473	0.528	0.505	0.515	0.494	0.529	0.509	0.507	0.504	0.458	0.535	0.429
	Draw 15	0.797	0.681	0.621	0.623	0.593	0.640	0.561	0.570	0.519	0.517	0.470	0.538	0.495	0.479	0.471	0.577	0.493	0.538	0.520	0.522	0.523	0.526	0.524	0.475	0.580	0.414
	Draw 16	0.781	0.671	0.607	0.657	0.551	0.663	0.559	0.560	0.528	0.497	0.469	0.511	0.478	0.443	0.469	0.561	0.553	0.511	0.500	0.520	0.497	0.516	0.509	0.449	0.535	0.395
	Draw 17	0.794	0.639	0.591	0.637	0.595	0.625	0.519	0.607	0.535	0.507	0.462	0.541	0.510	0.472	0.468	0.543	0.508	0.541	0.505	0.482	0.511	0.511	0.504	0.478	0.550	0.379
	Draw 18	0.776	0.689	0.599	0.618	0.599	0.647	0.483	0.578	0.493	0.507	0.480	0.556	0.497	0.485	0.450	0.578	0.523	0.556	0.504	0.521	0.482	0.530	0.493	0.483	0.558	0.376
	Draw 19	0.775	0.698	0.609	0.635	0.575	0.650	0.501	0.578	0.539	0.483	0.489	0.555	0.473	0.452	0.449	0.553	0.468	0.555	0.500	0.554	0.506	0.530	0.514	0.476	0.536	0.411
	Draw 20	0.789	0.674	0.623	0.562	0.604	0.639	0.526	0.576	0.545	0.544	0.488	0.524	0.496	0.446	0.418	0.581	0.474	0.524	0.534	0.518	0.522	0.503	0.509	0.488	0.533	0.412
DRAWS UP TO 150 VALVES	Draw 1	0.784	0.671	0.604	0.612	0.582	0.648	0.534	0.560	0.548	0.520	0.477	0.522	0.517	0.471	0.461	0.515	0.514	0.508	0.503	0.535	0.502	0.525	0.495	0.499	0.534	0.398
	Draw 2	0.768	0.690	0.607	0.611	0.584	0.653	0.525	0.559	0.529	0.522	0.477	0.549	0.486	0.461	0.443	0.551	0.500	0.511	0.510	0.523	0.538	0.549	0.506	0.490	0.558	0.398
	Draw 3	0.757	0.643	0.619	0.632	0.592	0.661	0.516	0.560	0.514	0.509	0.479	0.532	0.495	0.457	0.464	0.536	0.505	0.507	0.498	0.541	0.509	0.531	0.530	0.458	0.532	0.419
	Draw 4	0.774	0.675	0.615	0.621	0.582	0.660	0.517	0.558	0.526	0.521	0.492	0.567	0.493	0.454	0.465	0.537	0.524	0.504	0.509	0.530	0.521	0.534	0.537	0.475	0.536	0.373
	Draw 5	0.779	0.679	0.643	0.614	0.585	0.656	0.544	0.574	0.534	0.502	0.483	0.532	0.494	0.459	0.473	0.568	0.513	0.529	0.520	0.527	0.497	0.486	0.514	0.470	0.553	0.396
	Draw 6	0.764	0.669	0.595	0.602	0.582	0.641	0.525	0.564	0.524	0.520	0.476	0.546	0.501	0.470	0.451	0.537	0.512	0.477	0.494	0.508	0.506	0.525	0.533	0.478	0.548	0.379
	Draw 7	0.756	0.686	0.623	0.622	0.588	0.636	0.528	0.558	0.521	0.511	0.476	0.513	0.502	0.489	0.456	0.574	0.512	0.509	0.499	0.523	0.524	0.533	0.531	0.472	0.539	0.376
	Draw 8	0.748	0.671	0.607	0.624	0.580	0.657	0.537	0.569	0.524	0.530	0.483	0.528	0.521	0.456	0.465	0.524	0.497	0.474	0.497	0.515	0.513	0.513	0.523	0.477	0.532	0.404
	Draw 9	0.786	0.689	0.614	0.621	0.602	0.640	0.547	0.554	0.534	0.514	0.480	0.528	0.490	0.468	0.469	0.561	0.503	0.523	0.509	0.537	0.535	0.516	0.528	0.472	0.525	0.381
	Draw 10	0.779	0.676	0.633	0.607	0.581	0.648	0.519	0.562	0.525	0.517	0.493	0.542	0.497	0.469	0.439	0.557	0.506	0.506	0.492	0.518	0.519	0.501	0.537	0.455	0.532	0.389
	Draw 11	0.773	0.637	0.626	0.618	0.567	0.635	0.536	0.546	0.524	0.513	0.486	0.535	0.484	0.482	0.453	0.549	0.498	0.506	0.520	0.508	0.502	0.494	0.513	0.472	0.545	0.389
	Draw 12	0.770	0.639	0.585	0.619	0.583	0.658	0.504	0.571	0.531	0.535	0.476	0.505	0.482	0.481	0.450	0.549	0.499	0.502	0.497	0.511	0.503	0.523	0.535	0.479	0.542	0.384
	Draw 13	0.779	0.684	0.616	0.644	0.588	0.658	0.520	0.574	0.521	0.510	0.494	0.547	0.479	0.454	0.445	0.555	0.508	0.496	0.515	0.544	0.501	0.503	0.516	0.466	0.543	0.379
	Draw 14	0.765	0.679	0.608	0.618	0.590	0.653	0.517	0.556	0.526	0.509	0.496	0.517	0.497	0.475	0.458	0.549	0.494	0.502	0.525	0.528	0.496	0.504	0.530	0.472	0.537	0.394
	Draw 15	0.762	0.652	0.630	0.631	0.575	0.633	0.532	0.558	0.521	0.509	0.490	0.526	0.508	0.489	0.456	0.532	0.512	0.488	0.517	0.532	0.490	0.524	0.509	0.464	0.553	0.371
	Draw 16	0.787	0.662	0.617	0.625	0.569	0.650	0.544	0.550	0.506	0.522	0.493	0.511	0.486	0.465	0.473	0.535	0.517	0.510	0.514	0.514	0.494	0.500	0.517	0.500	0.550	0.370
	Draw 17	0.773	0.662	0.616	0.626	0.593	0.669	0.533	0.563	0.534	0.511	0.509	0.526	0.489	0.456	0.452	0.552	0.513	0.519	0.508	0.507	0.512	0.507	0.519	0.466	0.540	0.374
	Draw 18	0.768	0.685	0.618	0.618	0.579	0.650	0.538	0.565	0.535	0.530	0.485	0.548	0.472	0.483	0.456	0.513	0.527	0.512	0.521	0.509	0.513	0.511	0.504	0.471	0.525	0.431
	Draw 19	0.790	0.676	0.623	0.636	0.599	0.652	0.529	0.551	0.512	0.533	0.487	0.526	0.468	0.481	0.457	0.528	0.504	0.523	0.532	0.526	0.512	0.515	0.499	0.483	0.539	0.389
	Draw 20	0.759	0.668	0.606	0.626	0.595	0.633	0.539	0.575	0.531	0.511	0.479	0.544	0.470	0.468	0.443	0.541	0.496	0.501	0.527	0.539	0.500	0.515	0.540	0.475	0.556	0.377
DRAWS UP TO 200 VALVES	Draw 1	0.779	0.657	0.628	0.627	0.585	0.644	0.531	0.552	0.524	0.504	0.482	0.520	0.465	0.463	0.446	0.550	0.531	0.496	0.503	0.530	0.505	0.526	0.521	0.478	0.539	0.388
	Draw 2	0.785	0.673	0.616	0.605	0.585	0.645	0.515	0.556	0.526	0.525	0.486	0.544	0.489	0.469	0.450	0.558	0.505	0.505	0.513	0.553	0.502	0.522	0.523	0.477	0.546	0.375
	Draw 3	0.767	0.660	0.612	0.647	0.584	0.658	0.517	0.561	0.521	0.514	0.487	0.532	0.482	0.467	0.474	0.525	0.501	0.492	0.512	0.531	0.525	0.519	0.517	0.478	0.546	0.391
	Draw 4	0.786	0.667	0.605	0.615	0.585	0.647	0.527	0.563	0.526	0.501	0.484	0.529	0.498	0.458	0.447	0.552	0.484	0.503	0.518	0.529	0.508	0.523	0.527	0.467	0.533	0.358
	Draw 5	0.789	0.648	0.617	0.628	0.582	0.648	0.514	0.556	0.525	0.503	0.478	0.537	0.481	0.462	0.448	0.546	0.510	0.505	0.507	0.527	0.488	0.512	0.518	0.464	0.548	0.391
	Draw 6	0.781	0.672	0.622	0.617	0.586	0.652	0.516	0.548	0.534	0.513	0.480	0.533	0.479	0.468	0.455	0.556	0.497	0.504	0.510	0.524	0.514	0.514	0.536	0.471	0.544	0.405
	Draw 7	0.763	0.679	0.609	0.624	0.590	0.639	0.523	0.551	0.525	0.508	0.489	0.529	0.495	0.475	0.452	0.557	0.514	0.511	0.516	0.538	0.505	0.533	0.530	0.460	0.546	0.364
	Draw 8	0.761	0.666	0.629	0.610	0.585	0.642	0.519	0.559	0.522	0.519	0.487	0.520	0.482	0.476	0.456	0.532	0.519	0.473	0.514	0.522	0.497	0.525	0.517	0.474	0.541	0.416
	Draw 9	0.778	0.670	0.623	0.610	0.584	0.640	0.547	0.566	0.523	0.512	0.485	0.531	0.491	0.465	0.470	0.533	0.518	0.496	0.506	0.533	0.491	0.508	0.524	0.481	0.539	0.392
	Draw 10	0.764	0.665	0.613	0.605	0.585	0.650	0.530	0.561	0.527	0.518	0.485	0.533	0.501	0.473	0.461	0.535	0.529	0.511	0.507	0.527	0.512	0.503	0.517	0.464	0.543	0.413
	Draw 11	0.771	0.658	0.601	0.620	0.583	0.649	0.531	0.553	0.522	0.515	0.482	0.544	0.487	0.479	0.461	0.541	0.516	0.505	0.506	0.527	0.498	0.514	0.515	0.463	0.537	0.380
	Draw 12	0.767	0.686	0.614	0.647	0.586	0.661	0.548	0.566	0.519	0.504	0.483	0.523	0.487	0.460	0.449	0.546	0.491	0.495	0.512	0.534	0.519	0.523	0.514	0.467	0.540	0.381
	Draw 13	0.777	0.659	0.617	0.630	0.586	0.656	0.514	0.560	0.533	0.507	0.482	0.529	0.502	0.463	0.455	0.539	0.513	0.522	0.502	0.524	0.526	0.511	0.522	0.495	0.550	0.375
	Draw 14	0.765	0.661	0.600	0.611	0.588	0.643	0.542	0.561	0.519	0.509	0.486	0.520	0.477	0.478	0.496	0.535	0.504	0.490	0.514	0.531	0.514	0.525	0.535	0.461	0.533	0.380
	Draw 15	0.769	0.658	0.603	0.624	0.583	0.641	0.526	0.567	0.529	0.515	0.484	0.510	0.487	0.470	0.457	0.560	0.501	0.519	0.488	0.515	0.512	0.514	0.531	0.482	0.541	0.396
	Draw 16	0.774	0.655	0.608	0.625	0.587	0.647	0.534	0.560	0.512	0.512	0.486	0.538	0.484	0.476	0.485	0.529	0.518	0.500	0.518	0.536	0.512	0.519	0.517	0.468	0.552	0.382
	Draw 17	0.762	0.660	0.629	0.631	0.587	0.644	0.525	0.556	0.522	0.514	0.483	0.537	0.493	0.488	0.479	0.543	0.510	0.521	0.533	0.529	0.491	0.505	0.510	0.486	0.536	0.366
	Draw 18	0.780	0.649	0.621	0.620	0.588	0.646	0.526	0.557	0.531	0.513	0.484	0.538	0.493	0.482	0.456	0.549	0.505	0.486	0.498	0.528	0.512	0.525	0.512	0.462	0.543	0.365
	Draw 19	0.775	0.659	0.592	0.606	0.584	0.652	0.517	0.554	0.526	0.513	0.481	0.523	0.485	0.465	0.473	0.538	0.512	0.487	0.496	0.530	0.515	0.511	0.522	0.476	0.546	0.382
	Draw 20	0.776	0.668	0.620	0.627	0.582	0.638	0.535	0.551	0.514	0.525	0.485	0.518	0.492	0.465	0.455	0.543	0.529	0.491	0.506	0.522	0.503	0.511	0.520	0.472	0.543	0.388
DRAWS UP TO 250 VALVES	Draw 1	0.779	0.691	0.618	0.637	0.573	0.641	0.524	0.550	0.511	0.517	0.478	0.537	0.481	0.474	0.458	0.546	0.503	0.501	0.514	0.521	0.518	0.517	0.472	0.473	0.542	0.389
	Draw 2	0.778	0.679	0.627	0.667	0.569	0.659	0.522	0.562	0.523	0.511	0.492	0.532	0.491	0.472	0.464	0.539	0.508	0.494	0.507	0.528	0.522	0.526	0.481	0.478	0.537	0.381
	Draw 3	0.777	0.660	0.611	0.657	0.572	0.656	0.529	0.549	0.530	0.520	0.489	0.522	0.490	0.470	0.466	0.544	0.507	0.509	0.516	0.500	0.508	0.503	0.464	0.499	0.538	0.387
	Draw 4	0.763	0.675	0.619	0.674	0.577	0.629	0.540	0.548	0.520	0.515	0.484	0.528	0.488	0.465	0.454	0.529	0.510	0.485	0.503	0.530	0.502	0.506	0.458	0.485	0.554	0.393
	Draw 5	0.775	0.679	0.618	0.690	0.605	0.647	0.528	0.555	0.516	0.505	0.479	0.527	0.497	0.469	0.456	0.559	0.510	0.508	0.508	0.531	0.507	0.502	0.494	0.470	0.541	0.389
	Draw 6	0.771	0.671	0.604	0.665	0.583	0.671	0.528	0.550	0.520	0.512	0.480	0.523	0.490	0.473	0.462	0.544	0.513	0.495	0.507	0.532	0.517	0.509	0.445	0.476	0.538	0.399
	Draw 7	0.772	0.670	0.617	0.692	0.586	0.648	0.525	0.559	0.531	0.516	0.469	0.534	0.497	0.469	0.476	0.539	0.501	0.504	0.510	0.520	0.505	0.518	0.464	0.465	0.545	0.400
	Draw 8	0.783	0.665	0.621	0.668	0.622	0.662	0.533	0.552	0.536	0.512	0.473	0.517	0.482	0.472	0.459	0.540	0.504	0.497	0.512	0.532	0.511	0.526	0.473	0.469	0.530	0.391
	Draw 9	0.775	0.655	0.603	0.688	0.583	0.626	0.529	0.548	0.518	0.509	0.494	0.530	0.484	0.470	0.454	0.562	0.501	0.495	0.494	0.527	0.508	0.521	0.469	0.471	0.534	0.398
	Draw 10	0.773	0.688	0.614	0.658	0.590	0.654	0.530	0.556	0.522	0.512	0.481	0.547	0.481	0.469	0.464	0.535	0.507	0.502	0.506	0.527	0.501	0.518	0.376	0.476	0.542	0.399
	Draw 11	0.787	0.682	0.614	0.640	0.591	0.641	0.532	0.559	0.542	0.514	0.496	0.528	0.495	0.475	0.442	0.546	0.501	0.493	0.514	0.534	0.515	0.519	0.419	0.478	0.561	0.392
	Draw 12	0.754	0.651	0.619	0.645	0.598	0.650	0.530	0.554	0.522	0.513	0.500	0.520	0.491	0.475	0.468	0.530	0.505	0.506	0.518	0.539	0.495	0.529	0.489	0.468	0.553	0.388
	Draw 13	0.784	0.685	0.611	0.669	0.612	0.656	0.526	0.562	0.527	0.512	0.480	0.544	0.490	0.464	0.457	0.535	0.500	0.496	0.506	0.532	0.511	0.511	0.497	0.485	0.542	0.358
	Draw 14	0.780	0.664	0.618	0.650	0.587	0.648	0.523	0.569	0.538	0.510	0.484	0.528	0.487	0.469	0.468	0.555	0.494	0.504	0.508	0.519	0.516	0.530	0.464	0.482	0.543	0.387
	Draw 15	0.784	0.655	0.625	0.676	0.590	0.671	0.512	0.556	0.514	0.516	0.487	0.514	0.503	0.469	0.466	0.543	0.510	0.508	0.516	0.522	0.519	0.519	0.503	0.485	0.547	0.378
	Draw 16	0.777	0.665	0.613	0.691	0.595	0.652	0.521	0.553	0.532	0.514	0.482	0.519	0.495	0.475	0.471	0.554	0.505	0.512	0.507	0.522	0.519	0.526	0.441	0.477	0.542	0.394
	Draw 17	0.785	0.678	0.612	0.671	0.572	0.665	0.525	0.552	0.521	0.509	0.481	0.535	0.491	0.472	0.469	0.543	0.507	0.497	0.507	0.521	0.516	0.525	0.477	0.462	0.539	0.381
	Draw 18	0.765	0.685	0.603	0.675	0.603	0.659	0.512	0.561	0.524	0.510	0.475	0.530	0.491	0.469	0.450	0.548	0.497	0.507	0.505	0.525	0.519	0.520	0.439	0.476	0.536	0.401
	Draw 19	0.790	0.674	0.613	0.687	0.564	0.653	0.522	0.573	0.524	0.510	0.491	0.523	0.484	0.465	0.464	0.547	0.500	0.508	0.504	0.535	0.521	0.520	0.482	0.479	0.551	0.374
	Draw 20	0.778	0.668	0.619	0.660	0.600	0.634	0.532	0.550	0.519	0.514	0.493	0.526	0.483	0.473	0.461	0.547	0.500	0.501	0.502	0.522	0.519	0.527	0.467	0.476	0.541	0.407
DRAWS UP TO 300 VALVES	Draw 1	0.772	0.660	0.615	0.672	0.586	0.646	0.522	0.553	0.517	0.518	0.486	0.526	0.481	0.461	0.466	0.540	0.509	0.503	0.508	0.522	0.506	0.517	0.456	0.468	0.537	0.396
	Draw 2	0.775	0.663	0.615	0.663	0.573	0.662	0.527	0.565	0.528	0.501	0.483	0.533	0.482	0.479	0.458	0.537	0.506	0.502	0.510	0.530	0.506	0.516	0.500	0.468	0.546	0.396
	Draw 3	0.769	0.673	0.616	0.659	0.558	0.621	0.532	0.562	0.515	0.516	0.475	0.528	0.494	0.473	0.457	0.545	0.504	0.501	0.507	0.524	0.514	0.518	0.465	0.470	0.530	0.372
	Draw 4	0.771	0.672	0.607	0.669	0.593	0.660	0.516	0.570	0.522	0.513	0.489	0.529	0.495	0.471	0.459	0.535	0.510	0.504	0.511	0.524	0.506	0.518	0.492	0.463	0.546	0.400
	Draw 5	0.769	0.666	0.627	0.659	0.595	0.646	0.523	0.557	0.515	0.520	0.485	0.527	0.494	0.469	0.458	0.543	0.510	0.500	0.506	0.526	0.507	0.526	0.455	0.463	0.528	0.390
	Draw 6	0.772	0.671	0.609	0.660	0.583	0.671	0.532	0.554	0.530	0.512	0.493	0.527	0.490	0.468	0.452	0.542	0.507	0.499	0.513	0.526	0.506	0.522	0.474	0.472	0.543	0.386
	Draw 7	0.774	0.670	0.618	0.667	0.591	0.668	0.529	0.554	0.520	0.514	0.475	0.531	0.488	0.472	0.462	0.538	0.500	0.500	0.510	0.527	0.509	0.522	0.521	0.477	0.541	0.393
	Draw 8	0.765	0.666	0.616	0.668	0.567	0.653	0.527	0.560	0.528	0.524	0.490	0.527	0.494	0.472	0.475	0.548	0.507	0.501	0.507	0.531	0.509	0.525	0.481	0.465	0.536	0.400
	Draw 9	0.781	0.670	0.617	0.668	0.589	0.661	0.524	0.563	0.533	0.520	0.484	0.525	0.483	0.461	0.461	0.546	0.506	0.498	0.508	0.525	0.508	0.513	0.501	0.475	0.556	0.405
	Draw 10	0.780	0.673	0.617	0.669	0.594	0.643	0.528	0.560	0.534	0.516	0.474	0.528	0.498	0.470	0.456	0.534	0.504	0.501	0.511	0.528	0.514	0.509	0.483	0.473	0.530	0.388
	Draw 11	0.783	0.671	0.612	0.662	0.578	0.658	0.521	0.552	0.511	0.512	0.479	0.529	0.484	0.469	0.467	0.540	0.509	0.499	0.511	0.524	0.510	0.513	0.497	0.478	0.551	0.376
	Draw 12	0.777	0.673	0.611	0.655	0.581	0.636	0.514	0.549	0.530	0.515	0.474	0.522	0.491	0.476	0.461	0.544	0.500	0.501	0.514	0.526	0.505	0.521	0.476	0.466	0.536	0.387
	Draw 13	0.773	0.683	0.622	0.661	0.586	0.654	0.531	0.565	0.525	0.503	0.487	0.524	0.498	0.477	0.459	0.540	0.511	0.502	0.508	0.526	0.511	0.513	0.446	0.471	0.545	0.403
	Draw 14	0.778	0.663	0.628	0.664	0.582	0.643	0.519	0.563	0.519	0.530	0.485	0.530	0.487	0.476	0.460	0.536	0.510	0.500	0.513	0.528	0.512	0.514	0.458	0.468	0.534	0.396
	Draw 15	0.779	0.677	0.615	0.688	0.582	0.634	0.526	0.532	0.535	0.519	0.482	0.527	0.484	0.477	0.468	0.536	0.507	0.498	0.512	0.525	0.504	0.516	0.475	0.479	0.553	0.387
	Draw 16	0.788	0.661	0.621	0.663	0.589	0.666	0.519	0.563	0.528	0.515	0.483	0.519	0.486	0.469	0.456	0.546	0.500	0.500	0.510	0.527	0.510	0.518	0.449	0.462	0.539	0.383
	Draw 17	0.768	0.672	0.618	0.669	0.586	0.646	0.524	0.555	0.514	0.523	0.487	0.528	0.496	0.477	0.452	0.543	0.504	0.496	0.510	0.524	0.501	[illegible]	0.478	0.479	0.547	0.382
	Draw 18	0.775	0.662	0.611	0.658	0.573	0.647	0.515	0.574	0.520	0.516	0.480	0.527	0.487	0.469	0.454	0.540	0.494	0.496	0.516	0.526	0.513	0.511	0.483	0.479	0.543	0.392
	Draw 19	0.777	0.678	0.625	0.676	0.593	0.657	0.527	0.552	0.519	0.514	0.479	0.527	0.488	0.477	0.460	0.541	0.508	0.500	0.509	0.530	0.519	0.516	0.476	0.476	0.545	0.414
	Draw 20	0.768	0.656	0.614	0.687	0.595	0.656	0.527	0.542	0.526	0.510	0.477	0.525	0.494	0.474	0.456	0.549	0.499	0.500	0.502	0.524	0.503	0.520	0.488	0.472	0.532	0.378
DRAWS UP TO 350 VALVES	Draw 1	0.771	0.663	0.611	0.671	0.581	0.632	0.525	0.547	0.533	0.509	0.494	0.533	0.493	0.472	0.453	0.541	0.504	0.501	0.498	0.524	0.512	0.517	0.529	0.469	0.536	0.402
	Draw 2	0.772	0.665	0.621	0.681	0.571	0.651	0.524	0.563	0.520	0.520	0.493	0.532	0.491	0.475	0.456	0.542	0.505	0.499	0.494	0.526	0.508	0.518	0.531	0.468	0.539	0.389
	Draw 3	0.777	0.664	0.609	0.668	0.582	0.637	0.523	0.539	0.517	0.518	0.488	0.524	0.491	0.476	0.463	0.545	0.502	0.503	0.499	0.523	0.511	0.517	0.524	0.471	0.538	0.395
	Draw 4	0.766	0.666	0.609	0.664	0.589	0.649	0.522	0.544	0.514	0.514	0.478	0.526	0.491	0.469	0.457	0.543	0.503	0.491	0.509	0.527	0.512	0.519	0.533	0.474	0.548	0.392
	Draw 5	0.772	0.661	0.613	0.668	0.611	0.645	0.523	0.569	0.513	0.505	0.486	0.527	0.490	0.465	0.459	0.544	0.506	0.498	0.499	0.527	0.508	0.519	0.536	0.471	0.541	0.393
	Draw 6	0.768	0.667	0.615	0.669	0.585	0.650	0.526	0.542	0.530	0.511	0.482	0.531	0.491	0.470	0.461	0.538	0.504	0.497	0.503	0.529	0.516	0.520	0.528	0.471	0.542	0.387
	Draw 7	0.775	0.670	0.619	0.675	0.576	0.669	0.525	0.569	0.521	0.524	0.483	0.531	0.490	0.472	0.467	0.545	0.506	0.499	0.505	0.529	0.507	0.516	0.531	0.473	0.539	0.393
	Draw 8	0.767	0.659	0.618	0.681	0.567	0.649	0.524	0.548	0.513	0.508	0.485	0.527	0.493	0.461	0.456	0.543	0.508	0.497	0.504	0.527	0.506	0.519	0.524	0.473	0.550	0.387
	Draw 9	0.781	0.668	0.620	0.669	0.587	0.633	0.523	0.565	0.531	0.518	0.489	0.530	0.492	0.473	0.456	0.542	0.503	0.501	0.498	0.532	0.510	0.517	0.525	0.473	0.542	0.395
	Draw 10	0.774	0.675	0.631	0.668	0.605	0.648	0.524	0.554	0.513	0.513	0.475	0.536	0.491	0.456	0.457	0.545	0.507	0.498	0.501	0.522	0.510	0.516	0.525	0.474	0.557	0.396
	Draw 11	0.778	0.672	0.630	0.671	0.608	0.637	0.527	0.534	0.538	0.522	0.487	0.523	0.490	0.474	0.454	0.545	0.508	0.506	0.498	0.533	0.508	0.519	0.535	0.470	0.549	0.394
	Draw 12	0.767	0.666	0.615	0.654	0.582	0.651	0.521	0.558	0.540	0.495	0.483	0.532	0.490	0.477	0.452	0.542	0.505	0.504	0.499	0.528	0.507	0.520	0.529	0.472	0.541	0.401
	Draw 13	0.770	0.667	0.625	0.655	0.569	0.653	0.524	0.557	0.511	0.503	0.496	0.530	0.490	0.465	0.464	0.544	0.505	0.502	0.506	0.528	0.512	0.518	0.518	0.473	0.549	0.393
	Draw 14	0.777	0.669	0.591	0.669	0.603	0.639	0.527	0.579	0.542	0.498	0.484	0.526	0.491	0.460	0.456	0.544	0.506	0.502	0.504	0.534	0.512	0.517	0.531	0.473	0.549	0.389
	Draw 15	0.769	0.674	0.628	0.686	0.589	0.659	0.522	0.563	0.512	0.507	0.501	0.528	0.491	0.473	0.465	0.544	0.511	0.502	0.500	0.534	0.507	0.521	0.530	0.470	0.537	0.401
	Draw 16	0.768	0.674	0.619	0.698	0.588	0.645	0.527	0.560	0.529	0.510	0.471	0.528	0.491	0.481	0.450	0.543	0.502	0.495	0.500	0.528	0.511	0.519	0.529	0.473	0.534	0.392
	Draw 17	0.770	0.670	0.601	0.673	0.584	0.660	0.523	0.548	0.531	0.517	0.486	0.525	0.490	0.472	0.460	0.542	0.508	0.500	0.501	0.529	0.512	0.518	0.533	0.475	0.542	0.392
	Draw 18	0.772	0.663	0.645	0.672	0.570	0.631	0.525	0.563	0.528	0.506	0.497	0.529	0.490	0.489	0.465	0.545	0.507	0.500	0.503	0.529	0.509	0.524	0.525	0.472	0.540	0.392
	Draw 19	0.770	0.666	0.608	0.682	0.557	0.648	0.524	0.551	0.517	0.505	0.481	0.531	0.490	0.471	0.469	0.547	0.506	0.506	0.499	0.526	0.513	0.520	0.532	0.467	0.540	0.397
	Draw 20	0.771	0.668	0.636	0.692	0.573	0.649	0.527	0.577	0.525	0.524	0.489	0.524	0.491	0.459	0.450	0.544	0.506	0.498	0.505	0.527	0.508	0.516	0.525	0.473	0.542	0.387
DRAWS UP TO 400 VALVES	Draw 1	0.772	0.669	0.596	0.652	0.605	0.649	0.521	0.545	0.519	0.505	0.491	0.533	0.487	0.466	0.457	0.550	0.503	0.496	0.503	0.521	0.519	0.519	0.521	0.473	0.542	0.394
	Draw 2	0.773	0.668	0.610	0.664	0.579	0.647	0.523	0.558	0.518	0.507	0.485	0.536	0.479	0.463	0.457	0.541	0.507	0.494	0.497	0.524	0.504	0.525	0.530	0.474	0.542	0.386
	Draw 3	0.773	0.672	0.617	0.666	0.585	0.653	0.526	0.561	0.533	0.518	0.465	0.528	0.489	0.476	0.463	0.544	0.500	0.498	0.496	0.524	0.507	0.524	0.530	0.474	0.542	0.389
	Draw 4	0.772	0.670	0.612	0.663	0.597	0.644	0.527	0.538	0.539	0.514	0.482	0.531	0.485	0.468	0.457	0.543	0.505	0.500	0.491	0.533	0.501	0.521	0.533	0.476	0.541	0.394
	Draw 5	0.773	0.667	0.613	0.659	0.585	0.645	0.516	0.571	0.523	0.505	0.497	0.524	0.496	0.466	0.457	0.541	0.506	0.495	0.506	0.521	0.513	0.516	0.532	0.471	0.541	0.387
	Draw 6	0.770	0.666	0.608	0.655	0.592	0.646	0.524	0.558	0.513	0.515	0.477	0.525	0.496	0.469	0.463	0.540	0.507	0.503	0.496	0.524	0.509	0.519	0.524	0.472	0.542	0.398
	Draw 7	0.775	0.670	0.630	0.672	0.586	0.652	0.525	0.539	0.533	0.519	0.477	0.525	0.488	0.471	0.456	0.542	0.507	0.500	0.497	0.532	0.510	0.515	0.526	0.471	0.540	0.386
	Draw 8	0.773	0.668	0.612	0.667	0.588	0.637	0.529	0.545	0.515	0.519	0.492	0.535	0.490	0.484	0.461	0.547	0.493	0.498	0.504	0.525	0.507	0.517	0.527	0.471	0.540	0.389
	Draw 9	0.771	0.666	0.613	0.658	0.590	0.642	0.523	0.560	0.539	0.516	0.492	0.529	0.488	0.480	0.459	0.547	0.504	0.499	0.505	0.525	0.509	0.525	0.531	0.474	0.539	0.398
	Draw 10	0.772	0.669	0.626	0.676	0.589	0.633	0.520	0.569	0.536	0.513	0.484	0.518	0.492	0.462	0.458	0.544	0.511	0.504	0.490	0.520	0.508	0.520	0.531	0.473	0.544	0.385
	Draw 11	0.771	0.668	0.627	0.657	0.600	0.657	0.520	0.546	0.522	0.526	0.493	0.531	0.490	0.470	0.451	0.541	0.506	0.511	0.514	0.527	0.505	0.515	0.527	0.471	0.541	0.388
	Draw 12	0.773	0.667	0.631	0.668	0.602	0.651	0.523	0.550	0.524	0.508	0.483	0.530	0.497	0.466	0.467	0.548	0.500	0.491	0.487	0.524	0.506	0.514	0.530	0.474	0.541	0.395
	Draw 13	[illegible]	[illegible]	[illegible]	[illegible]	[illegible]	[illegible]	[illegible]	[illegible]	[illegible]	[illegible]	[illegible]	[illegible]	[illegible]	[illegible]	[illegible]	[illegible]	[illegible]	[illegible]	[illegible]	[illegible]	[illegible]	[illegible]	[illegible]	[illegible]	[illegible]	[illegible]
	Draw 14	[illegible]	[illegible]	[illegible]	[illegible]	[illegible]	[illegible]	[illegible]	[illegible]	[illegible]	[illegible]	[illegible]	[illegible]	[illegible]	[illegible]	[illegible]	[illegible]	[illegible]	[illegible]	[illegible]	[illegible]	[illegible]	[illegible]	[illegible]	[illegible]	[illegible]	[illegible]
	Draw 15	[illegible]	[illegible]	[illegible]	[illegible]	[illegible]	[illegible]	[illegible]	[illegible]	[illegible]	[illegible]	[illegible]	[illegible]	[illegible]	[illegible]	[illegible]	[illegible]	[illegible]	[illegible]	[illegible]	[illegible]	[illegible]	[illegible]	[illegible]	[illegible]	[illegible]	[illegible]
	Draw 16	[illegible]	[illegible]	[illegible]	[illegible]	[illegible]	[illegible]	[illegible]	[illegible]	[illegible]	[illegible]	[illegible]	[illegible]	[illegible]	[illegible]	[illegible]	[illegible]	[illegible]	[illegible]	[illegible]	[illegible]	[illegible]	[illegible]	[illegible]	[illegible]	[illegible]	[illegible]
	Draw 17	[illegible]	[illegible]	[illegible]	[illegible]	[illegible]	[illegible]	[illegible]	[illegible]	[illegible]	[illegible]	[illegible]	[illegible]	[illegible]	[illegible]	[illegible]	[illegible]	[illegible]	[illegible]	[illegible]	[illegible]	[illegible]	[illegible]	[illegible]	[illegible]	[illegible]	[illegible]
	Draw 18	[illegible]	[illegible]	[illegible]	[illegible]	[illegible]	[illegible]	[illegible]	[illegible]	[illegible]	[illegible]	[illegible]	[illegible]	[illegible]	[illegible]	[illegible]	[illegible]	[illegible]	[illegible]	[illegible]	[illegible]	[illegible]	[illegible]	[illegible]	[illegible]	[illegible]	[illegible]
	Draw 19	[illegible]	[illegible]	[illegible]	[illegible]	[illegible]	[illegible]	[illegible]	[illegible]	[illegible]	[illegible]	[illegible]	[illegible]	[illegible]	[illegible]	[illegible]	[illegible]	[illegible]	[illegible]	[illegible]	[illegible]	[illegible]	[illegible]	[illegible]	[illegible]	[illegible]	[illegible]
	Draw 20	[illegible]	[illegible]	[illegible]	[illegible]	[illegible]	[illegible]	[illegible]	[illegible]	[illegible]	[illegible]	[illegible]	[illegible]	[illegible]	[illegible]	[illegible]	[illegible]	[illegible]	[illegible]	[illegible]	[illegible]	[illegible]	[illegible]	[illegible]	[illegible]	[illegible]	[illegible]

RIVER DRAWA

		D1	D2	D3	D4	D5	D6	D7	D8	D9	D10	D11	D12	D13
DRAWS UP TO 50 VALVES	Draw 1	0.843	0.645	0.701	0.762	0.614	0.540	0.656	0.573	0.606	0.570	0.596	0.519	0.533
	Draw 2	0.867	0.667	0.777	0.760	0.623	0.480	0.685	0.554	0.533	0.556	0.580	0.565	0.503
	Draw 3	0.849	0.648	0.741	0.766	0.622	0.465	0.613	0.622	0.617	0.560	0.604	0.603	0.589
	Draw 4	0.886	0.636	0.736	0.766	0.656	0.536	0.645	0.574	0.597	0.533	0.658	0.537	0.501
	Draw 5	0.850	0.644	0.712	0.755	0.564	0.459	0.635	0.570	0.582	0.588	0.614	0.593	0.518
	Draw 6	0.861	0.653	0.740	0.727	0.590	0.499	0.587	0.551	0.599	0.549	0.616	0.600	0.508
	Draw 7	0.879	0.610	0.692	0.753	0.636	0.515	0.611	0.582	0.621	0.548	0.609	0.595	0.578
	Draw 8	0.838	0.619	0.702	0.747	0.646	0.461	0.671	0.536	0.557	0.553	0.583	0.543	0.490
	Draw 9	0.872	0.664	0.714	0.743	0.606	0.600	0.682	0.557	0.633	0.520	0.588	0.584	0.512
	Draw 10	0.876	0.655	0.719	0.738	0.583	0.504	0.610	0.567	0.603	0.606	0.600	0.595	0.471
	Draw 11	0.868	0.686	0.787	0.738	0.636	0.393	0.642	0.543	0.533	0.573	0.578	0.518	0.498
	Draw 12	0.839	0.679	0.670	0.763	0.585	0.522	0.569	0.593	0.580	0.558	0.669	0.540	0.460
	Draw 13	0.867	0.639	0.701	0.787	0.601	0.515	0.627	0.551	0.559	0.530	0.566	0.578	0.543
	Draw 14	0.862	0.653	0.694	0.721	0.622	0.507	0.619	0.568	0.633	0.547	0.590	0.542	0.485
	Draw 15	0.849	0.644	0.733	0.706	0.593	0.491	0.664	0.585	0.599	0.536	0.597	0.566	0.511
	Draw 16	0.880	0.629	0.686	0.753	0.587	0.452	0.663	0.535	0.577	0.564	0.609	0.553	0.516
	Draw 17	0.853	0.649	0.737	0.710	0.637	0.505	0.544	0.558	0.568	0.544	0.568	0.580	0.562
	Draw 18	0.800	0.675	0.670	0.747	0.629	0.458	0.621	0.559	0.589	0.556	0.554	0.567	0.467
	Draw 19	0.856	0.638	0.699	0.729	0.596	0.531	0.640	0.570	0.567	0.545	0.612	0.554	0.485
	Draw 20	0.823	0.645	0.660	0.732	0.627	0.530	0.605	0.517	0.594	0.587	0.596	0.561	0.480
DRAWS UP TO 100 VALVES	Draw 1	0.844	0.647	0.696	0.726	0.625	0.502	0.614	0.564	0.558	0.583	0.581	0.535	0.488
	Draw 2	0.864	0.637	0.697	0.742	0.628	0.493	0.631	0.542	0.577	0.554	0.569	0.593	0.512
	Draw 3	0.867	0.644	0.709	0.773	0.617	0.511	0.607	0.545	0.592	0.535	0.588	0.589	0.495
	Draw 4	0.829	0.669	0.714	0.753	0.608	0.489	0.615	0.570	0.609	0.563	0.598	0.570	0.498
	Draw 5	0.870	0.665	0.694	0.748	0.624	0.524	0.647	0.540	0.621	0.541	0.571	0.560	0.534
	Draw 6	0.851	0.638	0.713	0.763	0.596	0.517	0.637	0.547	0.579	0.532	0.577	0.594	0.505
	Draw 7	0.856	0.635	0.704	0.749	0.599	0.459	0.614	0.542	0.583	0.540	0.565	0.583	0.520
	Draw 8	0.867	0.655	0.732	0.737	0.588	0.515	0.621	0.585	0.603	0.538	0.629	0.613	0.485
	Draw 9	0.823	0.643	0.719	0.742	0.614	0.491	0.653	0.510	0.609	0.566	0.574	0.551	0.482
	Draw 10	0.849	0.657	0.702	0.707	0.623	0.557	0.589	0.549	0.595	0.574	0.566	0.557	0.497
	Draw 11	0.864	0.657	0.712	0.727	0.628	0.516	0.606	0.563	0.604	0.522	0.613	0.578	0.553
	Draw 12	0.861	0.662	0.688	0.768	0.608	0.505	0.611	0.572	0.596	0.548	0.610	0.569	0.524
	Draw 13	0.843	0.651	0.715	0.754	0.601	0.514	0.649	0.567	0.583	0.572	0.601	0.586	0.518
	Draw 14	0.868	0.673	0.708	0.734	0.612	0.513	0.595	0.555	0.582	0.554	0.595	0.555	0.485
	Draw 15	0.840	0.647	0.677	0.732	0.623	0.487	0.620	0.552	0.607	0.555	0.613	0.548	0.508
	Draw 16	0.853	0.621	0.701	0.756	0.617	0.562	0.627	0.546	0.598	0.557	0.574	0.511	0.509
	Draw 17	0.852	0.636	0.692	0.747	0.631	0.508	0.591	0.571	0.587	0.551	0.573	0.563	0.514
	Draw 18	0.840	0.639	0.703	0.731	0.627	0.513	0.638	0.529	0.587	0.594	0.597	0.545	0.475
	Draw 19	0.846	0.626	0.690	0.739	0.616	0.522	0.639	0.563	0.593	0.538	0.631	0.583	0.506
	Draw 20	0.862	0.643	0.733	0.783	0.589	0.497	0.671	0.542	0.545	0.512	0.575	0.563	0.546
DRAWS UP TO 150 VALVES	Draw 1	0.848	0.644	0.710	0.755	0.608	0.497	0.600	0.553	0.571	0.550	0.584	0.572	0.511
	Draw 2	0.845	0.647	0.718	0.771	0.616	0.490	0.605	0.547	0.566	0.534	0.583	0.579	0.514
	Draw 3	0.859	0.661	0.705	0.749	0.622	0.489	0.643	0.573	0.595	0.551	0.590	0.602	0.509
	Draw 4	0.856	0.654	0.700	0.742	0.604	0.492	0.627	0.560	0.588	0.557	0.603	0.542	0.514
	Draw 5	0.861	0.663	0.693	0.745	0.613	0.497	0.620	0.562	0.603	0.534	0.612	0.574	0.511
	Draw 6	0.846	0.674	0.678	0.751	0.617	0.471	0.644	0.543	0.595	0.583	0.591	0.570	0.497
	Draw 7	0.833	0.656	0.684	0.748	0.604	0.508	0.626	0.549	0.569	0.570	0.590	0.579	0.540
	Draw 8	0.867	0.647	0.705	0.746	0.626	0.529	0.611	0.546	0.569	0.565	0.596	0.584	0.479
	Draw 9	0.846	0.633	0.720	0.767	0.619	0.524	0.620	0.559	0.588	0.572	0.606	0.575	0.497
	Draw 10	0.823	0.649	0.700	0.741	0.607	0.496	0.635	0.550	0.587	0.551	0.579	0.561	0.496
	Draw 11	0.851	0.633	0.703	0.725	0.637	0.500	0.567	0.551	0.581	0.529	0.600	0.566	0.514
	Draw 12	0.855	0.646	0.690	0.735	0.625	0.487	0.652	0.534	0.575	0.539	0.586	0.568	0.511
	Draw 13	0.853	0.646	0.701	0.766	0.603	0.494	0.622	0.547	0.597	0.550	0.604	0.561	0.508
	Draw 14	0.853	0.656	0.707	0.745	0.624	0.498	0.631	0.544	0.576	0.551	0.595	0.600	0.502
	Draw 15	0.870	0.653	0.686	0.768	0.623	0.491	0.616	0.557	0.574	0.566	0.600	0.574	0.498
	Draw 16	0.868	0.640	0.694	0.734	0.633	0.473	0.611	0.540	0.579	0.569	0.569	0.572	0.504
	Draw 17	0.851	0.648	0.686	0.734	0.625	0.509	0.637	0.550	0.584	0.549	0.590	0.559	0.508
	Draw 18	0.811	0.646	0.686	0.742	0.600	0.517	0.651	0.563	0.572	0.552	0.601	0.558	0.545
	Draw 19	0.864	0.647	0.706	0.730	0.605	0.495	0.630	0.571	0.589	0.570	0.589	0.565	0.516
	Draw 20	0.861	0.648	0.699	0.748	0.618	0.507	0.634	0.548	0.573	0.549	0.600	0.567	0.522
DRAWS UP TO 200 VALVES	Draw 1	0.850	0.660	0.704	0.739	0.606	0.505	0.637	0.566	0.575	0.544	0.598	0.567	0.512
	Draw 2	0.847	0.646	0.685	0.750	0.617	0.494	0.623	0.538	0.610	0.558	0.613	0.574	0.489
	Draw 3	0.846	0.651	0.684	0.729	0.626	0.520	0.634	0.551	0.564	0.550	0.588	0.562	0.521
	Draw 4	0.852	0.628	0.717	0.742	0.604	0.505	0.621	0.544	0.581	0.565	0.615	0.562	0.491
	Draw 5	0.848	0.652	0.700	0.750	0.621	0.472	0.628	0.556	0.594	0.554	0.606	0.560	0.512
	Draw 6	0.863	0.654	0.700	0.769	0.649	0.501	0.606	0.539	0.587	0.543	0.592	0.561	0.508
	Draw 7	0.840	0.653	0.684	0.744	0.613	0.503	0.641	0.563	0.577	0.557	0.598	0.563	0.494
	Draw 8	0.844	0.659	0.693	0.734	0.619	0.477	0.597	0.555	0.594	0.564	0.593	0.555	0.527
	Draw 9	0.854	0.639	0.689	0.743	0.618	0.508	0.633	0.556	0.583	0.556	0.597	0.569	0.513
	Draw 10	0.849	0.654	0.705	0.748	0.624	0.492	0.620	0.553	0.588	0.550	0.599	0.550	0.518
	Draw 11	0.841	0.644	0.708	0.750	0.606	0.499	0.618	0.544	0.584	0.547	0.597	0.558	0.505
	Draw 12	0.852	0.658	0.697	0.751	0.635	0.504	0.631	0.546	0.586	0.579	0.593	0.578	0.514
	Draw 13	0.863	0.661	0.690	0.752	0.618	0.494	0.623	0.544	0.585	0.554	0.607	0.557	0.522
	Draw 14	0.857	0.656	0.696	0.735	0.623	0.497	0.619	0.557	0.580	0.546	0.606	0.566	0.518
	Draw 15	0.856	0.654	0.700	0.743	0.600	0.495	0.621	0.557	0.596	0.547	0.600	0.582	0.501
	Draw 16	0.839	0.668	0.690	0.748	0.614	0.502	0.643	0.559	0.587	0.549	0.589	0.596	0.512
	Draw 17	0.857	0.662	0.697	0.719	0.618	0.518	0.622	0.543	0.573	0.551	0.585	0.564	0.489
	Draw 18	0.849	0.658	0.683	0.739	0.626	0.490	0.641	0.553	0.592	0.555	0.589	0.568	0.498
	Draw 19	0.864	0.657	0.708	0.745	0.614	0.500	0.637	0.558	0.571	0.560	0.595	0.579	0.492
	Draw 20	0.843	0.640	0.702	0.750	0.627	0.481	0.619	0.559	0.560	0.534	0.595	0.559	0.503
DRAWS UP TO 250 VALVES	Draw 1	0.864	0.657	0.696	0.753	0.607	0.500	0.626	0.546	0.587	0.560	0.590	0.555	0.514
	Draw 2	0.857	0.656	0.698	0.745	0.602	0.515	0.628	0.551	0.577	0.553	0.602	0.559	0.508
	Draw 3	0.859	0.653	0.706	0.736	0.614	0.511	0.628	0.553	0.577	0.552	0.604	0.542	0.511
	Draw 4	0.855	0.659	0.709	0.734	0.608	0.487	0.631	0.552	0.578	0.555	0.598	0.552	0.521
	Draw 5	0.861	0.654	0.689	0.743	0.607	0.521	0.623	0.554	0.584	0.557	0.586	0.566	0.526
	Draw 6	0.841	0.649	0.686	0.738	0.622	0.515	0.628	0.551	0.584	0.558	0.591	0.576	0.483
	Draw 7	0.848	0.643	0.706	0.736	0.612	0.485	0.621	0.552	0.588	0.554	0.583	0.566	0.501
	Draw 8	0.862	0.650	0.696	0.723	0.620	0.500	0.630	0.554	0.588	0.565	0.594	0.563	0.498
	Draw 9	0.852	0.651	0.689	0.742	0.617	0.486	0.627	0.552	0.582	0.555	0.577	0.560	0.506
	Draw 10	0.842	0.648	0.695	0.742	0.621	0.491	0.620	0.567	0.580	0.555	0.597	0.547	0.503
	Draw 11	0.853	0.657	0.697	0.757	0.602	0.495	0.618	0.557	0.583	0.555	0.591	0.571	0.515
	Draw 12	0.853	0.656	0.690	0.755	0.615	0.530	0.618	0.560	0.583	0.543	0.582	0.564	0.515
	Draw 13	0.856	0.661	0.703	0.747	0.615	0.495	0.615	0.553	0.586	0.549	0.592	0.567	0.520
	Draw 14	0.865	0.639	0.703	0.755	0.625	0.517	0.639	0.557	0.586	0.540	0.586	0.569	0.510
	Draw 15	0.853	0.650	0.699	0.750	0.617	0.492	0.619	0.550	0.579	0.563	0.606	0.576	0.514
	Draw 16	0.856	0.652	0.694	0.746	0.619	0.492	0.618	0.551	0.579	0.549	0.597	0.566	0.498
	Draw 17	0.859	0.642	0.686	0.752	0.621	0.494	0.626	0.546	0.588	0.543	0.598	0.563	0.503
	Draw 18	0.855	0.658	0.707	0.750	0.616	0.489	0.628	0.556	0.584	0.549	0.603	0.563	0.512
	Draw 19	0.845	0.648	0.691	0.756	0.616	0.483	0.626	0.543	0.581	0.542	0.592	0.570	0.517
	Draw 20	0.853	0.648	0.688	0.741	0.630	0.498	0.638	0.549	0.579	0.555	0.604	0.554	0.502
DRAWS UP TO 300 VALVES	Draw 1	0.858	0.656	0.697	0.737	0.614	0.496	0.623	0.552	0.588	0.550	0.596	0.582	0.501
	Draw 2	0.859	0.651	0.686	0.737	0.608	0.504	0.629	0.553	0.590	0.555	0.591	0.572	0.506
	Draw 3	0.846	0.652	0.695	0.743	0.606	0.481	0.632	0.554	0.584	0.547	0.596	0.570	0.521
	Draw 4	0.849	0.653	0.705	0.741	0.615	0.490	0.629	0.555	0.585	0.553	0.593	0.572	0.503
	Draw 5	0.854	0.658	0.699	0.750	0.612	0.491	0.628	0.554	0.583	0.555	0.600	0.562	0.518
	Draw 6	0.849	0.653	0.692	0.745	0.602	0.497	0.622	0.552	0.588	0.554	0.586	0.564	0.496
	Draw 7	0.849	0.642	0.676	0.750	0.618	0.519	0.618	0.553	0.594	0.554	0.597	0.570	0.513
	Draw 8	0.862	0.645	0.681	0.754	0.614	0.512	0.638	0.554	0.581	0.559	0.589	0.567	0.506
	Draw 9	0.849	0.645	0.699	0.737	0.624	0.514	0.616	0.553	0.583	0.554	0.591	0.561	0.517
	Draw 10	0.861	0.651	0.688	0.736	0.614	0.507	0.635	0.552	0.584	0.553	0.595	0.561	0.517
	Draw 11	0.849	0.656	0.677	0.741	0.609	0.506	0.621	0.555	0.584	0.565	0.593	0.562	0.508
	Draw 12	0.857	0.653	0.702	0.743	0.620	0.503	0.619	0.552	0.583	0.548	0.597	0.566	0.518
	Draw 13	0.857	0.644	0.688	0.744	0.607	0.514	0.625	0.551	0.588	0.551	0.600	0.560	0.506
	Draw 14	0.849	0.648	0.687	0.743	0.620	0.503	0.635	0.552	0.576	0.558	0.593	0.567	0.504
	Draw 15	0.855	0.650	0.693	0.745	0.619	0.492	0.627	0.551	0.585	0.555	0.591	0.560	0.507
	Draw 16	0.852	0.657	0.690	0.731	0.618	0.500	0.625	0.551	0.585	0.554	0.600	0.570	0.505
	Draw 17	0.844	0.653	0.681	0.749	0.614	0.502	0.627	0.553	0.581	0.546	0.595	0.572	0.518
	Draw 18	0.851	0.652	0.681	0.736	0.625	0.511	0.631	0.555	0.575	0.548	0.593	0.575	0.500
	Draw 19	0.857	0.662	0.685	0.746	0.619	0.497	0.634	0.552	0.581	0.550	0.588	0.558	0.502
	Draw 20	0.853	0.656	0.697	0.745	0.615	0.500	0.631	0.551	0.585	0.545	0.593	0.562	0.503
DRAWS UP TO 350 VALVES	Draw 1	0.856	0.650	0.703	0.735	0.622	0.500	0.618	0.544	0.583	0.549	0.600	0.570	0.514
	Draw 2	0.856	0.650	0.696	0.746	0.612	0.497	0.615	0.556	0.583	0.554	0.591	0.566	0.505
	Draw 3	0.852	0.653	0.700	0.748	0.609	0.507	0.624	0.539	0.580	0.556	0.591	0.569	0.517
	Draw 4	0.853	0.649	0.700	0.744	0.621	0.510	0.627	0.563	0.581	0.554	0.580	0.561	0.509
	Draw 5	0.852	0.658	0.702	0.742	0.613	0.503	0.629	0.555	0.583	0.549	0.593	0.569	0.508
	Draw 6	0.853	0.654	0.691	0.748	0.603	0.498	0.620	0.556	0.584	0.552	0.591	0.565	0.505
	Draw 7	0.855	0.650	0.692	0.740	0.615	0.503	0.622	0.553	0.582	0.553	0.597	0.566	0.511
	Draw 8	0.851	0.654	0.694	0.742	0.620	0.496	0.625	0.544	0.583	0.548	0.595	0.569	0.508
	Draw 9	0.852	0.652	0.693	0.744	0.622	0.501	0.621	0.546	0.585	0.556	0.584	0.567	0.513
	Draw 10	0.857	0.654	0.699	0.741	0.611	0.510	0.610	0.544	0.586	0.554	0.591	0.563	0.517
	Draw 11	0.853	0.648	0.697	0.739	0.609	0.497	0.625	0.557	0.587	0.553	0.589	0.564	0.509
	Draw 12	0.854	0.648	0.697	0.750	0.613	0.506	0.632	0.557	0.582	0.553	0.592	0.569	0.511
	Draw 13	0.855	0.650	0.697	0.753	0.612	0.508	0.619	0.559	0.582	0.550	0.591	0.566	0.510
	Draw 14	0.854	0.653	0.702	0.737	0.611	0.491	0.628	0.543	0.583	0.553	0.592	0.573	0.510
	Draw 15	0.855	0.656	0.697	0.746	0.618	0.500	0.615	0.553	0.584	0.553	0.595	0.564	0.515
	Draw 16	0.855	0.649	0.704	0.743	0.605	0.507	0.629	0.558	0.585	0.553	0.598	0.571	0.511
	Draw 17	0.849	0.649	0.699	0.743	0.620	0.507	0.627	0.559	0.586	0.550	0.596	0.572	0.511
	Draw 18	0.851	0.654	0.701	0.742	0.613	0.497	0.628	0.546	0.588	0.552	0.593	0.570	0.509
	Draw 19	0.855	0.650	0.691	0.748	0.608	0.498	0.622	0.551	0.584	0.550	0.595	0.569	0.513
	Draw 20	0.855	0.646	0.687	0.742	0.621	0.508	0.623	0.553	0.580	0.552	0.592	0.567	0.508
DRAWS UP TO 400 VALVES	Draw 1	0.854	0.655	0.694	0.746	0.614	0.505	0.626	0.553	0.576	0.553	0.597	0.567	0.510
	Draw 2	0.854	0.654	0.697	0.747	0.612	0.499	0.627	0.557	0.577	0.558	0.598	0.569	0.509
	Draw 3	0.855	0.650	0.701	0.741	0.614	0.502	0.627	0.550	0.579	0.556	0.592	0.567	0.505
	Draw 4	0.855	0.651	0.703	0.746	0.615	0.509	0.629	0.551	0.584	0.554	0.590	0.566	0.509
	Draw 5	0.852	0.651	0.692	0.738	0.614	0.504	0.626	0.546	0.574	0.550	0.593	0.568	0.516
	Draw 6	0.852	0.655	0.691	0.740	0.611	0.503	0.627	0.556	0.575	0.559	0.590	0.567	0.514
	Draw 7	0.851	0.651	0.691	0.739	0.614	0.508	0.618	0.543	0.584	0.554	0.593	0.563	0.508
	Draw 8	0.855	0.655	0.692	0.742	0.613	0.503	0.623	0.549	0.586	0.552	0.596	0.566	0.510
	Draw 9	0.853	0.647	0.695	0.745	0.614	0.506	0.627	0.552	0.584	0.552	0.593	0.570	0.511
	Draw 10	0.852	0.649	0.700	0.746	0.612	0.502	0.625	0.553	0.582	0.543	0.592	0.567	0.515
	Draw 11	0.853	0.650	0.702	0.745	0.621	0.505	0.625	0.554	0.578	0.556	0.594	0.564	0.509
	Draw 12	0.853	0.644	0.689	0.745	0.613	0.498	0.624	0.555	0.588	0.555	0.594	0.563	0.506
	Draw 13	[illegible]	[illegible]	[illegible]	[illegible]	[illegible]	[illegible]	[illegible]	[illegible]	[illegible]	[illegible]	[illegible]	[illegible]	[illegible]
	Draw 14	[illegible]	[illegible]	[illegible]	[illegible]	[illegible]	[illegible]	[illegible]	[illegible]	[illegible]	[illegible]	[illegible]	[illegible]	[illegible]
	Draw 15	[illegible]	[illegible]	[illegible]	[illegible]	[illegible]	[illegible]	[illegible]	[illegible]	[illegible]	[illegible]	[illegible]	[illegible]	[illegible]
	Draw 16	[illegible]	[illegible]	[illegible]	[illegible]	[illegible]	[illegible]	[illegible]	[illegible]	[illegible]	[illegible]	[illegible]	[illegible]	[illegible]
	Draw 17	[illegible]	[illegible]	[illegible]	[illegible]	[illegible]	[illegible]	[illegible]	[illegible]	[illegible]	[illegible]	[illegible]	[illegible]	[illegible]
	Draw 18	[illegible]	[illegible]	[illegible]	[illegible]	[illegible]	[illegible]	[illegible]	[illegible]	[illegible]	[illegible]	[illegible]	[illegible]	[illegible]
	Draw 19	[illegible]	[illegible]	[illegible]	[illegible]	[illegible]	[illegible]	[illegible]	[illegible]	[illegible]	[illegible]	[illegible]	[illegible]	[illegible]
	Draw 20	[illegible]	[illegible]	[illegible]	[illegible]	[illegible]	[illegible]	[illegible]	[illegible]	[illegible]	[illegible]	[illegible]	[illegible]	[illegible]

Supplementary Table F Results of drew values (20 x each counting sum) of Polish Diatom Index for lakes (IOJ) for both river systems

		RIVER INA		RIVER DRAWA												
		**JK**	**JB**	**JD1**	**JD2**	**JD3**	**JD4**	**JD5**	**JD6**	**JD7**	**JD8**	**JD9**	**JD10**	**JD11**	**JD12**	**JD13**
**UP TO 50 VALVES**	**Draw 1**	0.556	0.557	0.780	0.480	0.431	0.494	0.585	0.403	0.638	0.722	0.697	0.535	0.596	0.604	0.500
	**Draw 2**	0.623	0.624	0.728	0.505	0.490	0.493	0.653	0.410	0.604	0.671	0.679	0.505	0.531	0.600	0.523
	**Draw 3**	0.636	0.637	0.758	0.522	0.481	0.533	0.563	0.427	0.648	0.670	0.634	0.488	0.560	0.605	0.478
	**Draw 4**	0.635	0.635	0.757	0.546	0.463	0.486	0.633	0.397	0.582	0.729	0.692	0.516	0.567	0.619	0.499
	**Draw 5**	0.588	0.588	0.757	0.451	0.467	0.528	0.599	0.394	0.574	0.758	0.695	0.500	0.571	0.636	0.462
	**Draw 6**	0.560	0.561	0.652	0.531	0.500	0.521	0.552	0.393	0.656	0.707	0.613	0.580	0.579	0.629	0.534
	**Draw 7**	0.624	0.624	0.733	0.493	0.498	0.521	0.560	0.399	0.573	0.732	0.614	0.543	0.565	0.624	0.457
	**Draw 8**	0.629	0.630	0.709	0.490	0.478	0.544	0.589	0.406	0.600	0.738	0.709	0.570	0.594	0.607	0.508
	**Draw 9**	0.718	0.718	0.745	0.461	0.478	0.493	0.641	0.417	0.565	0.745	0.652	0.489	0.581	0.643	0.523
	**Draw 10**	0.566	0.566	0.670	0.498	0.465	0.492	0.528	0.401	0.624	0.746	0.643	0.443	0.545	0.699	0.522
	**Draw 11**	0.551	0.551	0.707	0.450	0.467	0.470	0.617	0.417	0.580	0.695	0.593	0.447	0.544	0.668	0.471
	**Draw 12**	0.659	0.659	0.707	0.527	0.457	0.487	0.602	0.428	0.582	0.699	0.640	0.543	0.604	0.655	0.475
	**Draw 13**	0.601	0.601	0.733	0.483	0.482	0.461	0.589	0.393	0.622	0.767	0.642	0.459	0.548	0.601	0.468
	**Draw 14**	0.606	0.606	0.732	0.458	0.472	0.527	0.591	0.432	0.651	0.722	0.694	0.502	0.629	0.672	0.484
	**Draw 15**	0.644	0.645	0.703	0.492	0.458	0.483	0.589	0.393	0.560	0.658	0.642	0.509	0.540	0.645	0.475
	**Draw 16**	0.619	0.619	0.757	0.476	0.522	0.596	0.625	0.407	0.618	0.761	0.660	0.535	0.528	0.661	0.450
	**Draw 17**	0.675	0.675	0.687	0.510	0.535	0.510	0.619	0.421	0.598	0.762	0.685	0.527	0.590	0.627	0.465
	**Draw 18**	0.603	0.603	0.725	0.548	0.542	0.517	0.593	0.439	0.607	0.774	0.609	0.524	0.585	0.681	0.469
	**Draw 19**	0.618	0.618	0.730	0.459	0.452	0.583	0.551	0.406	0.582	0.711	0.641	0.458	0.525	0.688	0.507
	**Draw 20**	0.613	0.614	0.757	0.505	0.531	0.545	0.577	0.407	0.626	0.733	0.627	0.437	0.570	0.647	0.498
**DRAWS UP TO 100 VALVES**	**Draw 1**	0.621	0.621	0.738	0.485	0.473	0.485	0.550	0.411	0.619	0.727	0.699	0.512	0.561	0.660	0.502
	**Draw 2**	0.631	0.632	0.756	0.512	0.465	0.534	0.560	0.400	0.617	0.756	0.653	0.544	0.551	0.622	0.458
	**Draw 3**	0.653	0.653	0.741	0.481	0.465	0.509	0.614	0.406	0.619	0.739	0.698	0.506	0.542	0.622	0.501
	**Draw 4**	0.566	0.566	0.705	0.499	0.495	0.481	0.592	0.410	0.606	0.724	0.623	0.490	0.550	0.689	0.480
	**Draw 5**	0.642	0.642	0.729	0.487	0.454	0.515	0.562	0.417	0.622	0.726	0.637	0.527	0.580	0.647	0.517
	**Draw 6**	0.607	0.608	0.699	0.503	0.523	0.488	0.617	0.405	0.611	0.680	0.630	0.556	0.580	0.629	0.498
	**Draw 7**	0.600	0.600	0.702	0.493	0.520	0.546	0.582	0.410	0.609	0.703	0.673	0.499	0.575	0.669	0.505
	**Draw 8**	0.638	0.639	0.746	0.519	0.514	0.515	0.570	0.401	0.573	0.737	0.664	0.505	0.548	0.668	0.494
	**Draw 9**	0.632	0.632	0.705	0.508	0.475	0.535	0.586	0.412	0.601	0.733	0.694	0.512	0.563	0.599	0.498
	**Draw 10**	0.564	0.565	0.715	0.500	0.524	0.494	0.577	0.411	0.587	0.688	0.702	0.488	0.565	0.626	0.477
	**Draw 11**	0.637	0.637	0.684	0.480	0.524	0.500	0.589	0.410	0.645	0.755	0.644	0.541	0.545	0.690	0.500
	**Draw 12**	0.606	0.606	0.727	0.498	0.476	0.491	0.596	0.410	0.612	0.681	0.615	0.545	0.562	0.681	0.496
	**Draw 13**	0.637	0.638	0.709	0.483	0.537	0.526	0.581	0.405	0.588	0.771	0.634	0.538	0.557	0.614	0.498
	**Draw 14**	0.638	0.638	0.696	0.499	0.500	0.516	0.581	0.416	0.617	0.727	0.678	0.523	0.559	0.609	0.518
	**Draw 15**	0.591	0.591	0.711	0.499	0.493	0.506	0.634	0.412	0.598	0.746	0.629	0.470	0.567	0.630	0.485
	**Draw 16**	0.662	0.662	0.704	0.509	0.521	0.531	0.603	0.400	0.600	0.731	0.571	0.502	0.541	0.677	0.508
	**Draw 17**	0.635	0.635	0.714	0.477	0.504	0.496	0.578	0.429	0.596	0.752	0.649	0.500	0.542	0.632	0.482
	**Draw 18**	0.613	0.613	0.737	0.495	0.492	0.488	0.588	0.410	0.634	0.772	0.666	0.542	0.550	0.635	0.498
	**Draw 19**	0.643	0.643	0.700	0.500	0.497	0.503	0.571	0.421	0.647	0.727	0.648	0.500	0.554	0.662	0.507
	**Draw 20**	0.649	0.649	0.715	0.516	0.534	0.548	0.595	0.413	0.568	0.743	0.613	0.480	0.551	0.655	0.478
**DRAWS UP TO 150 VALVES**	**Draw 1**	0.708	0.719	0.742	0.484	0.495	0.496	0.565	0.408	0.637	0.701	0.649	0.500	0.555	0.648	0.482
	**Draw 2**	0.680	0.730	0.700	0.491	0.490	0.485	0.594	0.417	0.636	0.731	0.665	0.492	0.592	0.643	0.500
	**Draw 3**	0.731	0.742	0.719	0.511	0.475	0.494	0.576	0.412	0.608	0.727	0.678	0.513	0.546	0.642	0.523
	**Draw 4**	0.706	0.693	0.724	0.487	0.519	0.519	0.595	0.411	0.634	0.709	0.649	0.518	0.559	0.628	0.491
	**Draw 5**	0.672	0.767	0.739	0.494	0.492	0.512	0.570	0.412	0.598	0.711	0.692	0.487	0.568	0.637	0.498
	**Draw 6**	0.707	0.697	0.735	0.485	0.489	0.517	0.559	0.408	0.603	0.703	0.652	0.508	0.547	0.603	0.489
	**Draw 7**	0.688	0.670	0.757	0.506	0.483	0.489	0.605	0.413	0.641	0.704	0.677	0.502	0.577	0.656	0.497
	**Draw 8**	0.719	0.653	0.728	0.508	0.483	0.507	0.600	0.405	0.657	0.722	0.673	0.493	0.563	0.621	0.473
	**Draw 9**	0.701	0.742	0.725	0.487	0.484	0.496	0.597	0.406	0.606	0.737	0.681	0.527	0.575	0.651	0.506
	**Draw 10**	0.710	0.722	0.726	0.483	0.510	0.507	0.607	0.415	0.656	0.726	0.658	0.544	0.566	0.618	0.475
	**Draw 11**	0.682	0.625	0.705	0.492	0.491	0.503	0.580	0.413	0.618	0.725	0.626	0.524	0.575	0.656	0.485
	**Draw 12**	0.727	0.669	0.714	0.466	0.480	0.497	0.594	0.403	0.593	0.718	0.648	0.530	0.559	0.659	0.482
	**Draw 13**	0.692	0.714	0.738	0.506	0.476	0.542	0.573	0.405	0.608	0.724	0.681	0.493	0.574	0.628	0.477
	**Draw 14**	0.736	0.739	0.717	0.508	0.490	0.526	0.591	0.405	0.610	0.706	0.651	0.488	0.577	0.622	0.515
	**Draw 15**	0.717	0.711	0.725	0.494	0.484	0.520	0.597	0.397	0.656	0.755	0.677	0.508	0.554	0.630	0.500
	**Draw 16**	0.678	0.590	0.711	0.471	0.506	0.508	0.599	0.397	0.611	0.696	0.659	0.514	0.572	0.640	0.490
	**Draw 17**	0.716	0.709	0.691	0.498	0.500	0.479	0.582	0.408	0.622	0.700	0.647	0.490	0.554	0.633	0.503
	**Draw 18**	0.666	0.739	0.721	0.482	0.463	0.519	0.566	0.407	0.628	0.707	0.643	0.486	0.574	0.621	0.484
	**Draw 19**	0.694	0.723	0.734	0.503	0.500	0.511	0.577	0.415	0.653	0.736	0.688	0.500	0.553	0.667	0.475
	**Draw 20**	0.753	0.693	0.728	0.482	0.470	0.508	0.591	0.413	0.643	0.720	0.662	0.501	0.559	0.630	0.513
**DRAWS UP TO 00 VALVES**	**Draw 1**	0.705	0.691	0.708	0.495	0.494	0.503	0.576	0.416	0.624	0.732	0.661	0.522	0.565	0.612	0.511
	**Draw 2**	0.685	0.706	0.722	0.496	0.522	0.512	0.587	0.406	0.592	0.710	0.690	0.508	0.575	0.638	0.484
	**Draw 3**	0.718	0.707	0.715	0.498	0.500	0.493	0.595	0.408	0.607	0.701	0.660	0.504	0.547	0.650	0.502
	**Draw 4**	0.714	0.714	0.739	0.490	0.497	0.512	0.583	0.408	0.642	0.722	0.654	0.525	0.542	0.638	0.509
	**Draw 5**	0.713	0.721	0.714	0.496	0.482	0.509	0.581	0.411	0.614	0.697	0.636	0.496	0.544	0.639	0.490
	**Draw 6**	0.709	0.736	0.719	0.497	0.511	0.514	0.591	0.415	0.617	0.693	0.657	0.513	0.559	0.640	0.495
	**Draw 7**	0.709	0.724	0.723	0.497	0.492	0.493	0.590	0.411	0.604	0.722	0.635	0.525	0.564	0.631	0.482
	**Draw 8**	0.697	0.699	0.710	0.505	0.519	0.504	0.597	0.408	0.635	0.703	0.646	0.495	0.564	0.641	0.505
	**Draw 9**	0.679	0.726	0.731	0.488	0.502	0.514	0.596	0.411	0.644	0.733	0.647	0.523	0.562	0.640	0.477
	**Draw 10**	0.721	0.758	0.718	0.491	0.490	0.496	0.569	0.405	0.634	0.705	0.662	0.509	0.577	0.649	0.498
	**Draw 11**	0.729	0.710	0.727	0.499	0.493	0.514	0.589	0.407	0.605	0.724	0.656	0.509	0.570	0.629	0.491
	**Draw 12**	0.707	0.700	0.730	0.500	0.496	0.508	0.583	0.417	0.601	0.738	0.673	0.503	0.552	0.617	0.490
	**Draw 13**	0.690	0.717	0.731	0.501	0.506	0.504	0.606	0.403	0.633	0.707	0.647	0.530	0.550	0.628	0.514
	**Draw 14**	0.717	0.693	0.712	0.499	0.496	0.503	0.587	0.413	0.629	0.709	0.677	0.504	0.553	0.669	0.516
	**Draw 15**	0.714	0.725	0.720	0.493	0.512	0.494	0.568	0.407	0.596	0.711	0.667	0.483	0.571	0.668	0.505
	**Draw 16**	0.700	0.737	0.732	0.493	0.477	0.525	0.578	0.408	0.607	0.722	0.652	0.516	0.584	0.623	0.499
	**Draw 17**	0.736	0.703	0.739	0.502	0.506	0.505	0.611	0.411	0.596	0.727	0.669	0.536	0.550	0.627	0.487
	**Draw 18**	0.707	0.711	0.729	0.493	0.482	0.491	0.598	0.414	0.617	0.711	0.658	0.525	0.540	0.648	0.492
	**Draw 19**	0.702	0.736	0.748	0.485	0.515	0.486	0.590	0.411	0.609	0.699	0.652	0.486	0.574	0.622	0.511
	**Draw 20**	0.715	0.690	0.727	0.494	0.508	0.514	0.584	0.417	0.614	0.709	0.645	0.522	0.556	0.632	0.486
**DRAWS UP TO 250 VALVES**	**Draw 1**	0.699	0.699	0.721	0.490	0.510	0.517	0.575	0.408	0.612	0.711	0.674	0.493	0.558	0.659	0.498
	**Draw 2**	0.706	0.714	0.712	0.492	0.501	0.492	0.595	0.405	0.617	0.717	0.675	0.518	0.554	0.647	0.499
	**Draw 3**	0.726	0.712	0.712	0.490	0.481	0.497	0.596	0.412	0.619	0.714	0.682	0.520	0.580	0.654	0.495
	**Draw 4**	0.728	0.697	0.735	0.497	0.491	0.510	0.580	0.405	0.625	0.712	0.661	0.513	0.556	0.638	0.500
	**Draw 5**	0.694	0.714	0.713	0.495	0.479	0.508	0.592	0.410	0.614	0.710	0.654	0.509	0.574	0.631	0.497
	**Draw 6**	0.706	0.700	0.724	0.492	0.518	0.515	0.588	0.409	0.615	0.716	0.653	0.481	0.561	0.636	0.501
	**Draw 7**	0.697	0.701	0.728	0.497	0.490	0.513	0.597	0.408	0.610	0.705	0.636	0.527	0.548	0.641	0.502
	**Draw 8**	0.711	0.712	0.709	0.503	0.486	0.520	0.567	0.412	0.617	0.718	0.664	0.522	0.554	0.644	0.493
	**Draw 9**	0.688	0.685	0.709	0.490	0.499	0.507	0.572	0.410	0.610	0.736	0.649	0.522	0.582	0.631	0.497
	**Draw 10**	0.709	0.702	0.719	0.495	0.486	0.509	0.585	0.406	0.622	0.725	0.659	0.514	0.519	0.657	0.491
	**Draw 11**	0.730	0.700	0.718	0.493	0.485	0.510	0.605	0.410	0.596	0.741	0.653	0.526	0.554	0.661	0.508
	**Draw 12**	0.710	0.695	0.738	0.497	0.506	0.515	0.581	0.412	0.620	0.709	0.645	0.517	0.580	0.644	0.502
	**Draw 13**	0.695	0.716	0.717	0.496	0.485	0.511	0.588	0.412	0.636	0.710	0.652	0.500	0.557	0.641	0.491
	**Draw 14**	0.707	0.716	0.708	0.496	0.474	0.511	0.590	0.411	0.621	0.716	0.663	0.489	0.576	0.626	0.501
	**Draw 15**	0.712	0.702	0.716	0.497	0.488	0.515	0.585	0.408	0.610	0.732	0.662	0.488	0.549	0.624	0.503
	**Draw 16**	0.717	0.700	0.744	0.493	0.501	0.499	0.592	0.410	0.618	0.721	0.660	0.505	0.558	0.635	0.487
	**Draw 17**	0.724	0.719	0.736	0.497	0.492	0.529	0.587	0.414	0.628	0.715	0.643	0.514	0.571	0.635	0.487
	**Draw 18**	0.714	0.712	0.718	0.488	0.522	0.493	0.567	0.403	0.620	0.715	0.648	0.535	0.537	0.633	0.488
	**Draw 19**	0.706	0.732	0.718	0.492	0.514	0.518	0.583	0.408	0.613	0.711	0.642	0.522	0.564	0.635	0.480
	**Draw 20**	0.691	0.704	0.718	0.496	0.511	0.505	0.578	0.410	0.614	0.710	0.660	0.497	0.571	0.647	0.505
**DRAWS UP TO 300 VALVES**	**Draw 1**	0.713	0.708	0.711	0.486	0.490	0.513	0.584	0.409	0.617	0.723	0.647	0.521	0.576	0.649	0.505
	**Draw 2**	0.703	0.700	0.725	0.477	0.488	0.513	0.591	0.414	0.623	0.716	0.647	0.501	0.574	0.634	0.496
	**Draw 3**	0.700	0.734	0.728	0.494	0.520	0.508	0.581	0.403	0.606	0.716	0.648	0.520	0.546	0.637	0.498
	**Draw 4**	0.709	0.696	0.740	0.503	0.490	0.508	0.596	0.409	0.622	0.718	0.644	0.509	0.548	0.629	0.485
	**Draw 5**	0.711	0.698	0.717	0.482	0.499	0.503	0.601	0.408	0.626	0.724	0.669	0.501	0.560	0.643	0.498
	**Draw 6**	0.702	0.690	0.728	0.499	0.497	0.511	0.587	0.411	0.625	0.712	0.653	0.515	0.550	0.634	0.501
	**Draw 7**	0.707	0.690	0.717	0.504	0.512	0.512	0.597	0.413	0.619	0.721	0.666	0.508	0.551	0.635	0.500
	**Draw 8**	0.700	0.718	0.722	0.493	0.495	0.504	0.588	0.408	0.621	0.733	0.666	0.521	0.523	0.646	0.491
	**Draw 9**	0.698	0.718	0.721	0.496	0.501	0.507	0.592	0.412	0.616	0.724	0.648	0.500	0.558	0.650	0.502
	**Draw 10**	0.694	0.742	0.721	0.490	0.505	0.514	0.581	0.411	0.616	0.714	0.653	0.503	0.571	0.647	0.492
	**Draw 11**	0.707	0.729	0.717	0.497	0.489	0.507	0.581	0.407	0.619	0.710	0.659	0.504	0.579	0.643	0.489
	**Draw 12**	0.701	0.732	0.722	0.501	0.512	0.510	0.580	0.409	0.617	0.733	0.660	0.512	0.555	0.633	0.497
	**Draw 13**	0.693	0.714	0.718	0.496	0.502	0.509	0.583	0.411	0.630	0.715	0.646	0.524	0.557	0.646	0.497
	**Draw 14**	0.700	0.702	0.725	0.510	0.498	0.510	0.592	0.409	0.617	0.719	0.667	0.521	0.557	0.635	0.499
	**Draw 15**	0.724	0.660	0.731	0.504	0.490	0.508	0.579	0.410	0.617	0.711	0.662	0.503	0.552	0.636	0.494
	**Draw 16**	0.710	0.713	0.716	0.483	0.493	0.510	0.587	0.409	0.619	0.716	0.659	0.504	0.557	0.640	0.492
	**Draw 17**	0.716	0.714	0.724	0.493	0.490	0.508	0.591	0.411	0.618	0.720	0.663	0.518	0.561	0.638	0.490
	**Draw 18**	0.701	0.722	0.723	0.490	0.496	0.499	0.576	0.412	0.616	0.708	0.666	0.504	0.556	0.632	0.489
	**Draw 19**	0.713	0.730	0.712	0.488	0.499	0.509	0.569	0.413	0.623	0.730	0.657	0.513	0.553	0.646	0.495
	**Draw 20**	0.715	0.690	0.722	0.491	0.498	0.513	0.594	0.409	0.612	0.714	0.646	0.515	0.560	0.628	0.498
**DRAWS UP TO 350 VALVES**	**Draw 1**	0.714	0.736	0.713	0.500	0.504	0.506	0.587	0.410	0.615	0.720	0.643	0.511	0.567	0.636	0.500
	**Draw 2**	0.706	0.721	0.730	0.505	0.498	0.515	0.584	0.408	0.617	0.720	0.652	0.510	0.557	0.642	0.500
	**Draw 3**	0.710	0.718	0.720	0.500	0.505	0.509	0.579	0.410	0.626	0.724	0.650	0.513	0.565	0.642	0.498
	**Draw 4**	0.706	0.706	0.721	0.496	0.488	0.509	0.598	0.411	0.620	0.713	0.650	0.509	0.565	0.636	0.494
	**Draw 5**	0.707	0.689	0.722	0.507	0.495	0.509	0.584	0.409	0.614	0.718	0.654	0.521	0.568	0.639	0.499
	**Draw 6**	0.702	0.732	0.726	0.483	0.500	0.511	0.575	0.411	0.612	0.711	0.643	0.518	0.569	0.647	0.494
	**Draw 7**	0.709	0.690	0.729	0.494	0.477	0.506	0.595	0.409	0.618	0.714	0.656	0.513	0.548	0.642	0.491
	**Draw 8**	0.708	0.706	0.719	0.495	0.492	0.508	0.595	0.411	0.616	0.715	0.650	0.517	0.550	0.641	0.495
	**Draw 9**	0.712	0.730	0.716	0.487	0.505	0.513	0.588	0.412	0.619	0.719	0.644	0.525	0.552	0.647	0.492
	**Draw 10**	0.712	0.698	0.721	0.503	0.509	0.511	0.588	0.409	0.616	0.722	0.644	0.516	0.548	0.641	0.490
	**Draw 11**	0.707	0.693	0.720	0.491	0.489	0.508	0.574	0.408	0.611	0.721	0.633	0.504	0.558	0.633	0.503
	**Draw 12**	0.708	0.683	0.723	0.498	0.492	0.504	0.576	0.407	0.617	0.719	0.661	0.505	0.574	0.645	0.499
	**Draw 13**	0.699	0.722	0.719	0.514	0.505	0.509	0.587	0.408	0.618	0.713	0.648	0.508	0.548	0.640	0.496
	**Draw 14**	0.707	0.684	0.724	0.487	0.512	0.510	0.584	0.412	0.616	0.715	0.656	0.516	0.576	0.644	0.490
	**Draw 15**	0.707	0.711	0.719	0.493	0.499	0.510	0.585	0.410	0.620	0.723	0.648	0.511	0.583	0.644	0.495
	**Draw 16**	0.708	0.711	0.728	0.496	0.508	0.511	0.592	0.410	0.616	0.725	0.659	0.504	0.563	0.636	0.494
	**Draw 17**	0.713	0.700	0.721	0.489	0.491	0.509	0.577	0.409	0.623	0.722	0.650	0.517	0.548	0.643	0.496
	**Draw 18**	0.725	0.699	0.722	0.505	0.488	0.519	0.576	0.407	0.618	0.720	0.655	0.506	0.584	0.640	0.493
	**Draw 19**	0.704	0.718	0.719	0.510	0.502	0.513	0.589	0.412	0.620	0.722	0.640	0.511	0.560	0.642	0.491
	**Draw 20**	0.711	0.715	0.721	0.501	0.502	0.508	0.595	0.411	0.610	0.716	0.652	0.502	0.540	0.638	0.492
**DRAWS UP TO 400 VALVES**	**Draw 1**	0.709	0.695	0.724	0.496	0.501	0.515	0.585	0.409	0.622	0.708	0.657	0.514	0.551	0.637	0.494
	**Draw 2**	0.702	0.688	0.721	0.489	0.505	0.514	0.584	0.411	0.618	0.723	0.652	0.503	0.583	0.647	0.492
	**Draw 3**	0.708	0.700	0.719	0.488	0.493	0.514	0.588	0.409	0.618	0.716	0.652	0.515	0.559	0.646	0.489
	**Draw 4**	0.715	0.702	0.724	0.490	0.502	0.504	0.586	0.410	0.615	0.719	0.662	0.509	0.563	0.643	0.496
	**Draw 5**	0.710	0.688	0.726	0.495	0.508	0.514	0.581	0.411	0.616	0.715	0.652	0.513	0.566	0.639	0.497
	**Draw 6**	0.701	0.712	0.720	0.484	0.487	0.511	0.583	0.410	0.616	0.723	0.645	0.514	0.552	0.640	0.497
	**Draw 7**	0.702	0.679	0.724	0.496	0.493	0.517	0.588	0.410	0.616	0.717	0.646	0.517	0.551	0.638	0.493
	**Draw 8**	0.705	0.715	0.722	0.492	0.488	0.508	0.583	0.410	0.617	0.716	0.644	0.514	0.552	0.634	0.493
	**Draw 9**	0.710	0.722	0.724	0.495	0.505	0.521	0.581	0.410	0.622	0.725	0.654	0.511	0.541	0.635	0.491
	**Draw 10**	0.703	0.706	0.718	0.493	0.496	0.507	0.584	0.411	0.618	0.717	0.658	0.511	0.550	0.637	0.496
	**Draw 11**	0.710	0.716	0.725	0.494	0.494	0.507	0.585	0.410	0.619	0.718	0.658	0.512	0.565	0.639	0.494
	**Draw 12**	0.708	0.729	0.724	0.484	0.505	0.505	0.586	0.409	0.611	0.719	0.656	0.515	0.530	0.638	0.502
	**Draw 13**	0.713	0.715	0.717	0.496	0.490	0.510	0.584	0.409	0.616	0.715	0.668	0.510	0.579	0.641	0.490
	**Draw 14**	0.706	0.677	0.723	0.506	0.494	0.506	0.582	0.410	0.619	0.719	0.655	0.505	0.534	0.636	0.497
	**Draw 15**	0.709	0.721	0.724	0.501	0.493	0.517	0.588	0.409	0.615	0.720	0.649	0.509	0.582	0.639	0.489
	**Draw 16**	0.709	0.705	0.725	0.491	0.492	0.507	0.584	0.411	0.614	0.715	0.653	0.509	0.549	0.635	0.492
	**Draw 17**	0.715	0.691	0.721	0.492	0.493	0.505	0.588	0.410	0.618	0.720	0.653	0.512	0.556	0.640	0.494
	**Draw 18**	0.705	0.709	0.721	0.485	0.489	0.524	0.587	0.409	0.617	0.718	0.647	0.511	0.569	0.642	0.494
	**Draw 19**	0.702	0.735	0.725	0.489	0.499	0.508	0.581	0.409	0.613	0.722	0.643	0.510	0.566	0.634	0.498
	**Draw 20**	0.707	0.713	0.724	0.507	0.502	0.500	0.589	0.409	0.615	0.721	0.652	0.512	0.586	0.640	0.487
