# Supplementary material for: Is it possible to optimise the labour and time intensity of diatom analyses for determination of the Polish Diatom Indices (IO, IOJ)?
Source: Environ Monit Assess. 2022 Nov 3;195(1):64. doi: 10.1007/s10661-022-10676-7 (PMC9633445; doi:10.1007/s10661-022-10676-7)
Supplement: Supplementary file 4 — Supplementary file4 (PDF 254 KB) [file 10661_2022_10676_MOESM4_ESM.pdf]

Supplementary material 4

Supplementary Table G Results of drew values of Polish Diatom Index for rivers (IO) grouped by to primary studies results: GROUP 1 – very good ecological status; GROUP 2 –good ecological status; GROUP 3 – moderate ecological status

GROUP 1

	50_valve	100_valve	150_vavle	200_valve	250_valve	300_valve	350_valve	400_valve
**I1_draw1**	0.734	0.734	0.784	0.779	0.779	0.772	0.771	0.772
**I1_draw2**	0.754	0.780	0.768	0.785	0.778	0.775	0.772	0.773
**I1_draw3**	0.756	0.801	0.757	0.767	0.777	0.769	0.777	0.773
**I1_draw4**	0.759	0.796	0.774	0.786	0.763	0.771	0.766	0.772
**I1_draw5**	0.777	0.756	0.779	0.789	0.775	0.769	0.772	0.773
**I1_draw6**	0.767	0.766	0.764	0.781	0.771	0.772	0.768	0.770
**I1_draw7**	0.771	0.763	0.756	0.763	0.772	0.774	0.775	0.775
**I1_draw8**	0.780	0.793	0.748	0.761	0.783	0.765	0.767	0.773
**I1_draw9**	0.754	0.766	0.786	0.778	0.775	0.781	0.781	0.771
**I1_draw10**	0.716	0.758	0.779	0.764	0.773	0.780	0.774	0.772
**I1_draw11**	0.749	0.756	0.773	0.771	0.787	0.783	0.778	0.771
**I1_draw12**	0.805	0.777	0.770	0.767	0.754	0.777	0.767	0.773
**I1_draw13**	0.795	0.790	0.779	0.777	0.784	0.773	0.770	0.772
**I1_draw14**	0.777	0.776	0.765	0.765	0.780	0.778	0.777	0.774
**I1_draw15**	0.749	0.797	0.762	0.769	0.784	0.779	0.769	0.773
**I1_draw16**	0.756	0.781	0.787	0.774	0.777	0.788	0.768	0.771
**I1_draw17**	0.764	0.794	0.773	0.762	0.785	0.768	0.770	0.780
**I1_draw18**	0.787	0.776	0.768	0.780	0.765	0.775	0.772	0.771
**I1_draw19**	0.742	0.775	0.790	0.775	0.790	0.777	0.770	0.771
**I1_draw20**	0.807	0.789	0.759	0.776	0.778	0.768	0.771	0.773
**D1_draw1**	0.843	0.844	0.848	0.850	0.864	0.858	0.856	0.854
**D1_draw2**	0.867	0.864	0.845	0.847	0.857	0.859	0.856	0.854
**D1_draw3**	0.849	0.867	0.859	0.846	0.859	0.846	0.852	0.855
**D1_draw4**	0.886	0.829	0.856	0.852	0.855	0.849	0.853	0.855
**D1_draw5**	0.850	0.870	0.861	0.848	0.861	0.854	0.852	0.852
**D1_draw6**	0.861	0.851	0.846	0.863	0.841	0.849	0.853	0.852
**D1_draw7**	0.879	0.856	0.833	0.840	0.848	0.849	0.855	0.851
**D1_draw8**	0.838	0.867	0.867	0.844	0.862	0.862	0.851	0.855
**D1_draw9**	0.872	0.823	0.846	0.854	0.852	0.849	0.852	0.853
**D1_draw10**	0.876	0.849	0.823	0.849	0.842	0.861	0.857	0.852
**D1_draw11**	0.868	0.864	0.851	0.841	0.853	0.849	0.853	0.853
**D1_draw12**	0.839	0.861	0.855	0.852	0.853	0.857	0.854	0.853
**D1_draw13**	0.867	0.843	0.853	0.863	0.856	0.857	0.855	0.854
**D1_draw14**	0.862	0.868	0.853	0.857	0.865	0.849	0.854	0.852
**D1_draw15**	0.849	0.840	0.870	0.856	0.853	0.855	0.855	0.855
**D1_draw16**	0.880	0.853	0.868	0.839	0.856	0.852	0.855	0.855
**D1_draw17**	0.853	0.852	0.851	0.857	0.859	0.844	0.849	0.854
**D1_draw18**	0.800	0.840	0.811	0.849	0.855	0.851	0.851	0.853
**D1_draw19**	0.856	0.846	0.864	0.864	0.845	0.857	0.855	0.851
**D1_draw20**	0.823	0.862	0.861	0.843	0.853	0.853	0.855	0.856
**D3_draw1**	0.701	0.696	0.710	0.704	0.696	0.697	0.703	0.694
**D3_draw2**	0.777	0.697	0.718	0.685	0.698	0.686	0.696	0.697
**D3_draw3**	0.741	0.709	0.705	0.684	0.706	0.695	0.700	0.701
**D3_draw4**	0.736	0.714	0.700	0.717	0.709	0.705	0.700	0.703
**D3_draw5**	0.712	0.694	0.693	0.700	0.689	0.699	0.702	0.692
**D3_draw6**	0.740	0.713	0.678	0.700	0.686	0.692	0.691	0.691
**D3_draw7**	0.692	0.704	0.684	0.684	0.706	0.676	0.692	0.691
**D3_draw8**	0.702	0.732	0.705	0.693	0.696	0.681	0.694	0.692
**D3_draw9**	0.714	0.719	0.720	0.689	0.689	0.699	0.693	0.695
**D3_draw10**	0.719	0.702	0.700	0.705	0.695	0.688	0.699	0.700
**D3_draw11**	0.787	0.712	0.703	0.708	0.697	0.677	0.697	0.702
**D3_draw12**	0.670	0.688	0.690	0.697	0.690	0.702	0.697	0.689
**D3_draw13**	0.701	0.715	0.701	0.690	0.703	0.688	0.697	0.698
**D3_draw14**	0.694	0.708	0.707	0.696	0.703	0.687	0.702	0.698
**D3_draw15**	0.733	0.677	0.686	0.700	0.699	0.693	0.697	0.702
**D3_draw16**	0.686	0.701	0.694	0.690	0.694	0.690	0.704	0.693
**D3_draw17**	0.737	0.692	0.686	0.697	0.686	0.681	0.699	0.702
**D3_draw18**	0.670	0.703	0.686	0.683	0.707	0.681	0.701	0.694
**D3_draw19**	0.699	0.690	0.706	0.708	0.691	0.685	0.691	0.695
**D3_draw20**	0.660	0.733	0.699	0.702	0.688	0.697	0.687	0.694
**D4_draw1**	0.762	0.726	0.755	0.739	0.753	0.737	0.735	0.746
**D4_draw2**	0.760	0.742	0.771	0.750	0.745	0.737	0.746	0.747
**D4_draw3**	0.766	0.773	0.749	0.729	0.736	0.743	0.748	0.741
**D4_draw4**	0.766	0.753	0.742	0.742	0.734	0.741	0.744	0.746
**D4_draw5**	0.755	0.748	0.745	0.750	0.743	0.750	0.742	0.738
**D4_draw6**	0.727	0.763	0.751	0.769	0.738	0.745	0.748	0.740
**D4_draw7**	0.753	0.749	0.748	0.744	0.736	0.750	0.740	0.739
**D4_draw8**	0.747	0.737	0.746	0.734	0.723	0.754	0.742	0.742
**D4_draw9**	0.743	0.742	0.767	0.743	0.742	0.737	0.744	0.745
**D4_draw10**	0.738	0.707	0.741	0.748	0.742	0.736	0.741	0.746
**D4_draw11**	0.738	0.727	0.725	0.750	0.757	0.741	0.739	0.745
**D4_draw12**	0.763	0.768	0.735	0.751	0.755	0.743	0.750	0.745
**D4_draw13**	0.787	0.754	0.766	0.752	0.747	0.744	0.753	0.744
**D4_draw14**	0.721	0.734	0.745	0.735	0.755	0.743	0.737	0.748
**D4_draw15**	0.706	0.732	0.768	0.743	0.750	0.745	0.746	0.749
**D4_draw16**	0.753	0.756	0.734	0.748	0.746	0.731	0.743	0.745
**D4_draw17**	0.710	0.747	0.734	0.719	0.752	0.749	0.743	0.741
**D4_draw18**	0.747	0.731	0.742	0.739	0.750	0.736	0.742	0.739
**D4_draw19**	0.729	0.739	0.730	0.745	0.756	0.746	0.748	0.740
**D4_draw20**	0.732	0.783	0.748	0.750	0.741	0.745	0.742	0.743
**MIN**	0.660	0.677	0.678	0.683	0.686	0.676	0.687	0.689
**MAX**	0.886	0.870	0.870	0.864	0.865	0.862	0.857	0.856
**DISPERSION**	0.226	0.193	0.191	0.181	0.179	0.185	0.170	0.166
**MEDIANA**	0.756	0.760	0.761	0.762	0.757	0.759	0.759	0.760

GROUP 2

	50_valve	100_valve	150_vavle	200_valve	250_valve	300_valve	350_valve	400_valve
**I2_draw1**	0.687	0.668	0.671	0.657	0.691	0.660	0.663	0.669
**I2_draw2**	0.678	0.645	0.690	0.673	0.679	0.663	0.665	0.668
**I2_draw3**	0.689	0.659	0.643	0.660	0.660	0.673	0.664	0.672
**I2_draw4**	0.681	0.638	0.675	0.667	0.675	0.672	0.666	0.670
**I2_draw5**	0.670	0.670	0.679	0.648	0.679	0.666	0.661	0.667
**I2_draw6**	0.661	0.649	0.669	0.672	0.671	0.671	0.667	0.666
**I2_draw7**	0.659	0.689	0.686	0.679	0.670	0.670	0.670	0.670
**I2_draw8**	0.686	0.675	0.671	0.666	0.665	0.666	0.659	0.668
**I2_draw9**	0.693	0.671	0.689	0.670	0.655	0.670	0.668	0.666
**I2_draw10**	0.683	0.621	0.676	0.665	0.688	0.673	0.675	0.669
**I2_draw11**	0.713	0.673	0.637	0.658	0.682	0.671	0.672	0.668
**I2_draw12**	0.629	0.679	0.639	0.686	0.651	0.673	0.666	0.667
**I2_draw13**	0.686	0.697	0.684	0.659	0.685	0.683	0.667	0.666
**I2_draw14**	0.620	0.640	0.679	0.661	0.664	0.663	0.669	0.671
**I2_draw15**	0.636	0.681	0.652	0.658	0.655	0.677	0.674	0.665
**I2_draw16**	0.639	0.671	0.662	0.655	0.665	0.661	0.674	0.674
**I2_draw17**	0.649	0.639	0.662	0.660	0.678	0.672	0.670	0.668
**I2_draw18**	0.669	0.689	0.685	0.649	0.685	0.662	0.663	0.665
**I2_draw19**	0.744	0.698	0.676	0.659	0.674	0.678	0.666	0.673
**I2_draw20**	0.698	0.674	0.668	0.668	0.668	0.656	0.668	0.664
**I3_draw1**	0.610	0.614	0.604	0.628	0.618	0.615	0.611	0.596
**I3_draw2**	0.601	0.604	0.607	0.616	0.627	0.615	0.621	0.610
**I3_draw3**	0.674	0.636	0.619	0.612	0.611	0.616	0.609	0.617
**I3_draw4**	0.593	0.628	0.615	0.605	0.619	0.607	0.609	0.612
**I3_draw5**	0.574	0.623	0.643	0.617	0.618	0.627	0.613	0.613
**I3_draw6**	0.600	0.614	0.595	0.622	0.604	0.609	0.615	0.608
**I3_draw7**	0.592	0.623	0.623	0.609	0.617	0.618	0.619	0.630
**I3_draw8**	0.654	0.619	0.607	0.629	0.621	0.616	0.618	0.612
**I3_draw9**	0.619	0.629	0.614	0.623	0.603	0.617	0.620	0.613
**I3_draw10**	0.615	0.620	0.633	0.613	0.614	0.617	0.631	0.626
**I3_draw11**	0.655	0.617	0.626	0.601	0.614	0.612	0.630	0.627
**I3_draw12**	0.658	0.594	0.585	0.614	0.619	0.611	0.615	0.631
**I3_draw13**	0.599	0.639	0.616	0.617	0.611	0.622	0.625	0.633
**I3_draw14**	0.597	0.595	0.608	0.600	0.618	0.628	0.591	0.623
**I3_draw15**	0.610	0.621	0.630	0.603	0.625	0.615	0.628	0.608
**I3_draw16**	0.601	0.607	0.617	0.608	0.613	0.621	0.619	0.614
**I3_draw17**	0.607	0.591	0.616	0.629	0.612	0.618	0.601	0.617
**I3_draw18**	0.596	0.599	0.618	0.621	0.603	0.611	0.645	0.622
**I3_draw19**	0.663	0.609	0.623	0.592	0.613	0.625	0.608	0.620
**I3_draw20**	0.605	0.623	0.606	0.620	0.619	0.614	0.636	0.616
**I4_draw1**	0.676	0.620	0.612	0.627	0.637	0.672	0.671	0.652
**I4_draw2**	0.650	0.610	0.611	0.605	0.667	0.663	0.681	0.664
**I4_draw3**	0.621	0.624	0.632	0.647	0.657	0.659	0.668	0.666
**I4_draw4**	0.681	0.627	0.621	0.615	0.674	0.669	0.664	0.663
**I4_draw5**	0.635	0.614	0.614	0.628	0.690	0.659	0.668	0.659
**I4_draw6**	0.646	0.618	0.602	0.617	0.665	0.660	0.669	0.655
**I4_draw7**	0.602	0.602	0.622	0.624	0.692	0.667	0.675	0.672
**I4_draw8**	0.658	0.605	0.624	0.610	0.668	0.668	0.681	0.667
**I4_draw9**	0.671	0.639	0.621	0.610	0.688	0.668	0.669	0.658
**I4_draw10**	0.621	0.628	0.607	0.605	0.658	0.669	0.668	0.676
**I4_draw11**	0.623	0.622	0.618	0.620	0.640	0.662	0.671	0.657
**I4_draw12**	0.615	0.629	0.619	0.647	0.645	0.655	0.654	0.668
**I4_draw13**	0.596	0.619	0.644	0.630	0.669	0.661	0.655	0.672
**I4_draw14**	0.607	0.657	0.618	0.611	0.650	0.664	0.669	0.688
**I4_draw15**	0.591	0.623	0.631	0.624	0.676	0.688	0.686	0.668
**I4_draw16**	0.629	0.657	0.625	0.625	0.691	0.663	0.698	0.678
**I4_draw17**	0.539	0.637	0.626	0.631	0.671	0.669	0.673	0.653
**I4_draw18**	0.603	0.618	0.618	0.620	0.675	0.658	0.672	0.660
**I4_draw19**	0.618	0.635	0.636	0.606	0.687	0.676	0.682	0.668
**I4_draw20**	0.615	0.562	0.626	0.627	0.660	0.687	0.692	0.683
**I5a_draw1**	0.605	0.569	0.582	0.585	0.573	0.586	0.581	0.605
**I5a_draw2**	0.578	0.569	0.584	0.585	0.569	0.573	0.571	0.579
**I5a_draw3**	0.580	0.607	0.592	0.584	0.572	0.558	0.582	0.585
**I5a_draw4**	0.619	0.573	0.582	0.585	0.577	0.593	0.589	0.597
**I5a_draw5**	0.555	0.560	0.585	0.582	0.605	0.595	0.611	0.585
**I5a_draw6**	0.636	0.593	0.582	0.586	0.583	0.583	0.585	0.592
**I5a_draw7**	0.564	0.589	0.588	0.590	0.586	0.591	0.576	0.586
**I5a_draw8**	0.588	0.589	0.580	0.585	0.622	0.567	0.567	0.588
**I5a_draw9**	0.584	0.560	0.602	0.584	0.583	0.589	0.587	0.590
**I5a_draw10**	0.630	0.566	0.581	0.585	0.590	0.594	0.605	0.589
**I5a_draw11**	0.566	0.583	0.567	0.583	0.591	0.578	0.608	0.600
**I5a_draw12**	0.605	0.585	0.583	0.586	0.598	0.581	0.582	0.602
**I5a_draw13**	0.631	0.573	0.588	0.586	0.612	0.586	0.569	0.588
**I5a_draw14**	0.599	0.589	0.590	0.588	0.587	0.582	0.603	0.579
**I5a_draw15**	0.588	0.593	0.575	0.583	0.590	0.582	0.589	0.590
**I5a_draw16**	0.589	0.551	0.569	0.587	0.595	0.589	0.588	0.601
**I5a_draw17**	0.576	0.595	0.593	0.587	0.572	0.586	0.584	0.598
**I5a_draw18**	0.569	0.599	0.579	0.588	0.603	0.573	0.570	0.605
**I5a_draw19**	0.522	0.575	0.599	0.584	0.564	0.593	0.557	0.582
**I5a_draw20**	0.580	0.604	0.595	0.582	0.600	0.595	0.573	0.604
**I5b_draw1**	0.646	0.665	0.648	0.644	0.641	0.646	0.632	0.649
**I5b_draw2**	0.675	0.642	0.653	0.645	0.659	0.662	0.651	0.647
**I5b_draw3**	0.637	0.635	0.661	0.658	0.656	0.621	0.637	0.653
**I5b_draw4**	0.667	0.661	0.660	0.647	0.629	0.660	0.649	0.644
**I5b_draw5**	0.650	0.648	0.656	0.648	0.647	0.646	0.645	0.645
**I5b_draw6**	0.654	0.660	0.641	0.652	0.671	0.671	0.650	0.646
**I5b_draw7**	0.616	0.650	0.636	0.639	0.648	0.668	0.669	0.652
**I5b_draw8**	0.626	0.674	0.657	0.642	0.662	0.653	0.649	0.637
**I5b_draw9**	0.633	0.656	0.640	0.640	0.626	0.661	0.633	0.642
**I5b_draw10**	0.664	0.647	0.648	0.650	0.654	0.643	0.648	0.633
**I5b_draw11**	0.679	0.651	0.635	0.649	0.641	0.658	0.637	0.657
**I5b_draw12**	0.636	0.639	0.658	0.661	0.650	0.636	0.651	0.651
**I5b_draw13**	0.621	0.662	0.658	0.656	0.656	0.654	0.653	0.638
**I5b_draw14**	0.645	0.652	0.653	0.643	0.648	0.643	0.639	0.657
**I5b_draw15**	0.633	0.640	0.633	0.641	0.671	0.634	0.659	0.637
**I5b_draw16**	0.658	0.663	0.650	0.647	0.652	0.666	0.645	0.656
**I5b_draw17**	0.653	0.625	0.669	0.644	0.665	0.646	0.660	0.643
**I5b_draw18**	0.598	0.647	0.650	0.646	0.659	0.647	0.631	0.643
**I5b_draw19**	0.622	0.650	0.652	0.652	0.653	0.657	0.648	0.639
**I5b_draw20**	0.647	0.639	0.633	0.638	0.634	0.656	0.649	0.666
**I6_draw1**	0.504	0.521	0.534	0.531	0.524	0.522	0.525	0.521
**I6_draw2**	0.568	0.535	0.525	0.515	0.522	0.527	0.524	0.523
**I6_draw3**	0.522	0.520	0.516	0.517	0.529	0.532	0.523	0.526
**I6_draw4**	0.534	0.504	0.517	0.527	0.540	0.516	0.522	0.527
**I6_draw5**	0.550	0.498	0.544	0.514	0.528	0.523	0.523	0.516
**I6_draw6**	0.558	0.514	0.525	0.516	0.528	0.532	0.526	0.524
**I6_draw7**	0.492	0.533	0.528	0.523	0.525	0.529	0.525	0.525
**I6_draw8**	0.532	0.536	0.537	0.519	0.533	0.527	0.524	0.529
**I6_draw9**	0.492	0.553	0.547	0.547	0.529	0.524	0.523	0.523
**I6_draw10**	0.498	0.486	0.519	0.530	0.530	0.528	0.524	0.520
**I6_draw11**	0.576	0.520	0.536	0.531	0.532	0.521	0.527	0.520
**I6_draw12**	0.459	0.527	0.504	0.548	0.530	0.514	0.521	0.523
**I6_draw13**	0.531	0.533	0.520	0.514	0.526	0.531	0.524	0.528
**I6_draw14**	0.492	0.545	0.517	0.542	0.523	0.519	0.527	0.521
**I6_draw15**	0.496	0.561	0.532	0.526	0.512	0.526	0.522	0.527
**I6_draw16**	0.527	0.559	0.544	0.534	0.521	0.519	0.527	0.532
**I6_draw17**	0.517	0.519	0.533	0.525	0.525	0.524	0.523	0.522
**I6_draw18**	0.517	0.483	0.538	0.526	0.512	0.515	0.525	0.528
**I6_draw19**	0.499	0.501	0.529	0.517	0.522	0.527	0.524	0.523
**I6_draw20**	0.560	0.526	0.539	0.535	0.532	0.527	0.527	0.527
**I7_draw1**	0.529	0.577	0.560	0.552	0.550	0.553	0.547	0.545
**I7_draw2**	0.521	0.573	0.559	0.556	0.562	0.565	0.563	0.558
**I7_draw3**	0.546	0.561	0.560	0.561	0.549	0.562	0.539	0.561
**I7_draw4**	0.551	0.554	0.558	0.563	0.548	0.570	0.544	0.538
**I7_draw5**	0.553	0.573	0.574	0.556	0.555	0.557	0.569	0.571
**I7_draw6**	0.593	0.563	0.564	0.548	0.550	0.554	0.542	0.558
**I7_draw7**	0.548	0.534	0.558	0.551	0.559	0.554	0.569	0.539
**I7_draw8**	0.578	0.527	0.569	0.559	0.552	0.560	0.548	0.545
**I7_draw9**	0.529	0.530	0.554	0.566	0.548	0.563	0.565	0.560
**I7_draw10**	0.549	0.558	0.562	0.561	0.556	0.560	0.554	0.569
**I7_draw11**	0.553	0.576	0.546	0.553	0.559	0.552	0.534	0.546
**I7_draw12**	0.568	0.528	0.571	0.566	0.554	0.549	0.558	0.550
**I7_draw13**	0.533	0.531	0.574	0.560	0.562	0.565	0.557	0.527
**I7_draw14**	0.554	0.570	0.556	0.561	0.569	0.563	0.579	0.577
**I7_draw15**	0.558	0.570	0.558	0.567	0.556	0.532	0.563	0.543

GROUP 3

	50_valve	100_valve	150_vavle	200_valve	250_valve	300_valve	350_valve	400_valve
**I9b_draw1**	0.488	0.498	0.477	0.482	0.478	0.486	0.494	0.491
**I9b_draw2**	0.468	0.493	0.477	0.486	0.492	0.483	0.493	0.485
**I9b_draw3**	0.505	0.512	0.479	0.487	0.489	0.475	0.488	0.465
**I9b_draw4**	0.507	0.512	0.492	0.484	0.484	0.489	0.478	0.482
**I9b_draw5**	0.480	0.521	0.483	0.478	0.479	0.485	0.486	0.497
**I9b_draw6**	0.447	0.472	0.476	0.480	0.480	0.493	0.482	0.477
**I9b_draw7**	0.485	0.492	0.476	0.489	0.469	0.475	0.483	0.477
**I9b_draw8**	0.487	0.488	0.483	0.487	0.473	0.490	0.485	0.492
**I9b_draw9**	0.509	0.485	0.480	0.485	0.494	0.484	0.489	0.492
**I9b_draw10**	0.516	0.499	0.493	0.485	0.481	0.474	0.475	0.484
**I9b_draw11**	0.508	0.488	0.486	0.482	0.496	0.479	0.487	0.493
**I9b_draw12**	0.492	0.482	0.476	0.483	0.500	0.474	0.483	0.483
**I9b_draw13**	0.535	0.495	0.494	0.482	0.480	0.487	0.496	0.463
**I9b_draw14**	0.460	0.475	0.496	0.486	0.484	0.485	0.484	0.460
**I9b_draw15**	0.489	0.470	0.490	0.484	0.487	0.482	0.501	0.490
**I9b_draw16**	0.508	0.469	0.493	0.486	0.482	0.483	0.471	0.476
**I9b_draw17**	0.503	0.462	0.509	0.483	0.481	0.487	0.486	0.488
**I9b_draw18**	0.470	0.480	0.485	0.484	0.475	0.480	0.497	0.491
**I9b_draw19**	0.456	0.489	0.487	0.481	0.491	0.479	0.481	0.465
**I9b_draw20**	0.452	0.488	0.479	0.485	0.493	0.477	0.489	0.489
**I11_draw1**	0.497	0.483	0.517	0.465	0.481	0.481	0.493	0.487
**I11_draw2**	0.487	0.490	0.486	0.489	0.491	0.482	0.491	0.479
**I11_draw3**	0.550	0.485	0.495	0.482	0.490	0.494	0.491	0.489
**I11_draw4**	0.472	0.543	0.493	0.498	0.488	0.495	0.491	0.485
**I11_draw5**	0.533	0.502	0.494	0.481	0.497	0.494	0.490	0.496
**I11_draw6**	0.535	0.499	0.501	0.479	0.490	0.490	0.491	0.496
**I11_draw7**	0.503	0.507	0.502	0.495	0.497	0.488	0.490	0.488
**I11_draw8**	0.531	0.489	0.521	0.482	0.482	0.494	0.493	0.490
**I11_draw9**	0.448	0.532	0.490	0.491	0.484	0.483	0.492	0.488
**I11_draw10**	0.531	0.458	0.497	0.501	0.481	0.498	0.491	0.492
**I11_draw11**	0.417	0.489	0.484	0.487	0.495	0.484	0.490	0.490
**I11_draw12**	0.498	0.499	0.482	0.487	0.491	0.491	0.490	0.497
**I11_draw13**	0.552	0.475	0.479	0.502	0.490	0.498	0.490	0.488
**I11_draw14**	0.499	0.499	0.497	0.477	0.487	0.487	0.491	0.488
**I11_draw15**	0.510	0.495	0.508	0.487	0.503	0.484	0.491	0.491
**I11_draw16**	0.511	0.478	0.486	0.484	0.495	0.486	0.491	0.488
**I11_draw17**	0.466	0.510	0.489	0.493	0.491	0.496	0.490	0.494
**I11_draw18**	0.484	0.497	0.472	0.493	0.491	0.487	0.490	0.497
**I11_draw19**	0.510	0.473	0.468	0.485	0.484	0.488	0.490	0.489
**I11_draw20**	0.445	0.496	0.470	0.492	0.483	0.494	0.491	0.495
**I12_draw1**	0.532	0.486	0.471	0.463	0.474	0.461	0.472	0.466
**I12_draw2**	0.484	0.483	0.461	0.469	0.472	0.479	0.475	0.463
**I12_draw3**	0.530	0.430	0.457	0.467	0.470	0.473	0.476	0.476
**I12_draw4**	0.411	0.494	0.454	0.458	0.465	0.471	0.469	0.468
**I12_draw5**	0.479	0.478	0.459	0.462	0.469	0.469	0.465	0.466
**I12_draw6**	0.467	0.479	0.470	0.468	0.473	0.468	0.470	0.469
**I12_draw7**	0.464	0.485	0.489	0.475	0.469	0.472	0.472	0.471
**I12_draw8**	0.438	0.464	0.456	0.476	0.472	0.472	0.461	0.484
**I12_draw9**	0.480	0.460	0.468	0.465	0.470	0.461	0.473	0.480
**I12_draw10**	0.468	0.467	0.469	0.473	0.469	0.470	0.456	0.462
**I12_draw11**	0.450	0.456	0.482	0.479	0.475	0.469	0.474	0.470
**I12_draw12**	0.507	0.444	0.481	0.460	0.475	0.476	0.477	0.466
**I12_draw13**	0.433	0.480	0.454	0.463	0.464	0.477	0.465	0.480
**I12_draw14**	0.491	0.471	0.475	0.478	0.469	0.476	0.460	0.483
**I12_draw15**	0.475	0.479	0.489	0.470	0.469	0.477	0.473	0.462
**I12_draw16**	0.447	0.443	0.465	0.476	0.475	0.469	0.481	0.469
**I12_draw17**	0.478	0.472	0.456	0.488	0.472	0.477	0.472	0.473
**I12_draw18**	0.491	0.485	0.483	0.482	0.469	0.469	0.489	0.476
**I12_draw19**	0.461	0.452	0.481	0.465	0.465	0.477	0.471	0.456
**I12_draw20**	0.458	0.446	0.468	0.465	0.473	0.474	0.459	0.468
**I13_draw1**	0.476	0.477	0.461	0.446	0.458	0.466	0.453	0.457
**I13_draw2**	0.439	0.456	0.443	0.450	0.464	0.458	0.456	0.457
**I13_draw3**	0.466	0.436	0.464	0.474	0.466	0.457	0.463	0.463
**I13_draw4**	0.373	0.455	0.465	0.447	0.454	0.459	0.457	0.457
**I13_draw5**	0.497	0.463	0.473	0.448	0.456	0.458	0.459	0.457
**I13_draw6**	0.469	0.461	0.451	0.455	0.462	0.452	0.461	0.463
**I13_draw7**	0.452	0.444	0.456	0.452	0.476	0.462	0.467	0.456
**I13_draw8**	0.419	0.468	0.465	0.456	0.459	0.475	0.456	0.461
**I13_draw9**	0.464	0.457	0.469	0.470	0.454	0.461	0.456	0.459
**I13_draw10**	0.473	0.468	0.439	0.461	0.464	0.456	0.457	0.458
**I13_draw11**	0.487	0.473	0.453	0.461	0.442	0.467	0.454	0.451
**I13_draw12**	0.455	0.457	0.450	0.449	0.468	0.461	0.452	0.467
**I13_draw13**	0.433	0.464	0.445	0.455	0.457	0.459	0.464	0.460
**I13_draw14**	0.464	0.473	0.458	0.496	0.468	0.460	0.456	0.460
**I13_draw15**	0.450	0.471	0.456	0.457	0.466	0.468	0.465	0.463
**I13_draw16**	0.423	0.469	0.473	0.485	0.471	0.456	0.450	0.455
**I13_draw17**	0.446	0.468	0.452	0.479	0.469	0.452	0.460	0.457
**I13_draw18**	0.463	0.450	0.456	0.456	0.450	0.454	0.465	0.462
**I13_draw19**	0.417	0.449	0.457	0.473	0.464	0.460	0.469	0.457
**I13_draw20**	0.402	0.418	0.443	0.455	0.461	0.456	0.450	0.457
**I22_draw1**	0.472	0.456	0.499	0.478	0.473	0.468	0.469	0.473
**I22_draw2**	0.481	0.500	0.490	0.477	0.478	0.468	0.468	0.474
**I22_draw3**	0.464	0.465	0.458	0.478	0.499	0.470	0.471	0.474
**I22_draw4**	0.458	0.492	0.475	0.467	0.485	0.463	0.474	0.476
**I22_draw5**	0.494	0.455	0.470	0.464	0.470	0.463	0.471	0.471
**I22_draw6**	0.445	0.474	0.478	0.471	0.476	0.472	0.471	0.472
**I22_draw7**	0.464	0.521	0.472	0.460	0.465	0.477	0.473	0.471
**I22_draw8**	0.473	0.481	0.477	0.474	0.469	0.465	0.473	0.471
**I22_draw9**	0.469	0.501	0.472	0.481	0.471	0.475	0.473	0.474
**I22_draw10**	0.376	0.483	0.455	0.464	0.476	0.473	0.474	0.473
**I22_draw11**	0.419	0.497	0.472	0.463	0.478	0.478	0.470	0.471
**I22_draw12**	0.489	0.476	0.479	0.467	0.468	0.466	0.472	0.474
**I22_draw13**	0.497	0.446	0.466	0.495	0.485	0.471	0.473	0.470
**I22_draw14**	0.464	0.458	0.472	0.461	0.482	0.468	0.473	0.475
**I22_draw15**	0.503	0.475	0.464	0.482	0.485	0.479	0.470	0.471
**I22_draw16**	0.441	0.449	0.500	0.468	0.477	0.462	0.473	0.469
**I22_draw17**	0.477	0.478	0.466	0.486	0.462	0.479	0.475	0.472
**I22_draw18**	0.439	0.483	0.471	0.462	0.476	0.479	0.472	0.472
**I22_draw19**	0.482	0.476	0.483	0.476	0.479	0.476	0.467	0.468
**I22_draw20**	0.467	0.488	0.475	0.472	0.476	0.472	0.473	0.477
**I24_draw1**	0.331	0.409	0.398	0.388	0.389	0.396	0.402	0.394
**I24_draw2**	0.372	0.391	0.398	0.375	0.381	0.396	0.389	0.386
**I24_draw3**	0.382	0.393	0.419	0.391	0.387	0.372	0.395	0.389
**I24_draw4**	0.440	0.413	0.373	0.358	0.393	0.400	0.392	0.394
**I24_draw5**	0.355	0.397	0.396	0.391	0.389	0.390	0.393	0.387
**I24_draw6**	0.399	0.368	0.379	0.405	0.399	0.386	0.387	0.398
**I24_draw7**	0.408	0.400	0.376	0.364	0.400	0.393	0.393	0.386
**I24_draw8**	0.395	0.395	0.404	0.416	0.391	0.400	0.387	0.389
**I24_draw9**	0.359	0.383	0.381	0.392	0.398	0.405	0.395	0.398
**I24_draw10**	0.416	0.377	0.389	0.413	0.399	0.388	0.396	0.385
**I24_draw11**	0.415	0.389	0.389	0.380	0.392	0.376	0.394	0.388
**I24_draw12**	0.388	0.405	0.384	0.381	0.388	0.387	0.401	0.395
**I24_draw13**	0.301	0.447	0.379	0.375	0.358	0.403	0.393	0.395
**I24_draw14**	0.398	0.429	0.394	0.380	0.387	0.396	0.389	0.387
**I24_draw15**	0.374	0.414	0.371	0.396	0.378	0.387	0.401	0.389
**I24_draw16**	0.335	0.395	0.370	0.382	0.394	0.383	0.392	0.394
**I24_draw17**	0.306	0.379	0.374	0.366	0.381	0.382	0.392	0.393
**I24_draw18**	0.420	0.376	0.431	0.365	0.401	0.392	0.392	0.392
**I24_draw19**	0.385	0.411	0.389	0.382	0.374	0.414	0.397	0.388
**I24_draw20**	0.405	0.412	0.377	0.388	0.407	0.378	0.387	0.388
**D6_draw1**	0.540	0.502	0.497	0.505	0.500	0.496	0.500	0.505
**D6_draw2**	0.480	0.493	0.490	0.494	0.515	0.504	0.497	0.499
**D6_draw3**	0.465	0.511	0.489	0.520	0.511	0.481	0.507	0.502
**D6_draw4**	0.536	0.489	0.492	0.505	0.487	0.490	0.510	0.509
**D6_draw5**	0.459	0.524	0.497	0.472	0.521	0.491	0.503	0.504
**D6_draw6**	0.499	0.517	0.471	0.501	0.515	0.497	0.498	0.503
**D6_draw7**	0.515	0.459	0.508	0.503	0.485	0.519	0.503	0.508
**D6_draw8**	0.461	0.515	0.529	0.477	0.500	0.512	0.496	0.503
**D6_draw9**	0.600	0.491	0.524	0.508	0.486	0.514	0.501	0.506
**D6_draw10**	0.504	0.557	0.496	0.492	0.491	0.507	0.510	0.502
**D6_draw11**	0.393	0.516	0.500	0.499	0.495	0.506	0.497	0.505
**D6_draw12**	0.522	0.505	0.487	0.504	0.530	0.503	0.506	0.498
**D6_draw13**	0.515	0.514	0.494	0.494	0.495	0.514	0.508	0.502
**D6_draw14**	0.507	0.513	0.498	0.497	0.517	0.503	0.491	0.506
**D6_draw15**	0.491	0.487	0.491	0.495	0.492	0.492	0.500	0.509

**I7_draw16**	0.614	0.560	0.550	0.560	0.553	0.563	0.560	0.561
**I7_draw17**	0.514	0.607	0.563	0.556	0.552	0.555	0.548	0.573
**I7_draw18**	0.570	0.578	0.565	0.557	0.561	0.574	0.563	0.558
**I7_draw19**	0.587	0.578	0.551	0.554	0.573	0.552	0.551	0.582
**I7_draw20**	0.554	0.576	0.575	0.551	0.550	0.542	0.577	0.560
**I8_draw1**	0.558	0.514	0.548	0.524	0.511	0.517	0.533	0.519
**I8_draw2**	0.560	0.517	0.529	0.526	0.523	0.528	0.520	0.518
**I8_draw3**	0.493	0.529	0.514	0.521	0.530	0.515	0.517	0.533
**I8_draw4**	0.561	0.525	0.526	0.526	0.520	0.522	0.514	0.539
**I8_draw5**	0.509	0.539	0.534	0.525	0.516	0.515	0.513	0.523
**I8_draw6**	0.570	0.509	0.524	0.534	0.520	0.530	0.530	0.513
**I8_draw7**	0.538	0.520	0.521	0.525	0.531	0.520	0.521	0.533
**I8_draw8**	0.535	0.506	0.524	0.522	0.536	0.528	0.513	0.515
**I8_draw9**	0.501	0.517	0.534	0.523	0.518	0.533	0.531	0.539
**I8_draw10**	0.520	0.544	0.525	0.527	0.522	0.534	0.513	0.536
**I8_draw11**	0.566	0.496	0.524	0.522	0.542	0.511	0.538	0.522
**I8_draw12**	0.496	0.530	0.531	0.519	0.522	0.530	0.540	0.524
**I8_draw13**	0.503	0.549	0.521	0.533	0.527	0.525	0.511	0.543
**I8_draw14**	0.518	0.527	0.526	0.519	0.538	0.519	0.542	0.516
**I8_draw15**	0.521	0.519	0.521	0.529	0.514	0.535	0.512	0.525
**I8_draw16**	0.489	0.528	0.506	0.512	0.532	0.528	0.529	0.527
**I8_draw17**	0.511	0.535	0.534	0.522	0.521	0.514	0.531	0.520
**I8_draw18**	0.518	0.493	0.535	0.531	0.524	0.520	0.528	0.525
**I8_draw19**	0.512	0.539	0.512	0.526	0.524	0.519	0.517	0.533
**I8_draw20**	0.528	0.545	0.531	0.514	0.519	0.526	0.525	0.519
**I9a_draw1**	0.510	0.535	0.520	0.504	0.517	0.518	0.509	0.505
**I9a_draw2**	0.538	0.527	0.522	0.525	0.511	0.501	0.520	0.507
**I9a_draw3**	0.475	0.498	0.509	0.514	0.520	0.516	0.518	0.518
**I9a_draw4**	0.536	0.507	0.521	0.501	0.515	0.513	0.514	0.514
**I9a_draw5**	0.523	0.518	0.502	0.503	0.505	0.520	0.505	0.505
**I9a_draw6**	0.538	0.474	0.520	0.513	0.512	0.512	0.511	0.515
**I9a_draw7**	0.521	0.512	0.511	0.508	0.516	0.514	0.524	0.519
**I9a_draw8**	0.478	0.490	0.530	0.519	0.512	0.524	0.508	0.519
**I9a_draw9**	0.521	0.539	0.514	0.512	0.509	0.520	0.518	0.516
**I9a_draw10**	0.508	0.527	0.517	0.518	0.512	0.516	0.513	0.513
**I9a_draw11**	0.490	0.487	0.513	0.515	0.514	0.512	0.522	0.526
**I9a_draw12**	0.559	0.526	0.535	0.504	0.513	0.515	0.495	0.508
**I9a_draw13**	0.475	0.526	0.510	0.507	0.512	0.503	0.503	0.515
**I9a_draw14**	0.509	0.490	0.509	0.509	0.510	0.530	0.498	0.490
**I9a_draw15**	0.533	0.517	0.509	0.515	0.516	0.519	0.507	0.513
**I9a_draw16**	0.479	0.497	0.522	0.512	0.514	0.515	0.510	0.520
**I9a_draw17**	0.535	0.507	0.511	0.514	0.509	0.523	0.517	0.525
**I9a_draw18**	0.547	0.507	0.530	0.513	0.510	0.516	0.506	0.505
**I9a_draw19**	0.482	0.483	0.533	0.513	0.510	0.514	0.505	0.518
**I9a_draw20**	0.523	0.544	0.511	0.525	0.514	0.510	0.524	0.526
**I10_draw1**	0.561	0.521	0.522	0.520	0.537	0.526	0.533	0.533
**I10_draw2**	0.551	0.526	0.549	0.544	0.532	0.533	0.532	0.536
**I10_draw3**	0.441	0.508	0.532	0.532	0.522	0.528	0.524	0.528
**I10_draw4**	0.517	0.546	0.567	0.529	0.528	0.529	0.526	0.531
**I10_draw5**	0.555	0.532	0.532	0.537	0.527	0.527	0.527	0.524
**I10_draw6**	0.510	0.551	0.546	0.533	0.523	0.527	0.531	0.525
**I10_draw7**	0.552	0.546	0.513	0.529	0.534	0.531	0.531	0.525
**I10_draw8**	0.520	0.513	0.528	0.520	0.517	0.527	0.527	0.535
**I10_draw9**	0.573	0.578	0.528	0.531	0.530	0.525	0.530	0.529
**I10_draw10**	0.516	0.540	0.542	0.533	0.547	0.528	0.536	0.518
**I10_draw11**	0.517	0.526	0.535	0.544	0.528	0.529	0.523	0.531
**I10_draw12**	0.505	0.521	0.505	0.523	0.520	0.522	0.532	0.530
**I10_draw13**	0.527	0.564	0.547	0.529	0.544	0.524	0.530	0.529
**I10_draw14**	0.521	0.515	0.517	0.520	0.528	0.530	0.526	0.533
**I10_draw15**	0.522	0.538	0.526	0.510	0.514	0.527	0.528	0.525
**I10_draw16**	0.582	0.511	0.511	0.538	0.519	0.519	0.528	0.532
**I10_draw17**	0.485	0.541	0.526	0.537	0.535	0.528	0.525	0.525
**I10_draw18**	0.501	0.556	0.548	0.538	0.530	0.527	0.529	0.521
**I10_draw19**	0.569	0.555	0.526	0.523	0.523	0.527	0.531	0.530
**I10_draw20**	0.488	0.524	0.544	0.518	0.526	0.525	0.524	0.530
**I14_draw1**	0.564	0.534	0.515	0.550	0.546	0.540	0.541	0.550
**I14_draw2**	0.499	0.545	0.551	0.558	0.539	0.537	0.542	0.541
**I14_draw3**	0.576	0.526	0.536	0.525	0.544	0.545	0.545	0.544
**I14_draw4**	0.573	0.565	0.537	0.552	0.529	0.535	0.543	0.543
**I14_draw5**	0.549	0.571	0.568	0.546	0.559	0.543	0.544	0.541
**I14_draw6**	0.589	0.575	0.537	0.556	0.544	0.542	0.538	0.540
**I14_draw7**	0.521	0.563	0.574	0.557	0.539	0.538	0.545	0.542
**I14_draw8**	0.562	0.561	0.524	0.532	0.540	0.548	0.543	0.547
**I14_draw9**	0.603	0.562	0.561	0.533	0.562	0.546	0.542	0.547
**I14_draw10**	0.596	0.522	0.557	0.535	0.535	0.534	0.545	0.544
**I14_draw11**	0.528	0.570	0.549	0.541	0.546	0.540	0.545	0.541
**I14_draw12**	0.498	0.554	0.549	0.546	0.530	0.544	0.542	0.548
**I14_draw13**	0.516	0.536	0.555	0.539	0.535	0.540	0.544	0.541
**I14_draw14**	0.492	0.528	0.549	0.535	0.555	0.536	0.544	0.539
**I14_draw15**	0.539	0.577	0.532	0.560	0.543	0.536	0.544	0.548
**I14_draw16**	0.495	0.561	0.535	0.529	0.554	0.546	0.543	0.543
**I14_draw17**	0.562	0.543	0.552	0.543	0.543	0.543	0.542	0.546
**I14_draw18**	0.564	0.578	0.513	0.549	0.548	0.540	0.545	0.541
**I14_draw19**	0.551	0.553	0.528	0.538	0.547	0.541	0.547	0.542
**I14_draw20**	0.584	0.581	0.541	0.543	0.547	0.549	0.544	0.551
**I15_draw1**	0.583	0.533	0.514	0.531	0.503	0.509	0.504	0.503
**I15_draw2**	0.496	0.511	0.500	0.505	0.508	0.506	0.505	0.507
**I15_draw3**	0.462	0.489	0.505	0.501	0.507	0.504	0.502	0.500
**I15_draw4**	0.480	0.490	0.524	0.484	0.510	0.510	0.503	0.505
**I15_draw5**	0.518	0.500	0.513	0.510	0.510	0.510	0.506	0.506
**I15_draw6**	0.504	0.503	0.512	0.497	0.513	0.507	0.504	0.507
**I15_draw7**	0.490	0.521	0.512	0.514	0.501	0.500	0.506	0.507
**I15_draw8**	0.486	0.481	0.497	0.519	0.504	0.507	0.508	0.493
**I15_draw9**	0.463	0.487	0.503	0.518	0.501	0.506	0.503	0.504
**I15_draw10**	0.460	0.524	0.506	0.529	0.507	0.504	0.507	0.511
**I15_draw11**	0.501	0.505	0.498	0.516	0.501	0.509	0.508	0.506
**I15_draw12**	0.488	0.506	0.499	0.491	0.505	0.500	0.505	0.500
**I15_draw13**	0.508	0.498	0.508	0.513	0.500	0.511	0.505	0.499
**I15_draw14**	0.499	0.505	0.494	0.504	0.494	0.510	0.506	0.509
**I15_draw15**	0.477	0.493	0.512	0.501	0.510	0.507	0.511	0.509
**I15_draw16**	0.542	0.553	0.517	0.518	0.505	0.500	0.502	0.508
**I15_draw17**	0.478	0.508	0.513	0.510	0.507	0.504	0.508	0.515
**I15_draw18**	0.513	0.523	0.527	0.505	0.497	0.494	0.507	0.502
**I15_draw19**	0.460	0.468	0.504	0.512	0.500	0.508	0.506	0.501
**I15_draw20**	0.494	0.474	0.496	0.529	0.500	0.499	0.506	0.514
**I16_draw1**	0.561	0.521	0.508	0.496	0.501	0.503	0.501	0.496
**I16_draw2**	0.551	0.526	0.511	0.505	0.494	0.502	0.499	0.494
**I16_draw3**	0.441	0.508	0.507	0.492	0.509	0.501	0.503	0.498
**I16_draw4**	0.517	0.546	0.504	0.503	0.485	0.504	0.491	0.500
**I16_draw5**	0.555	0.532	0.529	0.505	0.508	0.500	0.498	0.495
**I16_draw6**	0.510	0.551	0.477	0.504	0.495	0.499	0.497	0.503
**I16_draw7**	0.552	0.546	0.509	0.511	0.504	0.500	0.499	0.500
**I16_draw8**	0.520	0.513	0.474	0.473	0.497	0.501	0.497	0.498
**I16_draw9**	0.573	0.578	0.523	0.496	0.495	0.498	0.501	0.499
**I16_draw10**	0.516	0.540	0.506	0.511	0.502	0.501	0.498	0.504
**I16_draw11**	0.517	0.526	0.506	0.505	0.493	0.499	0.506	0.511
**I16_draw12**	0.505	0.521	0.502	0.495	0.506	0.501	0.504	0.491
**I16_draw13**	0.527	0.564	0.496	0.522	0.496	0.502	0.502	0.504
**I16_draw14**	0.521	0.515	0.502	0.490	0.504	0.500	0.502	0.502
**I16_draw15**	0.522	0.538	0.488	0.519	0.508	0.498	0.502	0.506
**I16_draw16**	0.582	0.511	0.510	0.500	0.512	0.500	0.495	0.502
**I16_draw17**	0.485	0.541	0.519	0.521	0.497	0.496	0.500	0.506
**I16_draw18**	0.501	0.556	0.512	0.486	0.507	0.496	0.500	0.498
**I16_draw19**	0.569	0.555	0.523	0.487	0.508	0.500	0.506	0.509
**I16_draw20**	0.488	0.524	0.501	0.491	0.501	0.500	0.498	0.497
**I17_draw1**	0.511	0.520	0.503	0.503	0.514	0.508	0.498	0.503
**I17_draw2**	0.542	0.515	0.510	0.513	0.507	0.510	0.494	0.497
**I17_draw3**	0.489	0.549	0.498	0.512	0.516	0.507	0.499	0.496
**I17_draw4**	0.542	0.527	0.509	0.518	0.503	0.511	0.509	0.491
**I17_draw5**	0.546	0.527	0.520	0.507	0.508	0.506	0.499	0.506
**I17_draw6**	0.550	0.482	0.494	0.510	0.507	0.513	0.503	0.496
**I17_draw7**	0.526	0.503	0.499	0.516	0.510	0.510	0.505	0.497
**I17_draw8**	0.524	0.483	0.497	0.514	0.512	0.507	0.504	0.504
**I17_draw9**	0.498	0.521	0.509	0.506	0.494	0.508	0.498	0.505
**I17_draw10**	0.491	0.513	0.492	0.507	0.506	0.511	0.501	0.490
**I17_draw11**	0.516	0.501	0.520	0.506	0.514	0.511	0.498	0.514
**I17_draw12**	0.473	0.518	0.497	0.512	0.518	0.514	0.499	0.487
**I17_draw13**	0.529	0.506	0.515	0.502	0.506	0.508	0.506	0.494
**I17_draw14**	0.521	0.494	0.525	0.514	0.508	0.513	0.504	0.499
**I17_draw15**	0.551	0.520	0.517	0.488	0.516	0.512	0.500	0.504
**I17_draw16**	0.487	0.500	0.514	0.518	0.507	0.510	0.500	0.501
**I17_draw17**	0.539	0.505	0.508	0.533	0.507	0.510	0.501	0.509
**I17_draw18**	0.531	0.504	0.521	0.498	0.505	0.516	0.503	0.500
**I17_draw19**	0.508	0.500	0.532	0.496	0.504	0.509	0.499	0.505
**I17_draw20**	0.569	0.534	0.527	0.506	0.502	0.502	0.505	0.495
**I18_draw1**	0.460	0.541	0.535	0.530	0.521	0.522	0.524	0.521
**I18_draw2**	0.526	0.526	0.523	0.553	0.528	0.530	0.526	0.524
**I18_draw3**	0.536	0.511	0.541	0.531	0.500	0.524	0.523	0.524
**I18_draw4**	0.521	0.528	0.530	0.529	0.530	0.524	0.527	0.533
**I18_draw5**	0.541	0.529	0.527	0.527	0.531	0.526	0.527	0.521
**I18_draw6**	0.491	0.506	0.508	0.524	0.532	0.526	0.529	0.524
**I18_draw7**	0.523	0.561	0.523	0.538	0.520	0.527	0.529	0.532
**I18_draw8**	0.515	0.540	0.515	0.522	0.532	0.531	0.527	0.525
**I18_draw9**	0.554	0.523	0.537	0.533	0.527	0.525	0.532	0.525
**I18_draw10**	0.530	0.516	0.518	0.527	0.527	0.528	0.522	0.520
**I18_draw11**	0.556	0.546	0.508	0.527	0.534	0.524	0.533	0.527
**I18_draw12**	0.516	0.529	0.511	0.534	0.539	0.526	0.528	0.524
**I18_draw13**	0.495	0.515	0.544	0.524	0.532	0.526	0.528	0.532
**I18_draw14**	0.568	0.529	0.528	0.531	0.519	0.528	0.534	0.525
**I18_draw15**	0.508	0.522	0.532	0.515	0.522	0.525	0.534	0.528

**D6_draw16**	0.452	0.562	0.473	0.502	0.492	0.500	0.507	0.507
**D6_draw17**	0.505	0.508	0.509	0.518	0.494	0.502	0.507	0.510
**D6_draw18**	0.458	0.513	0.517	0.490	0.489	0.511	0.497	0.502
**D6_draw19**	0.531	0.522	0.495	0.500	0.483	0.497	0.498	0.500
**D6_draw20**	0.530	0.497	0.507	0.481	0.498	0.500	0.508	0.503
**MIN**	0.301	0.368	0.370	0.358	0.358	0.372	0.387	0.385
**MAX**	0.600	0.562	0.529	0.520	0.530	0.519	0.510	0.510
**DISPERSION**	0.299	0.194	0.158	0.163	0.171	0.148	0.123	0.125
**MEDIANA**	0.468	0.478	0.475	0.477	0.476	0.475	0.473	0.473

**I18_draw16**	0.546	0.520	0.514	0.536	0.522	0.527	0.528	0.523
**I18_draw17**	0.529	0.482	0.507	0.529	0.521	0.524	0.529	0.532
**I18_draw18**	0.549	0.521	0.509	0.528	0.525	0.526	0.529	0.522
**I18_draw19**	0.550	0.554	0.526	0.530	0.535	0.530	0.526	0.526
**I18_draw20**	0.524	0.518	0.539	0.522	0.522	0.524	0.527	0.529
**I19_draw1**	0.504	0.515	0.502	0.505	0.518	0.506	0.512	0.519
**I19_draw2**	0.505	0.534	0.538	0.502	0.522	0.506	0.508	0.504
**I19_draw3**	0.560	0.509	0.509	0.525	0.508	0.514	0.511	0.507
**I19_draw4**	0.510	0.540	0.521	0.508	0.502	0.506	0.512	0.501
**I19_draw5**	0.521	0.494	0.497	0.488	0.507	0.507	0.508	0.513
**I19_draw6**	0.516	0.484	0.506	0.514	0.517	0.506	0.516	0.509
**I19_draw7**	0.541	0.496	0.524	0.505	0.505	0.509	0.507	0.510
**I19_draw8**	0.508	0.543	0.513	0.497	0.511	0.509	0.506	0.507
**I19_draw9**	0.512	0.530	0.535	0.491	0.508	0.508	0.510	0.509
**I19_draw10**	0.552	0.513	0.519	0.512	0.501	0.514	0.510	0.508
**I19_draw11**	0.482	0.483	0.502	0.498	0.515	0.510	0.508	0.505
**I19_draw12**	0.460	0.501	0.503	0.519	0.495	0.505	0.507	0.506
**I19_draw13**	0.524	0.510	0.501	0.526	0.511	0.511	0.512	0.507
**I19_draw14**	0.514	0.509	0.496	0.514	0.516	0.512	0.512	0.506
**I19_draw15**	0.481	0.523	0.490	0.512	0.519	0.504	0.507	0.514
**I19_draw16**	0.459	0.497	0.494	0.512	0.519	0.510	0.511	0.507
**I19_draw17**	0.562	0.511	0.512	0.491	0.516	0.501	0.512	0.511
**I19_draw18**	0.501	0.482	0.513	0.512	0.519	0.513	0.509	0.511
**I19_draw19**	0.518	0.506	0.512	0.515	0.521	0.519	0.513	0.512
**I19_draw20**	0.501	0.522	0.500	0.503	0.519	0.503	0.508	0.516
**I20_draw1**	0.575	0.500	0.525	0.526	0.517	0.517	0.517	0.519
**I20_draw2**	0.520	0.538	0.549	0.522	0.526	0.516	0.518	0.525
**I20_draw3**	0.559	0.510	0.531	0.519	0.503	0.518	0.517	0.524
**I20_draw4**	0.539	0.519	0.534	0.523	0.506	0.518	0.519	0.521
**I20_draw5**	0.526	0.509	0.486	0.512	0.502	0.526	0.519	0.516
**I20_draw6**	0.502	0.532	0.525	0.514	0.509	0.522	0.520	0.519
**I20_draw7**	0.529	0.537	0.533	0.533	0.518	0.522	0.516	0.515
**I20_draw8**	0.499	0.521	0.513	0.525	0.526	0.525	0.519	0.517
**I20_draw9**	0.502	0.541	0.516	0.508	0.521	0.513	0.517	0.525
**I20_draw10**	0.556	0.527	0.501	0.503	0.518	0.509	0.516	0.520
**I20_draw11**	0.506	0.532	0.494	0.514	0.519	0.513	0.519	0.515
**I20_draw12**	0.509	0.537	0.523	0.523	0.529	0.521	0.520	0.514
**I20_draw13**	0.552	0.551	0.503	0.511	0.511	0.513	0.518	0.514
**I20_draw14**	0.540	0.507	0.504	0.525	0.530	0.514	0.517	0.519
**I20_draw15**	0.498	0.526	0.524	0.514	0.519	0.516	0.521	0.521
**I20_draw16**	0.525	0.516	0.500	0.519	0.526	0.518	0.519	0.518
**I20_draw17**	0.485	0.511	0.507	0.505	0.525	0.508	0.518	0.516
**I20_draw18**	0.526	0.530	0.511	0.525	0.520	0.511	0.524	0.517
**I20_draw19**	0.555	0.530	0.515	0.511	0.520	0.516	0.520	0.521
**I20_draw20**	0.453	0.503	0.515	0.511	0.527	0.520	0.516	0.516
**I21_draw1**	0.561	0.512	0.495	0.521	0.472	0.456	0.529	0.521
**I21_draw2**	0.551	0.509	0.506	0.523	0.481	0.500	0.531	0.530
**I21_draw3**	0.441	0.491	0.530	0.517	0.464	0.465	0.524	0.530
**I21_draw4**	0.517	0.526	0.537	0.527	0.458	0.492	0.533	0.533
**I21_draw5**	0.555	0.538	0.514	0.518	0.494	0.455	0.536	0.532
**I21_draw6**	0.510	0.497	0.533	0.536	0.445	0.474	0.528	0.524
**I21_draw7**	0.552	0.527	0.531	0.530	0.464	0.521	0.531	0.526
**I21_draw8**	0.520	0.525	0.523	0.517	0.473	0.481	0.524	0.527
**I21_draw9**	0.573	0.521	0.528	0.524	0.469	0.501	0.525	0.531
**I21_draw10**	0.516	0.520	0.537	0.517	0.376	0.483	0.525	0.531
**I21_draw11**	0.517	0.518	0.513	0.515	0.419	0.497	0.535	0.527
**I21_draw12**	0.505	0.514	0.535	0.514	0.489	0.476	0.529	0.530
**I21_draw13**	0.527	0.521	0.516	0.522	0.497	0.446	0.518	0.536
**I21_draw14**	0.521	0.504	0.530	0.535	0.464	0.458	0.531	0.523
**I21_draw15**	0.522	0.524	0.509	0.531	0.503	0.475	0.530	0.532
**I21_draw16**	0.582	0.509	0.517	0.517	0.441	0.449	0.529	0.530
**I21_draw17**	0.485	0.504	0.519	0.510	0.477	0.478	0.533	0.526
**I21_draw18**	0.501	0.493	0.504	0.512	0.439	0.483	0.525	0.528
**I21_draw19**	0.569	0.514	0.499	0.522	0.482	0.476	0.532	0.528
**I21_draw20**	0.488	0.509	0.540	0.520	0.467	0.488	0.525	0.525
**I23_draw1**	0.573	0.536	0.534	0.539	0.542	0.537	0.536	0.542
**I23_draw2**	0.591	0.555	0.558	0.546	0.537	0.546	0.539	0.542
**I23_draw3**	0.557	0.538	0.532	0.546	0.538	0.530	0.538	0.542
**I23_draw4**	0.571	0.514	0.536	0.533	0.554	0.546	0.548	0.541
**I23_draw5**	0.574	0.525	0.553	0.548	0.541	0.528	0.541	0.541
**I23_draw6**	0.602	0.549	0.548	0.544	0.538	0.543	0.542	0.542
**I23_draw7**	0.545	0.520	0.539	0.546	0.545	0.541	0.539	0.540
**I23_draw8**	0.552	0.553	0.532	0.541	0.530	0.536	0.550	0.540
**I23_draw9**	0.514	0.544	0.525	0.539	0.534	0.556	0.542	0.539
**I23_draw10**	0.521	0.547	0.532	0.543	0.542	0.530	0.557	0.544
**I23_draw11**	0.566	0.509	0.545	0.537	0.561	0.551	0.549	0.541
**I23_draw12**	0.536	0.554	0.542	0.540	0.553	0.536	0.541	0.541
**I23_draw13**	0.534	0.540	0.543	0.550	0.542	0.545	0.549	0.543
**I23_draw14**	0.544	0.535	0.537	0.533	0.543	0.534	0.549	0.541
**I23_draw15**	0.529	0.580	0.553	0.541	0.547	0.553	0.537	0.545
**I23_draw16**	0.508	0.535	0.550	0.552	0.542	0.539	0.534	0.541
**I23_draw17**	0.525	0.550	0.540	0.536	0.539	0.547	0.542	0.543
**I23_draw18**	0.569	0.558	0.525	0.543	0.536	0.543	0.540	0.543
**I23_draw19**	0.532	0.536	0.539	0.546	0.551	0.545	0.540	0.539
**I23_draw20**	0.523	0.533	0.556	0.543	0.541	0.532	0.542	0.542
**D2_draw1**	0.645	0.647	0.644	0.660	0.657	0.656	0.650	0.655
**D2_draw2**	0.667	0.637	0.647	0.646	0.656	0.651	0.650	0.654
**D2_draw3**	0.648	0.644	0.661	0.651	0.653	0.652	0.653	0.650
**D2_draw4**	0.636	0.669	0.654	0.628	0.659	0.653	0.649	0.651
**D2_draw5**	0.644	0.665	0.663	0.652	0.654	0.658	0.658	0.651
**D2_draw6**	0.653	0.638	0.674	0.654	0.649	0.653	0.654	0.655
**D2_draw7**	0.610	0.635	0.656	0.653	0.643	0.642	0.650	0.651
**D2_draw8**	0.619	0.655	0.647	0.659	0.650	0.645	0.654	0.655
**D2_draw9**	0.664	0.643	0.633	0.639	0.651	0.645	0.652	0.647
**D2_draw10**	0.655	0.657	0.649	0.654	0.648	0.651	0.654	0.649
**D2_draw11**	0.686	0.657	0.633	0.644	0.657	0.656	0.648	0.650
**D2_draw12**	0.679	0.662	0.646	0.658	0.656	0.653	0.648	0.644
**D2_draw13**	0.639	0.651	0.646	0.661	0.661	0.644	0.650	0.653
**D2_draw14**	0.653	0.673	0.656	0.656	0.639	0.648	0.653	0.654
**D2_draw15**	0.644	0.647	0.653	0.654	0.650	0.650	0.656	0.652
**D2_draw16**	0.629	0.621	0.640	0.668	0.652	0.657	0.649	0.652
**D2_draw17**	0.649	0.636	0.648	0.662	0.642	0.653	0.649	0.648
**D2_draw18**	0.675	0.639	0.646	0.658	0.658	0.652	0.654	0.654
**D2_draw19**	0.638	0.626	0.647	0.657	0.648	0.662	0.650	0.650
**D2_draw20**	0.645	0.643	0.648	0.640	0.648	0.656	0.646	0.649
**D5_draw1**	0.614	0.625	0.608	0.606	0.607	0.614	0.622	0.614
**D5_draw2**	0.623	0.628	0.616	0.617	0.602	0.608	0.612	0.612
**D5_draw3**	0.622	0.617	0.622	0.626	0.614	0.606	0.609	0.614
**D5_draw4**	0.656	0.608	0.604	0.604	0.608	0.615	0.621	0.615
**D5_draw5**	0.564	0.624	0.613	0.621	0.607	0.612	0.613	0.614
**D5_draw6**	0.590	0.596	0.617	0.649	0.622	0.602	0.603	0.611
**D5_draw7**	0.636	0.599	0.604	0.613	0.612	0.618	0.615	0.614
**D5_draw8**	0.646	0.588	0.626	0.619	0.620	0.614	0.620	0.613
**D5_draw9**	0.606	0.614	0.619	0.618	0.617	0.624	0.622	0.614
**D5_draw10**	0.583	0.623	0.607	0.624	0.621	0.614	0.611	0.612
**D5_draw11**	0.636	0.628	0.637	0.606	0.602	0.609	0.609	0.621
**D5_draw12**	0.585	0.608	0.625	0.635	0.615	0.620	0.613	0.613
**D5_draw13**	0.601	0.601	0.603	0.618	0.615	0.607	0.612	0.609
**D5_draw14**	0.622	0.612	0.624	0.623	0.625	0.620	0.611	0.619
**D5_draw15**	0.593	0.623	0.623	0.600	0.617	0.619	0.618	0.614
**D5_draw16**	0.587	0.617	0.633	0.614	0.619	0.618	0.605	0.615
**D5_draw17**	0.637	0.631	0.625	0.618	0.621	0.614	0.620	0.615
**D5_draw18**	0.629	0.627	0.600	0.626	0.616	0.625	0.613	0.616
**D5_draw19**	0.596	0.616	0.605	0.614	0.616	0.619	0.608	0.616
**D5_draw20**	0.627	0.589	0.618	0.627	0.630	0.615	0.621	0.611
**D7_draw1**	0.656	0.614	0.600	0.637	0.626	0.623	0.618	0.626
**D7_draw2**	0.685	0.631	0.605	0.623	0.628	0.629	0.615	0.627
**D7_draw3**	0.613	0.607	0.643	0.634	0.628	0.632	0.624	0.627
**D7_draw4**	0.645	0.615	0.627	0.621	0.631	0.629	0.627	0.629
**D7_draw5**	0.635	0.647	0.620	0.628	0.623	0.628	0.629	0.626
**D7_draw6**	0.587	0.637	0.644	0.606	0.628	0.622	0.620	0.627
**D7_draw7**	0.611	0.614	0.626	0.641	0.621	0.618	0.622	0.618
**D7_draw8**	0.671	0.621	0.611	0.597	0.630	0.638	0.625	0.623
**D7_draw9**	0.682	0.653	0.620	0.633	0.627	0.616	0.621	0.627
**D7_draw10**	0.610	0.589	0.635	0.620	0.620	0.635	0.610	0.625
**D7_draw11**	0.642	0.606	0.567	0.618	0.618	0.621	0.625	0.625
**D7_draw12**	0.569	0.611	0.652	0.631	0.618	0.619	0.632	0.624
**D7_draw13**	0.627	0.649	0.622	0.623	0.615	0.625	0.619	0.630
**D7_draw14**	0.619	0.595	0.631	0.619	0.639	0.635	0.628	0.624
**D7_draw15**	0.664	0.620	0.616	0.621	0.619	0.627	0.615	0.628
**D7_draw16**	0.663	0.627	0.611	0.643	0.618	0.625	0.629	0.625
**D7_draw17**	0.544	0.591	0.637	0.622	0.626	0.627	0.627	0.623
**D7_draw18**	0.621	0.638	0.651	0.641	0.628	0.631	0.628	0.622
**D7_draw19**	0.640	0.639	0.630	0.637	0.626	0.634	0.622	0.621
**D7_draw20**	0.605	0.671	0.634	0.619	0.638	0.631	0.623	0.625
**D8_draw1**	0.573	0.564	0.553	0.566	0.546	0.552	0.544	0.553
**D8_draw2**	0.554	0.542	0.547	0.538	0.551	0.553	0.556	0.557
**D8_draw3**	0.622	0.545	0.573	0.551	0.553	0.554	0.539	0.550
**D8_draw4**	0.574	0.570	0.560	0.544	0.552	0.555	0.563	0.551
**D8_draw5**	0.570	0.540	0.562	0.556	0.554	0.554	0.555	0.546
**D8_draw6**	0.551	0.547	0.543	0.539	0.551	0.552	0.556	0.556
**D8_draw7**	0.582	0.542	0.549	0.563	0.552	0.553	0.553	0.543
**D8_draw8**	0.536	0.585	0.546	0.555	0.554	0.554	0.544	0.549
**D8_draw9**	0.557	0.510	0.559	0.556	0.552	0.553	0.546	0.552
**D8_draw10**	0.567	0.549	0.550	0.553	0.567	0.552	0.544	0.553
**D8_draw11**	0.543	0.563	0.551	0.544	0.557	0.555	0.557	0.554
**D8_draw12**	0.593	0.572	0.534	0.546	0.560	0.552	0.557	0.555
**D8_draw13**	0.551	0.567	0.547	0.544	0.553	0.551	0.559	0.554
**D8_draw14**	0.568	0.555	0.544	0.557	0.557	0.552	0.543	0.552
**D8_draw15**	0.585	0.552	0.557	0.557	0.550	0.551	0.553	0.553

**D8_draw16**	0.535	0.546	0.540	0.559	0.551	0.551	0.558	0.554
**D8_draw17**	0.558	0.571	0.550	0.543	0.546	0.553	0.559	0.553
**D8_draw18**	0.559	0.529	0.563	0.553	0.556	0.555	0.546	0.552
**D8_draw19**	0.570	0.563	0.571	0.558	0.543	0.552	0.551	0.555
**D8_draw20**	0.517	0.542	0.548	0.559	0.549	0.551	0.553	0.552
**D9_draw1**	0.606	0.558	0.571	0.575	0.587	0.588	0.583	0.576
**D9_draw2**	0.533	0.577	0.566	0.610	0.577	0.590	0.583	0.577
**D9_draw3**	0.617	0.592	0.595	0.564	0.577	0.584	0.580	0.579
**D9_draw4**	0.597	0.609	0.588	0.581	0.578	0.585	0.581	0.584
**D9_draw5**	0.582	0.621	0.603	0.594	0.584	0.583	0.583	0.574
**D9_draw6**	0.599	0.579	0.595	0.587	0.584	0.588	0.584	0.575
**D9_draw7**	0.621	0.583	0.569	0.577	0.588	0.594	0.582	0.584
**D9_draw8**	0.557	0.603	0.569	0.594	0.588	0.581	0.583	0.586
**D9_draw9**	0.633	0.609	0.588	0.583	0.582	0.583	0.585	0.584
**D9_draw10**	0.603	0.595	0.587	0.588	0.580	0.584	0.586	0.582
**D9_draw11**	0.533	0.604	0.581	0.584	0.583	0.584	0.587	0.578
**D9_draw12**	0.580	0.596	0.575	0.586	0.583	0.583	0.582	0.588
**D9_draw13**	0.559	0.583	0.597	0.585	0.586	0.588	0.582	0.588
**D9_draw14**	0.633	0.582	0.576	0.580	0.586	0.576	0.583	0.581
**D9_draw15**	0.599	0.607	0.574	0.596	0.579	0.585	0.584	0.584
**D9_draw16**	0.577	0.598	0.579	0.587	0.579	0.585	0.585	0.580
**D9_draw17**	0.568	0.587	0.584	0.573	0.588	0.581	0.586	0.581
**D9_draw18**	0.589	0.587	0.572	0.592	0.584	0.575	0.588	0.580
**D9_draw19**	0.567	0.593	0.589	0.571	0.581	0.581	0.584	0.577
**D9_draw20**	0.594	0.545	0.573	0.560	0.579	0.585	0.580	0.589
**D10_draw1**	0.570	0.583	0.550	0.544	0.560	0.550	0.549	0.553
**D10_draw2**	0.556	0.554	0.534	0.558	0.553	0.555	0.554	0.558
**D10_draw3**	0.560	0.535	0.551	0.550	0.552	0.547	0.556	0.556
**D10_draw4**	0.533	0.563	0.557	0.565	0.555	0.553	0.554	0.554
**D10_draw5**	0.588	0.541	0.534	0.554	0.557	0.555	0.549	0.550
**D10_draw6**	0.549	0.532	0.583	0.543	0.558	0.554	0.552	0.559
**D10_draw7**	0.548	0.540	0.570	0.557	0.554	0.554	0.553	0.554
**D10_draw8**	0.553	0.538	0.565	0.564	0.565	0.559	0.548	0.552
**D10_draw9**	0.520	0.566	0.572	0.556	0.555	0.554	0.556	0.552
**D10_draw10**	0.606	0.574	0.551	0.550	0.555	0.553	0.554	0.543
**D10_draw11**	0.573	0.522	0.529	0.547	0.555	0.565	0.553	0.556
**D10_draw12**	0.558	0.548	0.539	0.579	0.543	0.548	0.553	0.555
**D10_draw13**	0.530	0.572	0.550	0.554	0.549	0.551	0.550	0.551
**D10_draw14**	0.547	0.554	0.551	0.546	0.540	0.558	0.553	0.549
**D10_draw15**	0.536	0.555	0.566	0.547	0.563	0.555	0.553	0.544
**D10_draw16**	0.564	0.557	0.569	0.549	0.549	0.554	0.553	0.546
**D10_draw17**	0.544	0.551	0.549	0.551	0.543	0.546	0.550	0.550
**D10_draw18**	0.556	0.594	0.552	0.555	0.549	0.548	0.552	0.548
**D10_draw19**	0.545	0.538	0.570	0.560	0.542	0.550	0.550	0.557
**D10_draw20**	0.587	0.512	0.549	0.534	0.555	0.545	0.552	0.559
**D11_draw1**	0.596	0.581	0.584	0.598	0.590	0.596	0.600	0.597
**D11_draw2**	0.580	0.569	0.583	0.613	0.602	0.591	0.591	0.598
**D11_draw3**	0.604	0.588	0.590	0.588	0.604	0.596	0.591	0.592
**D11_draw4**	0.658	0.598	0.603	0.615	0.598	0.593	0.591	0.590
**D11_draw5**	0.614	0.571	0.612	0.606	0.586	0.600	0.593	0.593
**D11_draw6**	0.616	0.577	0.591	0.592	0.591	0.586	0.591	0.590
**D11_draw7**	0.609	0.565	0.590	0.598	0.583	0.597	0.597	0.593
**D11_draw8**	0.583	0.629	0.596	0.593	0.594	0.589	0.595	0.596
**D11_draw9**	0.588	0.574	0.606	0.597	0.577	0.591	0.584	0.593
**D11_draw10**	0.600	0.566	0.579	0.599	0.597	0.595	0.591	0.592
**D11_draw11**	0.578	0.613	0.600	0.597	0.591	0.593	0.589	0.594
**D11_draw12**	0.669	0.610	0.586	0.593	0.582	0.597	0.592	0.594
**D11_draw13**	0.566	0.601	0.604	0.607	0.592	0.600	0.591	0.595
**D11_draw14**	0.590	0.595	0.595	0.606	0.586	0.593	0.592	0.593
**D11_draw15**	0.597	0.613	0.600	0.600	0.606	0.591	0.595	0.591
**D11_draw16**	0.609	0.574	0.569	0.589	0.597	0.600	0.598	0.590
**D11_draw17**	0.568	0.573	0.590	0.585	0.598	0.595	0.596	0.591
**D11_draw18**	0.554	0.597	0.601	0.589	0.603	0.593	0.593	0.597
**D11_draw19**	0.612	0.631	0.589	0.595	0.592	0.588	0.595	0.594
**D11_draw20**	0.596	0.575	0.600	0.595	0.604	0.593	0.592	0.598
**D12_draw1**	0.519	0.535	0.572	0.567	0.555	0.582	0.570	0.567
**D12_draw2**	0.565	0.593	0.579	0.574	0.559	0.572	0.566	0.569
**D12_draw3**	0.603	0.589	0.602	0.562	0.542	0.570	0.569	0.567
**D12_draw4**	0.537	0.570	0.542	0.562	0.552	0.572	0.561	0.566
**D12_draw5**	0.593	0.560	0.574	0.560	0.566	0.562	0.569	0.568
**D12_draw6**	0.600	0.594	0.570	0.561	0.576	0.564	0.565	0.567
**D12_draw7**	0.595	0.583	0.579	0.563	0.566	0.570	0.562	0.563
**D12_draw8**	0.543	0.613	0.584	0.555	0.563	0.567	0.569	0.566
**D12_draw9**	0.584	0.551	0.575	0.569	0.560	0.561	0.567	0.570
**D12_draw10**	0.595	0.557	0.561	0.550	0.547	0.561	0.563	0.567
**D12_draw11**	0.518	0.578	0.566	0.558	0.571	0.562	0.564	0.564
**D12_draw12**	0.540	0.569	0.568	0.578	0.564	0.566	0.569	0.563
**D12_draw13**	0.578	0.586	0.561	0.557	0.567	0.560	0.566	0.568
**D12_draw14**	0.542	0.555	0.600	0.566	0.569	0.567	0.573	0.569
**D12_draw15**	0.566	0.548	0.574	0.582	0.576	0.560	0.564	0.570
**D12_draw16**	0.553	0.511	0.572	0.596	0.566	0.570	0.571	0.566
**D12_draw17**	0.580	0.563	0.559	0.564	0.563	0.572	0.572	0.566
**D12_draw18**	0.567	0.545	0.558	0.568	0.563	0.575	0.570	0.575
**D12_draw19**	0.554	0.583	0.565	0.579	0.570	0.558	0.569	0.567
**D12_draw20**	0.561	0.563	0.567	0.559	0.554	0.562	0.567	0.563
**D13_draw1**	0.533	0.488	0.511	0.512	0.514	0.501	0.514	0.510
**D13_draw2**	0.503	0.512	0.514	0.489	0.508	0.506	0.505	0.509
**D13_draw3**	0.589	0.495	0.509	0.521	0.511	0.521	0.517	0.505
**D13_draw4**	0.501	0.498	0.514	0.491	0.521	0.503	0.509	0.509
**D13_draw5**	0.518	0.534	0.511	0.512	0.526	0.518	0.508	0.516
**D13_draw6**	0.508	0.505	0.497	0.508	0.483	0.496	0.505	0.514
**D13_draw7**	0.578	0.520	0.540	0.494	0.501	0.513	0.511	0.508
**D13_draw8**	0.490	0.485	0.479	0.527	0.498	0.506	0.508	0.510
**D13_draw9**	0.512	0.482	0.497	0.513	0.506	0.517	0.513	0.511
**D13_draw10**	0.471	0.497	0.496	0.518	0.503	0.517	0.517	0.515
**D13_draw11**	0.498	0.553	0.514	0.505	0.515	0.508	0.509	0.509
**D13_draw12**	0.460	0.524	0.511	0.514	0.515	0.518	0.511	0.506
**D13_draw13**	0.543	0.518	0.508	0.522	0.520	0.506	0.510	0.511
**D13_draw14**	0.485	0.485	0.502	0.518	0.510	0.504	0.510	0.508
**D13_draw15**	0.511	0.508	0.498	0.501	0.514	0.507	0.515	0.514
**D13_draw16**	0.516	0.509	0.504	0.512	0.498	0.505	0.511	0.510
**D13_draw17**	0.562	0.514	0.508	0.489	0.503	0.518	0.511	0.512
**D13_draw18**	0.467	0.475	0.545	0.498	0.512	0.500	0.509	0.510
**D13_draw19**	0.485	0.506	0.516	0.492	0.517	0.502	0.513	0.512
**D13_draw20**	0.480	0.546	0.522	0.503	0.502	0.503	0.508	0.513
**MIN**	0.441	0.468	0.474	0.473	0.376	0.446	0.491	0.487
**MAX**	0.744	0.698	0.690	0.686	0.692	0.688	0.698	0.688
**DISPERSION**	0.303	0.230	0.216	0.213	0.316	0.241	0.208	0.201
**MEDIANA**	0.557	0.552	0.549	0.547	0.548	0.548	0.545	0.545

Supplementary Table H Results of randomly drew values of Polish Diatom Index for lakes (IOJ) grouped by primary studies results: GROUP 2 – good ecological status; GROPU 3 – moderate ecological status.

GROUP 2

	50_valve	100_valve	150_vavle	200_valve	250_valve	300_valve	350_valve	400_valve
**JK_draw1**	0.556	0.621	0.708	0.705	0.699	0.713	0.714	0.709
**JK_draw2**	0.623	0.631	0.680	0.685	0.706	0.703	0.706	0.702
**JK_draw3**	0.636	0.653	0.731	0.718	0.726	0.700	0.710	0.708
**JK_draw4**	0.635	0.566	0.706	0.714	0.728	0.709	0.706	0.715
**JK_draw5**	0.588	0.642	0.672	0.713	0.694	0.711	0.707	0.710
**JK_draw6**	0.560	0.607	0.707	0.709	0.706	0.702	0.702	0.701
**JK_draw7**	0.624	0.600	0.688	0.709	0.697	0.707	0.709	0.702
**JK_draw8**	0.629	0.638	0.719	0.697	0.711	0.700	0.708	0.705
**JK_draw9**	0.718	0.632	0.701	0.679	0.688	0.698	0.712	0.710
**JK_draw10**	0.566	0.564	0.710	0.721	0.709	0.694	0.712	0.703
**JK_draw11**	0.551	0.637	0.682	0.729	0.730	0.707	0.707	0.710
**JK_draw12**	0.659	0.606	0.727	0.707	0.710	0.701	0.708	0.708
**JK_draw13**	0.601	0.637	0.692	0.690	0.695	0.693	0.699	0.713
**JK_draw14**	0.606	0.638	0.736	0.717	0.707	0.700	0.707	0.706
**JK_draw15**	0.644	0.591	0.717	0.714	0.712	0.724	0.707	0.709
**JK_draw16**	0.619	0.662	0.678	0.700	0.717	0.710	0.708	0.709
**JK_draw17**	0.675	0.635	0.716	0.736	0.724	0.716	0.713	0.715
**JK_draw18**	0.603	0.613	0.666	0.707	0.714	0.701	0.725	0.705
**JK_draw19**	0.618	0.643	0.694	0.702	0.706	0.713	0.704	0.702
**JK_draw20**	0.613	0.649	0.753	0.715	0.691	0.715	0.711	0.707

GROUP 3

	50_valve	100_valve	150_vavle	200_valve	250_valve	300_valve	350_valve	400_valve
**JD2_draw1**	0.480	0.485	0.484	0.495	0.490	0.486	0.500	0.496
**JD2_draw2**	0.505	0.512	0.491	0.496	0.492	0.477	0.505	0.489
**JD2_draw3**	0.522	0.481	0.511	0.498	0.490	0.494	0.500	0.488
**JD2_draw4**	0.546	0.499	0.487	0.490	0.497	0.503	0.496	0.490
**JD2_draw5**	0.451	0.487	0.494	0.496	0.495	0.482	0.507	0.495
**JD2_draw6**	0.531	0.503	0.485	0.497	0.492	0.499	0.483	0.484
**JD2_draw7**	0.493	0.493	0.506	0.497	0.497	0.504	0.494	0.496
**JD2_draw8**	0.490	0.519	0.508	0.505	0.503	0.493	0.495	0.492
**JD2_draw9**	0.461	0.508	0.487	0.488	0.490	0.496	0.487	0.495
**JD2_draw10**	0.498	0.500	0.483	0.491	0.495	0.490	0.503	0.493
**JD2_draw11**	0.450	0.480	0.492	0.499	0.493	0.497	0.491	0.494
**JD2_draw12**	0.527	0.498	0.466	0.500	0.497	0.501	0.498	0.484
**JD2_draw13**	0.483	0.483	0.506	0.501	0.496	0.496	0.514	0.496
**JD2_draw14**	0.458	0.499	0.508	0.499	0.496	0.510	0.487	0.506
**JD2_draw15**	0.492	0.499	0.494	0.493	0.497	0.504	0.493	0.501
**JD2_draw16**	0.476	0.509	0.471	0.493	0.493	0.483	0.496	0.491
**JD2_draw17**	0.510	0.477	0.498	0.502	0.497	0.493	0.489	0.492
**JD2_draw18**	0.548	0.495	0.482	0.493	0.488	0.490	0.505	0.485
**JD2_draw19**	0.459	0.500	0.503	0.485	0.492	0.488	0.510	0.489
**JD2_draw20**	0.505	0.516	0.482	0.494	0.496	0.491	0.501	0.507

**JB_draw1**	0.557	0.621	0.719	0.691	0.699	0.708	0.736	0.695
**JB_draw2**	0.624	0.632	0.730	0.706	0.714	0.700	0.721	0.688
**JB_draw3**	0.637	0.653	0.742	0.707	0.712	0.734	0.718	0.700
**JB_draw4**	0.635	0.566	0.693	0.714	0.697	0.696	0.706	0.702
**JB_draw5**	0.588	0.642	0.767	0.721	0.714	0.698	0.689	0.688
**JB_draw6**	0.561	0.608	0.697	0.736	0.700	0.690	0.732	0.712
**JB_draw7**	0.624	0.600	0.670	0.724	0.701	0.690	0.690	0.679
**JB_draw8**	0.630	0.639	0.653	0.699	0.712	0.718	0.706	0.715
**JB_draw9**	0.718	0.632	0.742	0.726	0.685	0.718	0.730	0.722
**JB_draw10**	0.566	0.565	0.722	0.758	0.702	0.742	0.698	0.706
**JB_draw11**	0.551	0.637	0.625	0.710	0.700	0.729	0.693	0.716
**JB_draw12**	0.659	0.606	0.669	0.700	0.695	0.732	0.683	0.729
**JB_draw13**	0.601	0.638	0.714	0.717	0.716	0.714	0.722	0.715
**JB_draw14**	0.606	0.638	0.739	0.693	0.716	0.702	0.684	0.677
**JB_draw15**	0.645	0.591	0.711	0.725	0.702	0.660	0.711	0.721
**JB_draw16**	0.619	0.662	0.590	0.737	0.700	0.713	0.711	0.705
**JB_draw17**	0.675	0.635	0.709	0.703	0.719	0.714	0.700	0.691
**JB_draw18**	0.603	0.613	0.739	0.711	0.712	0.722	0.699	0.709
**JB_draw19**	0.618	0.643	0.723	0.736	0.732	0.730	0.718	0.735
**JB_draw20**	0.614	0.649	0.693	0.690	0.704	0.690	0.715	0.713
**JD1_draw1**	0.780	0.738	0.742	0.708	0.721	0.711	0.713	0.724
**JD1_draw2**	0.728	0.756	0.700	0.722	0.712	0.725	0.730	0.721
**JD1_draw3**	0.758	0.741	0.719	0.715	0.712	0.728	0.720	0.719
**JD1_draw4**	0.757	0.705	0.724	0.739	0.735	0.740	0.721	0.724
**JD1_draw5**	0.757	0.729	0.739	0.714	0.713	0.717	0.722	0.726
**JD1_draw6**	0.652	0.699	0.735	0.719	0.724	0.728	0.726	0.720
**JD1_draw7**	0.733	0.702	0.757	0.723	0.728	0.717	0.729	0.724
**JD1_draw8**	0.709	0.746	0.728	0.710	0.709	0.722	0.719	0.722
**JD1_draw9**	0.745	0.705	0.725	0.731	0.709	0.721	0.716	0.724
**JD1_draw10**	0.670	0.715	0.726	0.718	0.719	0.721	0.721	0.718
**JD1_draw11**	0.707	0.684	0.705	0.727	0.718	0.717	0.720	0.725
**JD1_draw12**	0.707	0.727	0.714	0.730	0.738	0.722	0.723	0.724
**JD1_draw13**	0.733	0.709	0.738	0.731	0.717	0.718	0.719	0.717
**JD1_draw14**	0.732	0.696	0.717	0.712	0.708	0.725	0.724	0.723
**JD1_draw15**	0.703	0.711	0.725	0.720	0.716	0.731	0.719	0.724
**JD1_draw16**	0.757	0.704	0.711	0.732	0.744	0.716	0.728	0.725
**JD1_draw17**	0.687	0.714	0.691	0.739	0.736	0.724	0.721	0.721
**JD1_draw18**	0.725	0.737	0.721	0.729	0.718	0.723	0.722	0.721
**JD1_draw19**	0.730	0.700	0.734	0.748	0.718	0.712	0.719	0.725
**JD1_draw20**	0.757	0.715	0.728	0.727	0.718	0.722	0.721	0.724
**JD7_draw1**	0.722	0.727	0.701	0.732	0.711	0.723	0.720	0.708
**JD7_draw2**	0.671	0.756	0.731	0.710	0.717	0.716	0.720	0.723
**JD7_draw3**	0.670	0.739	0.727	0.701	0.714	0.716	0.724	0.716
**JD7_draw4**	0.729	0.724	0.709	0.722	0.712	0.718	0.713	0.719
**JD7_draw5**	0.758	0.726	0.711	0.697	0.710	0.724	0.718	0.715
**JD7_draw6**	0.707	0.680	0.703	0.693	0.716	0.712	0.711	0.723
**JD7_draw7**	0.732	0.703	0.704	0.722	0.705	0.721	0.714	0.717
**JD7_draw8**	0.738	0.737	0.722	0.703	0.718	0.733	0.715	0.716
**JD7_draw9**	0.745	0.733	0.737	0.733	0.736	0.724	0.719	0.725
**JD7_draw10**	0.746	0.688	0.726	0.705	0.725	0.714	0.722	0.717
**JD7_draw11**	0.695	0.755	0.725	0.724	0.741	0.710	0.721	0.718
**JD7_draw12**	0.699	0.681	0.718	0.738	0.709	0.733	0.719	0.719
**JD7_draw13**	0.767	0.771	0.724	0.707	0.710	0.715	0.713	0.715
**JD7_draw14**	0.722	0.727	0.706	0.709	0.716	0.719	0.715	0.719
**JD7_draw15**	0.658	0.746	0.755	0.711	0.732	0.711	0.723	0.720
**JD7_draw16**	0.761	0.731	0.696	0.722	0.721	0.716	0.725	0.715
**JD7_draw17**	0.762	0.752	0.700	0.727	0.715	0.720	0.722	0.720
**JD7_draw18**	0.774	0.772	0.707	0.711	0.715	0.708	0.720	0.718
**JD7_draw19**	0.711	0.727	0.736	0.699	0.711	0.730	0.722	0.722
**JD7_draw20**	0.733	0.743	0.720	0.709	0.710	0.714	0.716	0.721
**JD8_draw1**	0.638	0.619	0.637	0.624	0.612	0.617	0.615	0.622
**JD8_draw2**	0.604	0.617	0.636	0.592	0.617	0.623	0.617	0.618
**JD8_draw3**	0.648	0.619	0.608	0.607	0.619	0.606	0.626	0.618
**JD8_draw4**	0.582	0.606	0.634	0.642	0.625	0.622	0.620	0.615
**JD8_draw5**	0.574	0.622	0.598	0.614	0.614	0.626	0.614	0.616
**JD8_draw6**	0.656	0.611	0.603	0.617	0.615	0.625	0.612	0.616
**JD8_draw7**	0.573	0.609	0.641	0.604	0.610	0.619	0.618	0.616
**JD8_draw8**	0.600	0.573	0.657	0.635	0.617	0.621	0.616	0.617
**JD8_draw9**	0.565	0.601	0.606	0.644	0.610	0.616	0.619	0.622
**JD8_draw10**	0.624	0.587	0.656	0.634	0.622	0.616	0.616	0.618
**JD8_draw11**	0.580	0.645	0.618	0.605	0.596	0.619	0.611	0.619
**JD8_draw12**	0.582	0.612	0.593	0.601	0.620	0.617	0.617	0.611
**JD8_draw13**	0.622	0.588	0.608	0.633	0.636	0.630	0.618	0.616
**JD8_draw14**	0.651	0.617	0.610	0.629	0.621	0.617	0.616	0.619
**JD8_draw15**	0.560	0.598	0.656	0.596	0.610	0.617	0.620	0.615
**JD8_draw16**	0.618	0.600	0.611	0.607	0.618	0.619	0.616	0.614
**JD8_draw17**	0.598	0.596	0.622	0.596	0.628	0.618	0.623	0.618
**JD8_draw18**	0.607	0.634	0.628	0.617	0.620	0.616	0.618	0.617
**JD8_draw19**	0.582	0.647	0.653	0.609	0.613	0.623	0.620	0.613
**JD8_draw20**	0.626	0.568	0.643	0.614	0.614	0.612	0.610	0.615
**JD9_draw1**	0.697	0.699	0.649	0.661	0.674	0.647	0.643	0.657
**JD9_draw2**	0.679	0.653	0.665	0.690	0.675	0.647	0.652	0.652
**JD9_draw3**	0.634	0.698	0.678	0.660	0.682	0.648	0.650	0.652
**JD9_draw4**	0.692	0.623	0.649	0.654	0.661	0.644	0.650	0.662
**JD9_draw5**	0.695	0.637	0.692	0.636	0.654	0.669	0.654	0.652
**JD9_draw6**	0.613	0.630	0.652	0.657	0.653	0.653	0.643	0.645
**JD9_draw7**	0.614	0.673	0.677	0.635	0.636	0.666	0.656	0.646
**JD9_draw8**	0.709	0.664	0.673	0.646	0.664	0.666	0.650	0.644
**JD9_draw9**	0.652	0.694	0.681	0.647	0.649	0.648	0.644	0.654
**JD9_draw10**	0.643	0.702	0.658	0.662	0.659	0.653	0.644	0.658
**JD9_draw11**	0.593	0.644	0.626	0.656	0.653	0.659	0.633	0.658
**JD9_draw12**	0.640	0.615	0.648	0.673	0.645	0.660	0.661	0.656
**JD9_draw13**	0.642	0.634	0.681	0.647	0.652	0.646	0.648	0.668
**JD9_draw14**	0.694	0.678	0.651	0.677	0.663	0.667	0.656	0.655
**JD9_draw15**	0.642	0.629	0.677	0.667	0.662	0.662	0.648	0.649
**JD9_draw16**	0.660	0.571	0.659	0.652	0.660	0.659	0.659	0.653
**JD9_draw17**	0.685	0.649	0.647	0.669	0.643	0.663	0.650	0.653
**JD9_draw18**	0.609	0.666	0.643	0.658	0.648	0.666	0.655	0.647
**JD9_draw19**	0.641	0.648	0.688	0.652	0.642	0.657	0.640	0.643
**JD9_draw20**	0.627	0.613	0.662	0.645	0.660	0.646	0.652	0.652
**JD12_draw1**	0.604	0.660	0.648	0.612	0.659	0.649	0.636	0.637
**JD12_draw2**	0.600	0.622	0.643	0.638	0.647	0.634	0.642	0.647
**JD12_draw3**	0.605	0.622	0.642	0.650	0.654	0.637	0.642	0.646
**JD12_draw4**	0.619	0.689	0.628	0.638	0.638	0.629	0.636	0.643
**JD12_draw5**	0.636	0.647	0.637	0.639	0.631	0.643	0.639	0.639
**JD12_draw6**	0.629	0.629	0.603	0.640	0.636	0.634	0.647	0.640
**JD12_draw7**	0.624	0.669	0.656	0.631	0.641	0.635	0.642	0.638
**JD12_draw8**	0.607	0.668	0.621	0.641	0.644	0.646	0.641	0.634
**JD12_draw9**	0.643	0.599	0.651	0.640	0.631	0.650	0.647	0.635
**JD12_draw10**	0.699	0.626	0.618	0.649	0.657	0.647	0.641	0.637
**JD12_draw11**	0.668	0.690	0.656	0.629	0.661	0.643	0.633	0.639
**JD12_draw12**	0.655	0.681	0.659	0.617	0.644	0.633	0.645	0.638
**JD12_draw13**	0.601	0.614	0.628	0.628	0.641	0.646	0.640	0.641
**JD12_draw14**	0.672	0.609	0.622	0.669	0.626	0.635	0.644	0.636
**JD12_draw15**	0.645	0.630	0.630	0.668	0.624	0.636	0.644	0.639
**JD12_draw16**	0.661	0.677	0.640	0.623	0.635	0.640	0.636	0.635
**JD12_draw17**	0.627	0.632	0.633	0.627	0.635	0.638	0.643	0.640
**JD12_draw18**	0.681	0.635	0.621	0.648	0.633	0.632	0.640	0.642
**JD12_draw19**	0.688	0.662	0.667	0.622	0.635	0.646	0.642	0.634
**JD12_draw20**	0.647	0.655	0.630	0.632	0.647	0.628	0.638	0.640
**MIN**	0.551	0.564	0.590	0.592	0.596	0.606	0.610	0.611
**MAX**	0.780	0.772	0.767	0.758	0.744	0.742	0.736	0.735
**DISPERSION**	0.229	0.208	0.177	0.165	0.148	0.136	0.126	0.124
**MEDIANA**	0.643	0.644	0.690	0.699	0.699	0.700	0.703	0.702

**JD3_draw1**	0.431	0.473	0.495	0.494	0.510	0.490	0.504	0.504
**JD3_draw2**	0.490	0.465	0.490	0.522	0.501	0.488	0.498	0.498
**JD3_draw3**	0.481	0.465	0.475	0.500	0.481	0.520	0.505	0.505
**JD3_draw4**	0.463	0.495	0.519	0.497	0.491	0.490	0.488	0.488
**JD3_draw5**	0.467	0.454	0.492	0.482	0.479	0.499	0.495	0.495
**JD3_draw6**	0.500	0.523	0.489	0.511	0.518	0.497	0.500	0.500
**JD3_draw7**	0.498	0.520	0.483	0.492	0.490	0.512	0.477	0.477
**JD3_draw8**	0.478	0.514	0.483	0.519	0.486	0.495	0.492	0.492
**JD3_draw9**	0.478	0.475	0.484	0.502	0.499	0.501	0.505	0.505
**JD3_draw10**	0.465	0.524	0.510	0.490	0.486	0.505	0.509	0.509
**JD3_draw11**	0.467	0.524	0.491	0.493	0.485	0.489	0.489	0.489
**JD3_draw12**	0.457	0.476	0.480	0.496	0.506	0.512	0.492	0.492
**JD3_draw13**	0.482	0.537	0.476	0.506	0.485	0.502	0.505	0.505
**JD3_draw14**	0.472	0.500	0.490	0.496	0.474	0.498	0.512	0.512
**JD3_draw15**	0.458	0.493	0.484	0.512	0.488	0.490	0.499	0.499
**JD3_draw16**	0.522	0.521	0.506	0.477	0.501	0.493	0.508	0.508
**JD3_draw17**	0.535	0.504	0.500	0.506	0.492	0.490	0.491	0.491
**JD3_draw18**	0.542	0.492	0.463	0.482	0.522	0.496	0.488	0.488
**JD3_draw19**	0.452	0.497	0.500	0.515	0.514	0.499	0.502	0.502
**JD3_draw20**	0.531	0.534	0.470	0.508	0.511	0.498	0.502	0.502
**JD4_draw1**	0.494	0.485	0.496	0.503	0.517	0.513	0.506	0.515
**JD4_draw2**	0.493	0.534	0.485	0.512	0.492	0.513	0.515	0.514
**JD4_draw3**	0.533	0.509	0.494	0.493	0.497	0.508	0.509	0.514
**JD4_draw4**	0.486	0.481	0.519	0.512	0.510	0.508	0.509	0.504
**JD4_draw5**	0.528	0.515	0.512	0.509	0.508	0.503	0.509	0.514
**JD4_draw6**	0.521	0.488	0.517	0.514	0.515	0.511	0.511	0.511
**JD4_draw7**	0.521	0.546	0.489	0.493	0.513	0.512	0.506	0.517
**JD4_draw8**	0.544	0.515	0.507	0.504	0.520	0.504	0.508	0.508
**JD4_draw9**	0.493	0.535	0.496	0.514	0.507	0.507	0.513	0.521
**JD4_draw10**	0.492	0.494	0.507	0.496	0.509	0.514	0.511	0.507
**JD4_draw11**	0.470	0.500	0.503	0.514	0.510	0.507	0.508	0.507
**JD4_draw12**	0.487	0.491	0.497	0.508	0.515	0.510	0.504	0.505
**JD4_draw13**	0.461	0.526	0.542	0.504	0.511	0.509	0.509	0.510
**JD4_draw14**	0.527	0.516	0.526	0.503	0.511	0.510	0.510	0.506
**JD4_draw15**	0.483	0.506	0.520	0.494	0.515	0.508	0.510	0.517
**JD4_draw16**	0.596	0.531	0.508	0.525	0.499	0.510	0.511	0.507
**JD4_draw17**	0.510	0.496	0.479	0.505	0.529	0.508	0.509	0.505
**JD4_draw18**	0.517	0.488	0.519	0.491	0.493	0.499	0.519	0.524
**JD4_draw19**	0.583	0.503	0.511	0.486	0.518	0.509	0.513	0.508
**JD4_draw20**	0.545	0.548	0.508	0.514	0.505	0.513	0.508	0.500
**JD5_draw1**	0.585	0.550	0.565	0.576	0.575	0.584	0.587	0.585
**JD5_draw2**	0.653	0.560	0.594	0.587	0.595	0.591	0.584	0.584
**JD5_draw3**	0.563	0.614	0.576	0.595	0.596	0.581	0.579	0.588
**JD5_draw4**	0.633	0.592	0.595	0.583	0.580	0.596	0.598	0.586
**JD5_draw5**	0.599	0.562	0.570	0.581	0.592	0.601	0.584	0.581
**JD5_draw6**	0.552	0.617	0.559	0.591	0.588	0.587	0.575	0.583
**JD5_draw7**	0.560	0.582	0.605	0.590	0.597	0.597	0.595	0.588
**JD5_draw8**	0.589	0.570	0.600	0.597	0.567	0.588	0.595	0.583
**JD5_draw9**	0.641	0.586	0.597	0.596	0.572	0.592	0.588	0.581
**JD5_draw10**	0.528	0.577	0.607	0.569	0.585	0.581	0.588	0.584
**JD5_draw11**	0.617	0.589	0.580	0.589	0.605	0.581	0.574	0.585
**JD5_draw12**	0.602	0.596	0.594	0.583	0.581	0.580	0.576	0.586
**JD5_draw13**	0.589	0.581	0.573	0.606	0.588	0.583	0.587	0.584
**JD5_draw14**	0.591	0.581	0.591	0.587	0.590	0.592	0.584	0.582
**JD5_draw15**	0.589	0.634	0.597	0.568	0.585	0.579	0.585	0.588
**JD5_draw16**	0.625	0.603	0.599	0.578	0.592	0.587	0.592	0.584
**JD5_draw17**	0.619	0.578	0.582	0.611	0.587	0.591	0.577	0.588
**JD5_draw18**	0.593	0.588	0.566	0.598	0.567	0.576	0.576	0.587
**JD5_draw19**	0.551	0.571	0.577	0.590	0.583	0.569	0.589	0.581
**JD5_draw20**	0.577	0.595	0.591	0.584	0.578	0.594	0.595	0.589
**JD6_draw1**	0.403	0.411	0.408	0.416	0.408	0.409	0.410	0.409
**JD6_draw2**	0.410	0.400	0.417	0.406	0.405	0.414	0.408	0.411
**JD6_draw3**	0.427	0.406	0.412	0.408	0.412	0.403	0.410	0.409
**JD6_draw4**	0.397	0.410	0.411	0.408	0.405	0.409	0.411	0.410
**JD6_draw5**	0.394	0.417	0.412	0.411	0.410	0.408	0.409	0.411
**JD6_draw6**	0.393	0.405	0.408	0.415	0.409	0.411	0.411	0.410
**JD6_draw7**	0.399	0.410	0.413	0.411	0.408	0.413	0.409	0.410
**JD6_draw8**	0.406	0.401	0.405	0.408	0.412	0.408	0.411	0.410
**JD6_draw9**	0.417	0.412	0.406	0.411	0.410	0.412	0.412	0.410
**JD6_draw10**	0.401	0.411	0.415	0.405	0.406	0.411	0.409	0.411
**JD6_draw11**	0.417	0.410	0.413	0.407	0.410	0.407	0.408	0.410
**JD6_draw12**	0.428	0.410	0.403	0.417	0.412	0.409	0.407	0.409
**JD6_draw13**	0.393	0.405	0.405	0.403	0.412	0.411	0.408	0.409
**JD6_draw14**	0.432	0.416	0.405	0.413	0.411	0.409	0.412	0.410
**JD6_draw15**	0.393	0.412	0.397	0.407	0.408	0.410	0.410	0.409
**JD6_draw16**	0.407	0.400	0.397	0.408	0.410	0.409	0.410	0.411
**JD6_draw17**	0.421	0.429	0.408	0.411	0.414	0.411	0.409	0.410
**JD6_draw18**	0.439	0.410	0.407	0.414	0.403	0.412	0.407	0.409
**JD6_draw19**	0.406	0.421	0.415	0.411	0.408	0.413	0.412	0.409
**JD6_draw20**	0.407	0.413	0.413	0.417	0.410	0.409	0.411	0.409
**JD10_draw1**	0.535	0.512	0.500	0.522	0.493	0.521	0.511	0.514
**JD10_draw2**	0.505	0.544	0.492	0.508	0.518	0.501	0.510	0.503
**JD10_draw3**	0.488	0.506	0.513	0.504	0.520	0.520	0.513	0.515
**JD10_draw4**	0.516	0.490	0.518	0.525	0.513	0.509	0.509	0.509
**JD10_draw5**	0.500	0.527	0.487	0.496	0.509	0.501	0.521	0.513
**JD10_draw6**	0.580	0.556	0.508	0.513	0.481	0.515	0.518	0.514
**JD10_draw7**	0.543	0.499	0.502	0.525	0.527	0.508	0.513	0.517
**JD10_draw8**	0.570	0.505	0.493	0.495	0.522	0.521	0.517	0.514
**JD10_draw9**	0.489	0.512	0.527	0.523	0.522	0.500	0.525	0.511
**JD10_draw10**	0.443	0.488	0.544	0.509	0.514	0.503	0.516	0.511
**JD10_draw11**	0.447	0.541	0.524	0.509	0.526	0.504	0.504	0.512
**JD10_draw12**	0.543	0.545	0.530	0.503	0.517	0.512	0.505	0.515
**JD10_draw13**	0.459	0.538	0.493	0.530	0.500	0.524	0.508	0.510
**JD10_draw14**	0.502	0.523	0.488	0.504	0.489	0.521	0.516	0.505
**JD10_draw15**	0.509	0.470	0.508	0.483	0.488	0.503	0.511	0.509
**JD10_draw16**	0.535	0.502	0.514	0.516	0.505	0.504	0.504	0.509
**JD10_draw17**	0.527	0.500	0.490	0.536	0.514	0.518	0.517	0.512
**JD10_draw18**	0.524	0.542	0.486	0.525	0.535	0.504	0.506	0.511
**JD10_draw19**	0.458	0.500	0.500	0.486	0.522	0.513	0.511	0.510
**JD10_draw20**	0.437	0.480	0.501	0.522	0.497	0.515	0.502	0.512
**JD11_draw1**	0.596	0.561	0.555	0.565	0.558	0.576	0.567	0.551
**JD11_draw2**	0.531	0.551	0.592	0.575	0.554	0.574	0.557	0.583
**JD11_draw3**	0.560	0.542	0.546	0.547	0.580	0.546	0.565	0.559
**JD11_draw4**	0.567	0.550	0.559	0.542	0.556	0.548	0.565	0.563
**JD11_draw5**	0.571	0.580	0.568	0.544	0.574	0.560	0.568	0.566
**JD11_draw6**	0.579	0.580	0.547	0.559	0.561	0.550	0.569	0.552
**JD11_draw7**	0.565	0.575	0.577	0.564	0.548	0.551	0.548	0.551
**JD11_draw8**	0.594	0.548	0.563	0.564	0.554	0.523	0.550	0.552
**JD11_draw9**	0.581	0.563	0.575	0.562	0.582	0.558	0.552	0.541
**JD11_draw10**	0.545	0.565	0.566	0.577	0.519	0.571	0.548	0.550
**JD11_draw11**	0.544	0.545	0.575	0.570	0.554	0.579	0.558	0.565
**JD11_draw12**	0.604	0.562	0.559	0.552	0.580	0.555	0.574	0.530
**JD11_draw13**	0.548	0.557	0.574	0.550	0.557	0.557	0.548	0.579
**JD11_draw14**	0.629	0.559	0.577	0.553	0.576	0.557	0.576	0.534
**JD11_draw15**	0.540	0.567	0.554	0.571	0.549	0.552	0.583	0.582
**JD11_draw16**	0.528	0.541	0.572	0.584	0.558	0.557	0.563	0.549
**JD11_draw17**	0.590	0.542	0.554	0.550	0.571	0.561	0.548	0.556
**JD11_draw18**	0.585	0.550	0.574	0.540	0.537	0.556	0.584	0.569
**JD11_draw19**	0.525	0.554	0.553	0.574	0.564	0.553	0.560	0.566
**JD11_draw20**	0.570	0.551	0.559	0.556	0.571	0.560	0.540	0.586
**JD13_draw1**	0.500	0.502	0.482	0.511	0.498	0.505	0.500	0.494
**JD13_draw2**	0.523	0.458	0.500	0.484	0.499	0.496	0.500	0.492
**JD13_draw3**	0.478	0.501	0.523	0.502	0.495	0.498	0.498	0.489
**JD13_draw4**	0.499	0.480	0.491	0.509	0.500	0.485	0.494	0.496
**JD13_draw5**	0.462	0.517	0.498	0.490	0.497	0.498	0.499	0.497
**JD13_draw6**	0.534	0.498	0.489	0.495	0.501	0.501	0.494	0.497
**JD13_draw7**	0.457	0.505	0.497	0.482	0.502	0.500	0.491	0.493
**JD13_draw8**	0.508	0.494	0.473	0.505	0.493	0.491	0.495	0.493
**JD13_draw9**	0.523	0.498	0.506	0.477	0.497	0.502	0.492	0.491
**JD13_draw10**	0.522	0.477	0.475	0.498	0.491	0.492	0.490	0.496
**JD13_draw11**	0.471	0.500	0.485	0.491	0.508	0.489	0.503	0.494
**JD13_draw12**	0.475	0.496	0.482	0.490	0.502	0.497	0.499	0.502
**JD13_draw13**	0.468	0.498	0.477	0.514	0.491	0.497	0.496	0.490
**JD13_draw14**	0.484	0.518	0.515	0.516	0.501	0.499	0.490	0.497
**JD13_draw15**	0.475	0.485	0.500	0.505	0.503	0.494	0.495	0.489
**JD13_draw16**	0.450	0.508	0.490	0.499	0.487	0.492	0.494	0.492
**JD13_draw17**	0.465	0.482	0.503	0.487	0.487	0.490	0.496	0.494
**JD13_draw18**	0.469	0.498	0.484	0.492	0.488	0.489	0.493	0.494
**JD13_draw19**	0.507	0.507	0.475	0.511	0.480	0.495	0.491	0.498
**JD13_draw20**	0.498	0.478	0.513	0.486	0.505	0.498	0.492	0.487
**MIN**	0.393	0.400	0.397	0.403	0.403	0.403	0.407	0.409
**MAX**	0.653	0.634	0.607	0.611	0.605	0.601	0.598	0.589
**DISPERSION**	0.259	0.234	0.209	0.207	0.202	0.198	0.192	0.180
**MEDIANA**	0.504	0.505	0.500	0.504	0.502	0.504	0.506	0.505
